# Enantioselective Formal Synthesis of (−)-Aflatoxin B_2_ Enabled by Pd-Catalyzed Carboetherification of 2,3-Dihydrofuran

**DOI:** 10.1021/acs.orglett.5c02618

**Published:** 2025-07-18

**Authors:** Max Kadarauch, Robert J. Phipps

**Affiliations:** Yusuf Hamied Department of Chemistry, 2152 University of Cambridge, Lensfield Road, Cambridge CB2 1EW, United Kingdom

## Abstract

We report a short formal synthesis of the natural product (−)-aflatoxin B_2_ enabled by a highly enantioselective Pd/sSPhos-catalyzed carboetherification reaction of 2,3-dihydrofuran; we also describe the application of the key reaction to several other substrates. This key step enantioselectively forges the complex tricyclic core of the natural product directly from commercially available substrates in 99% ee. An enabling aspect is that our chiral phosphine ligand sSPhos, which operates through electrostatically directed catalysis, permits a substituent to be incorporated at the 4 position of the resulting tetrahydrobenzofuran: (−)-aflatoxin B_2_ and other natural products based on this polycyclic motif also contain a substituent in this position. Post-functionalization completes one of the most concise (−)-aflatoxin B_2_ formal syntheses reported to date while also delivering the key intermediate with the highest enantiomeric excess. We also demonstrate sSPhos to be effective in an analogous enantioselective carboamination reaction.

## Graphical abstract

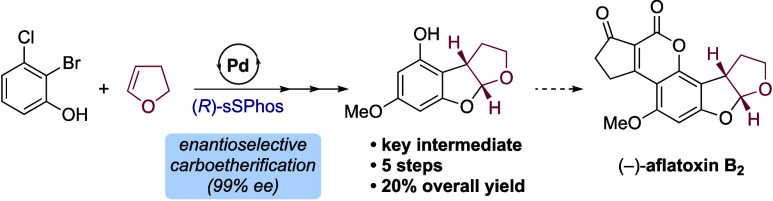

Mycotoxins are secondary metabolites produced by fungi, which are dangerous to humans and animals when found in colonized crops.^1^ Aflatoxins are a family of mycotoxins of particular potency: responsible for many instances of human and animal death, they include some of the most carcinogenic substances known.^2^ The aflatoxins are characterized by their pentacyclic ring system, which includes fused dihydrofurans with adjacent stereocenters (Scheme 1A and B).^3^ Aflatoxins B_1_ and B_2_ differ from G_1_ and G_2_ in their E rings: the former contain a cyclopentenone, whereas the latter feature a lactone motif. Aflatoxins M_1_ and M_2_, meanwhile, contain an alcohol stereocenter between the B and C rings while sharing the cyclopentenone E ring of aflatoxins B_1_ and B_2_. These natural products present challenging targets, inspiring creative synthetic efforts.^4^

**Scheme 1:**
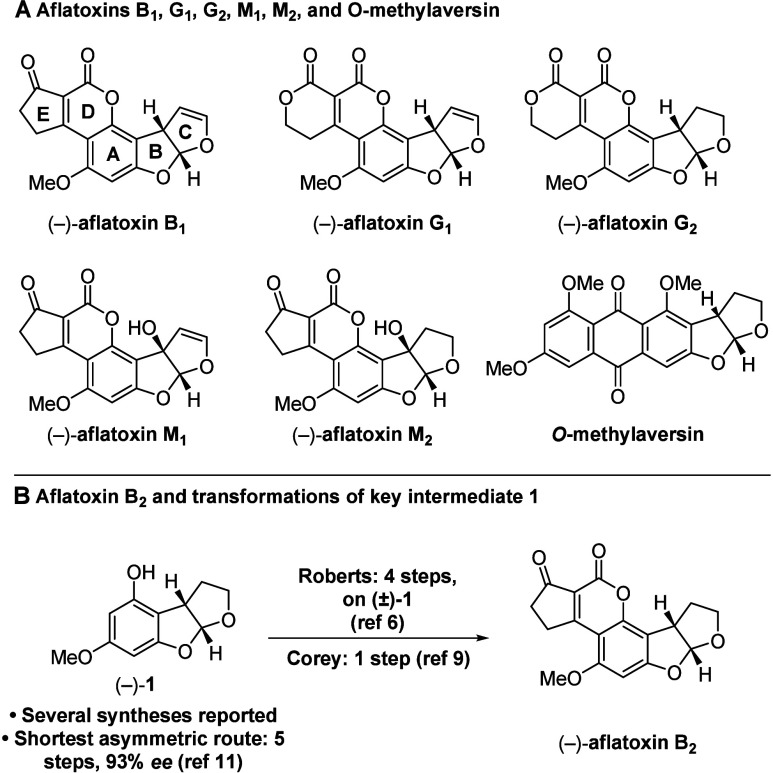
Aflatoxins B_1_, B_2_, G_1_, G_2_, M_1_, and M_2_, *O*-Methylaversin, and Key Intermediate (−)-**1**

Intermediate **1** was first synthesized as a racemate by Roberts and co-workers in 1966,^5^ who subsequently converted this to (±)-aflatoxin B_2_ in four steps (Scheme 1B)^6^ as well as related natural product *O*-methylaversin (Scheme 1A, lower right).^7^ Since then, several groups have reported enantioselective syntheses of intermediate **1**, constituting enantioselective formal syntheses of aflatoxin B_2_, a body of work which has formed a vibrant testing ground for modern enantioselective methodology. Bando and Shishido first achieved this with an enantioselective synthesis of (−)-**1** in 1997 via an asymmetric lipase-catalyzed acetylation (14 steps, 89% ee).^8^ In 2005, Zhou and Corey deployed an enantioselective oxazaborolidinium-catalyzed [3 + 2] cycloaddition (7 steps, 92% ee, refined to 99% by recrystallization); their report also streamlined Roberts’ conversion of **1** to aflatoxin B_2_, reducing it to a single cyclization step (Scheme 1B, below arrow).^9^ While Zhou and Corey’s approach could form the ring system very effectively using an enantioselective [3 + 2] cycloaddition, functional group manipulation was required over six further steps before intermediate (−)-**1** could be obtained due to the required location of the free hydroxyl.^9^ Additional syntheses of **1** using asymmetric catalysis have been reported by Hong (8 steps, 90% ee),^10^ Zu (5 steps, 93% ee),^11^ and Sorensen and Davies (9 steps, 94% ee).^12^ It should be noted that the first asymmetric total synthesis of closely related aflatoxin B_1_, by Trost and Toste, was achieved in 9 total steps, not via intermediate **1**.^13^ These examples serve to illustrate the considerable synthetic efforts that have gone into the enantioselective construction of the tricyclic core of intermediate **1**.

During our studies on the development and application of chiral ligands for Pd catalysis, we became aware of independent reports from the groups of Mazet and Zhang on Pd-catalyzed carboetherification and carboamination of 2,3-dihydrofuran with 2-halophenols and 2-haloanilines (Scheme 2A).^14,15^ These transformations form tetrahydrofurobenzofurans directly if conducted with phenol nucleophiles and furoindolines with anilines: privileged motifs commonly found in natural products. Significant complexity is formed in a single catalytic process, including two stereocenters. However, despite the effectiveness of this methodology, its application to natural product synthesis, most notably the aflatoxins, has remained so far unexploited. We surmised that this may be down to a specific limitation in both groups’ reports: for carboetherification, a substituent on the arene adjacent to the bromide (therefore resulting in a substituent at the 4 position of the tetrahydrobenzofuran product) either results in lower enantioselectivity in Mazet’s case^14a^ or trace reactivity in Zhang’s case (Scheme 2A, inset box, Y = O).^15^ This is a problem for the enantioselective synthesis of intermediate **1**, which contains a hydroxyl at the 4 position, especially considering that this position is neither electronically activated for S_E_Ar nor sterically accessible and therefore not readily amenable to direct transformation of the C–H bond.

**Scheme 2:**
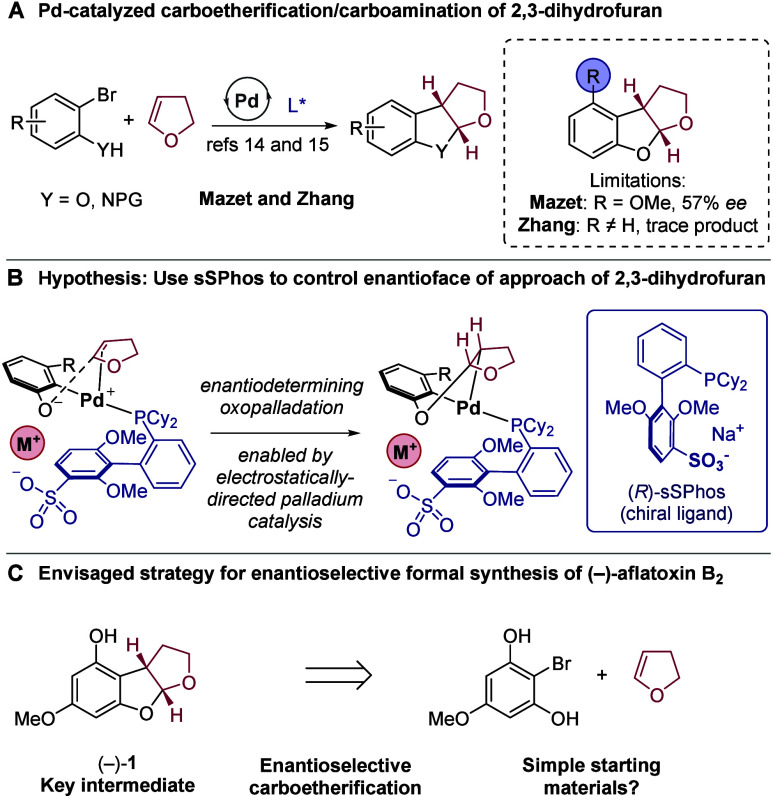
Prior Methodology Development and Our Approach

Our group has an interest in developing enantioselective Pd-catalyzed reactions mediated by chiral, sulfonated ligand sSPhos^16^ and related variants. sSPhos was repurposed by our group, first to control site-selectivity in cross-coupling^17^ and subsequently as a chiral ligand in atroposelective cross-couplings^18^ as well as arylative dearomatization reactions.^19^ In the enantioselective examples, we believe that functionality on the substrate engages in attractive noncovalent interactions with the sulfonate group on sSPhos, creating a highly organized chiral environment conducive to high enantiocontrol.^18,19^ We have had particular success with asymmetric transformations of phenolic substrates, and considering that the carboetherification reaction involves a phenol, we were curious whether sSPhos might constitute a promising chiral ligand for this transformation. A detailed study of the racemic reaction mechanism by Mazet and co-workers suggests that a *syn*-oxopalladation should be enantiodetermining in an asymmetric protocol, involving the addition of 2-bromophenolate across 2,3-dihydrofuran.^14c^ We hypothesized that an attractive electrostatic interaction between the deprotonated phenol, metal cation, and sulfonate group of the ligand might be capable of controlling the enantioface of approach of 2,3-dihydrofuran, by providing organization at the enantiodetermining TS (Scheme 2B).^20^ If this could overcome prior limitations relating to phenol substitution, it could permit a concise enantioselective formal synthesis of (−)-aflatoxin B_2_ (Scheme 2C). Direct formation of the fused ring system from two commercially available components should provide a concise route to key intermediate **1**, if the requisite arene substituents can be incorporated.^4^

We initially evaluated unsubstituted 2-bromophenol, under conditions inspired by those of Mazet and co-workers, with Pd_2_dba_3_ as the palladium source, NaO^*t*^Bu as the base, and toluene as the solvent at 80 °C.^14a^ Use of SPhos as the ligand gave moderate reactivity (Table 1, entry 1), and we were pleased to find that switching to (*R*)-sSPhos delivered the desired tetrahydrofurobenzofuran product **3a** in 97% ee and improved yield (entry 2). Use of KO^*t*^Bu as the base gave a low yield but similarly high ee, suggesting that metal cations of different sizes can participate in the putative electrostatic interaction (entry 3). NaOH gave an almost identical result to NaO^*t*^Bu (entry 4 vs entry 2), and we continued with NaOH for ease of handling. A potassium phosphate base resulted in a lower yield though maintaining excellent enantioselectivity (entry 5), while weaker carbonate bases afforded poor reactivity, suggesting that phenol deprotonation is crucial, as Mazet proposed (entries 6–8).^14c^ As a control experiment to probe the presence of an electrostatic substrate–ligand interaction (see Scheme 2B), we evaluated (*R*)-sSPhos-Np, a sulfonate ester variant of (*R*)-sSPhos, which no longer contains the charged sulfonate group (entry 9). This gave a very poor yield and a −60% ee, delivering the opposite product enantiomer as the major one, despite using the same enantiomer of the ligand scaffold. This divergence in both reactivity and sense of enantioinduction supports our hypothesis that an attractive electrostatic interaction is crucial for the success of sSPhos in the transformation. The intriguing result with sSPhos-Np suggests that a closely related chiral ligand operating primarily on repulsive steric interactions has a different and inferior mode of enantiocontrol.

**Table 1:**
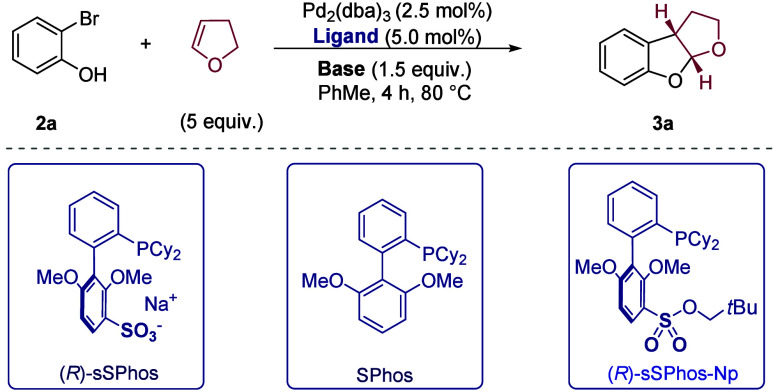
Reaction Optimization

entry	ligand	base	yield (%)^a^	ee (%)^b^
1	SPhos	NaO^*t*^Bu	40	N/A
2	(*R*)-sSPhos	NaO^*t*^Bu	70	97
3	(*R*)-sSPhos	KO^*t*^Bu	10	>99
**4** ^c^	**(*R*)-sSPhos**	**NaOH**	**70 (71)**	**98**
5	(*R*)-sSPhos	K_3_PO_4_	13	>99
6	(*R*)-sSPhos	Na_2_CO_3_	trace	N.D.
7	(*R*)-sSPhos	K_2_CO_3_	trace	N.D.
8	(*R*)-sSPhos	Cs_2_CO_3_	trace	N.D.
9^c^	(*R*)-sSPhos-Np	NaOH	7	–60

a Yields were determined by ^1^H NMR with reference to the internal standard. The value in parentheses refers to the isolated material.

b ee was determined by SFC analysis of the crude reaction mixture, except for entries 3 and 9.

c 16 h reaction time.

With effective conditions in hand, we established that both electron-donating (OMe, **3b**) and -withdrawing (CF_3_, **3c**) substituents could be tolerated on the phenol (Scheme 3). In these examples, the substituent was positioned *para* to the phenol hydroxyl to probe the electronic effect on its acidity/nucleophilicity, but we were encouraged to see that enantioselectivity was excellent in both cases. Pleasingly, a 3-substituted bromophenol also reacted effectively (**3d**). This addressed a key limitation in previous reports and boded well for our future attempts toward aflatoxin B_2_.^14a,15^ The absolute configuration of product **3a** was determined by comparing its optical rotation to those reported by Mazet and Zhang, with absolute configuration of **3b**, **3c**, and **3d** assigned by analogy.^14a,15^ From this information, we could also predict that (*R*)-sSPhos is correctly configured for application in the formal synthesis, which should lead to the natural (−)-enantiomer of aflatoxin B_2_.

**Scheme 3:**
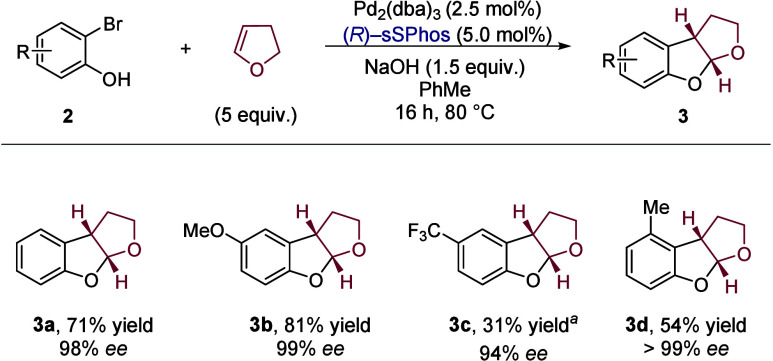
Carboetherification of 2,3-Dihydrofuran with Selected 2-Bromophenols

a The isolated sample contained several trace impurities, which could not be removed by chromatography.

We then turned our attention to the synthesis of key intermediate (−)-**1**. The reaction of 2-bromo-5-methoxybenzene-1,3-diol (**2e**) (Table 2, entry 1), prepared in one step from commercially available 5-methoxybenzene-1,3-diol, would have been particularly advantageous, since it would have directly formed intermediate (−)-**1** in one step, with no post-functionalization required. However, subjection of diol **2e** to the reaction conditions afforded no desired product; we note that the lack of tolerance of a non-participating unprotected phenol is implied by the absence of these from the scopes of Mazet and Zhang’s reports.^14a,15^ A benzyl-protected phenol (**2f**, entry 2), prepared in one step from diol **2e**, also afforded no desired product. With attempts to position oxygen functionality adjacent to bromide being unsuccessful, we reasoned that a chloro substituent in this position might be readily convertible to the desired hydroxyl following the key cyclization. This strategy was initially explored with the desired 5-methoxy substituent in place, but low yields of the cyclized product were obtained, which could not be improved by varying the temperature (entries 3–5).

**Table 2:**
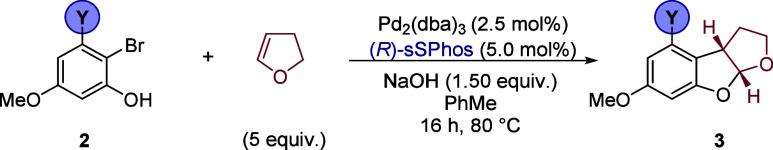
Unsuccessful Aryl Bromides En Route to Key Intermediate (−)-**1**

entry	substrate	Y	temp (°C)	yield (%)^a^
1	**2e**	OH	80	
2	**2f**	OBn	80	
3	**2g**	Cl	80	9
4	**2g**	Cl	110	13
5	**2g**	Cl	50	trace

a Yields were determined by ^1^H NMR with reference to the internal standard.

We eventually found success using commercially available 2-bromo-3-chlorophenol (**2h**), delivering the tricyclic product **3h** in 56% yield and 99% ee (Scheme 4, first step); loadings of catalyst were reduced to 1 mol % Pd and ligand in this scaled-up protocol. Curiously, substrate **2h** differs from **2g** only by its lack of a methoxy, yet they give very different reaction outcomes. We speculate that the methoxy substituent in substrate **2g**, positioned *meta* with respect to the hydroxyl, renders the phenol less nucleophilic (σ_m_ = +0.115 for OMe). The combined effect of both electron-withdrawing substituents (*m*-OMe and *m*-Cl) appears to favor Heck-type reactivity for substrate **2g**, consistent with Mazet’s findings.^14c^ Iridium-catalyzed borylation followed by oxidation installed the required hydroxyl group at the most sterically accessible position of the arene, and this was methylated to afford **5**. Finally, chloride was converted to phenol via a Pd-catalyzed coupling with hydroxide.^21^ This completes the formal synthesis, accessing aflatoxin B_2_ precursor (−)-**1** in 20% overall yield, three chromatographic purifications, and a highly enantioselective key step. The absolute stereochemistry of (−)-**1** was confirmed by comparison of its optical rotation to previous reports.^9,10,12^

**Scheme 4:**
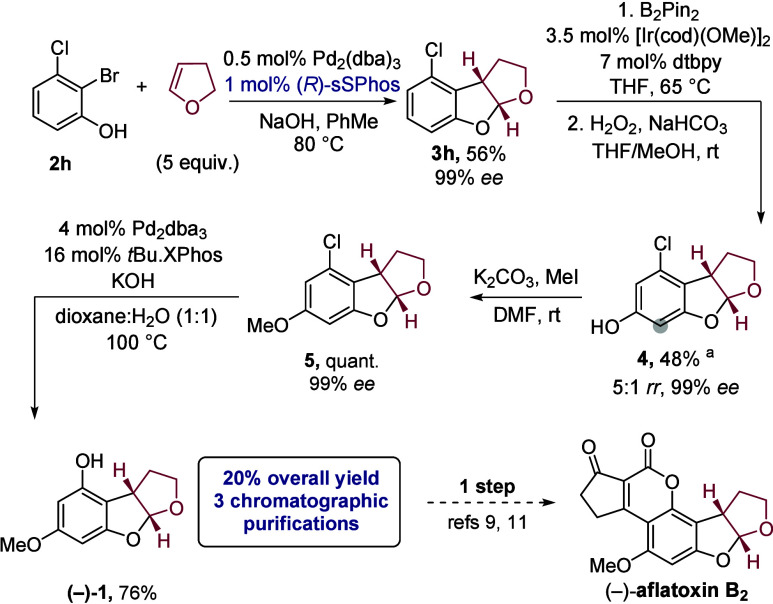
Enantioselective Synthesis of Key Intermediate (−)-**1**

a Corresponds to the isolated sample of the major regioisomer, with rr determined from the crude reaction mixture.

As mentioned earlier, Zhang and co-workers in 2020 developed a highly enantioselective protocol for the analogous carboamination of 2,3-dihydrofuran using their ligand Xiang-Phos.^15^ We questioned whether sSPhos might also constitute an effective chiral ligand for this process to further demonstrate its generality. A brief reevaluation of the conditions revealed a better yield using a binary solvent mixture of PhMe and *tert*-amyl alcohol (TAA), consistent with the findings from Mazet and co-workers in their carboamination studies (Scheme 5A, entries 1 and 2).^14b^ This led to good enantioselectivity of cyclized furoindoline **7a**, suggesting that an effective electrostatic interaction can also occur between deprotonated *N*-tosylaniline **6a** and sSPhos. Both solvent systems were also evaluated at a higher temperature, resulting in a poorer yield (entries 3 and 4). Investigation of a more electron-withdrawing Tf-protecting group resulted in worse enantioselectivity (see the Supporting Information). A brief investigation also showed high enantioselectivities for furoindolines **7b** and **7c**, with both substrates displaying extremes of electronics *para* to the aniline nitrogen, suggesting that a broad variety of electronic character on the aniline should be tolerated (Scheme 5B). The absolute configuration of the products was consistent with the carboetherification.^15^

**Scheme 5:**
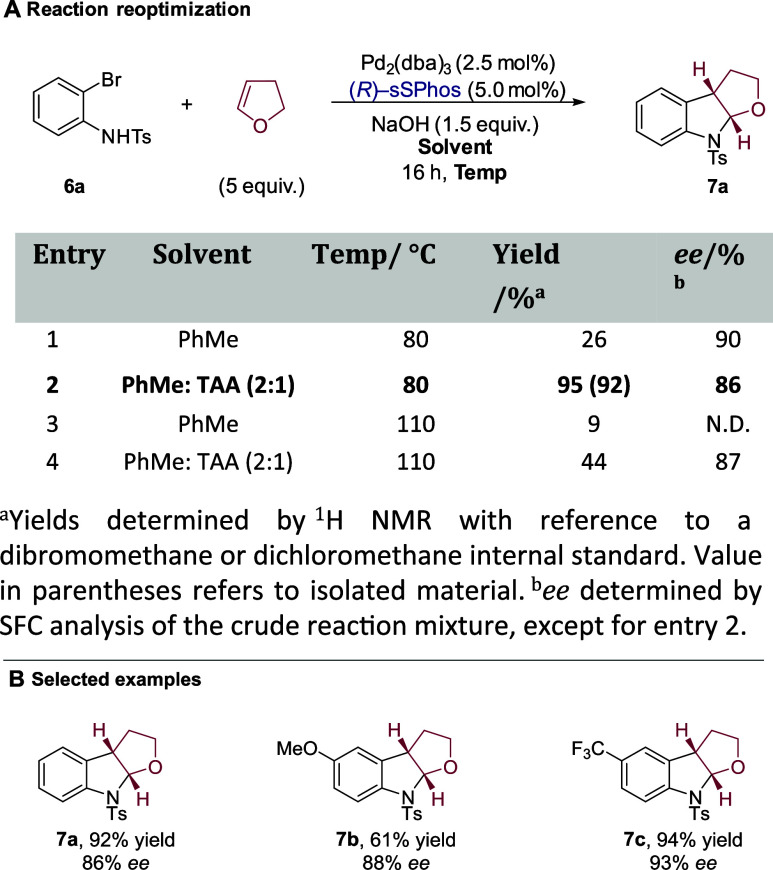
Carboamination of 2,3-Dihydrofuran with 2-Bromo-*N*-tosylanilines

In conclusion, we describe the application of the chiral ligand sSPhos to the Pd-catalyzed reaction of 2-bromophenols with 2,3-dihydrofuran to form tetrahydrobenzofurans, enabled by electrostatically directed palladium catalysis. This has allowed us to overcome a limitation in the previous literature, permitting two representative phenols with a substituent adjacent to bromide to be engaged in the reaction with excellent enantioselectivities. This has enabled one of the most concise and enantioselective formal syntheses of aflatoxin-B_2_ reported to date with the key intermediate (−)-**1** accessed in 20% overall yield over five steps, requiring only three chromatographic purifications, and in 99% ee. The enantioselective synthesis of the aflatoxins has challenged synthetic chemists for decades; we hope that our realization of a palladium-catalyzed approach for the key complexity-generating step may inspire other synthetic strategies toward related polycyclic molecules of biological relevance.

## Supplementary Material



## Data Availability

The data underlying this study are available in the published article and its Supporting Information.

## Notes

Full experimental details, characterization data for compounds, copies of NMR spectra, and SFC traces (PDF)
