# Supplementary material for: Enantioselective Formal Synthesis of (−)-Aflatoxin B2 Enabled by Pd-Catalyzed Carboetherification of 2,3-Dihydrofuran
Source: Org Lett. 2025 Jul 18;27(30):8344–8. doi: 10.1021/acs.orglett.5c02618 (PMC12322965; doi:10.1021/acs.orglett.5c02618)

Supporting Information

Enantioselective Formal Synthesis of (−)-Aflatoxin B_2 Enabled by Pd-Catalyzed Carboetherification of 2,3-Dihydrofuran

Max Kadarauch and Robert J. Phipps*

Yusuf Hamied Department of Chemistry, University of Cambridge, Lensfield Road, Cambridge, CB2 1EW, United Kingdom.

Table of Contents

General Experimental 3

Investigation of Tf-Protected Aniline 5

Synthesis of Substrates in Table 2 5

Characterization Data for Products in Scheme 3 7

General Procedure A: Palladium/(*R*)-sSPhos-Catalyzed Carboetherification of 2,3-Dihydrofuran with 2-Bromophenols 7

Determination of Absolute Stereochemistry of Carboetherification Products with 2-Bromophenols 7

Characterization Data for Compounds in Scheme 4 10

Synthesis of (3a*S*,8a*R*)-4-chloro-2,3,3a,8a-tetrahydrofuro[2,3-*b*]benzofuran-7-ol (S5) and (3a*S*,8a*R*)-4-chloro-2,3,3a,8a-tetrahydrofuro[2,3-*b*]benzofuran-6-ol (4) 12

Characterization Data for Products in Scheme 5 16

General Procedure B: Palladium/(*R*)-sSPhos-Catalyzed Carboamination of 2,3-Dihydrofuran with 2-Bromoanilines 16

Determination of Absolute Stereochemistry for Carboamination Products with 2-Bromoanilines 16

References 19

Chiral SFC Traces 21

NMR Spectra 31

General Experimental

<u>Solvents</u>: THF, MeOH, CH_2Cl_2, and PhMe were purified by distillation on site under inert atmosphere via the following processes. THF was pre-dried over sodium wire and then distilled from calcium hydride and lithium aluminum hydride. MeOH, CH_2Cl_2, and PhMe were distilled from calcium hydride. *tert*-Amyl alcohol (TAA) was sparged with nitrogen before use.

<u>Reagents</u>: All reagents were used as supplied from commercial sources with no further purification. NaOH was finely ground using a pestle and mortar. (*R*)-sSPhos was prepared by resolution of (*rac*)-sSPhos by preparative SFC, according to our previous publication.[1] Alternatively, (*R*)-sSPhos can also be obtained via recrystallization of diastereomeric quinidine salts, according to our previous publication.[1] (*R*)-sSPhos-Np was prepared according to our previous publication.[2] Pd_2dba_3 was purchased from commercial sources but repurified prior to use, according the method described by Zalesskiy and Ananikov.[3] *N*-(2-bromophenyl)-4-methylbenzenesulfonamide (**6a**),[4] *N*-(2-bromo-4-methoxyphenyl)-4-methylbenzenesulfonamide (**6b**),[5] and *N*-(2-bromo-4-(trifluoromethyl)phenyl)-4-methylbenzenesulfonamide (**6b**)[6] were prepared according to the literature.

<u>Chromatography</u>: Flash column chromatography was performed using 60A (40-63 μM) from Fluorochem. Crude compounds were dried directly onto silica gel and then loaded onto a pre-equilibrated silica column eluting with the solvent system specified under a positive pressure of air. Thin layer chromatography (TLC) was performed using 0.25 mm E. Merck silica plates (60F-254). The plates were visualized using ultraviolet radiation (254 nm) or a potassium permanganate stain where appropriate.

<u>Reactions</u>: Optimization experiments and scope examples were carried out on a 0.2 mmol scale in 4 mL 13 mm crimp ring vials. The 2 mmol scale up experiment (synthesis of **3h**) was carried out in a 10 mL Biotage microwave vial.

<u>Data collection</u>: ^{1}H NMR spectra were recorded on 400 MHz QNP cryoprobe, 400 MHz AVIII HD smart probe, 400 MHz Advance III HD, 400 MHz Neo Prodigy, 500 MHz DCH Cryoprobe, and 700 MHz TXO Cryoprobe spectrometers. The chemical shifts, reported in parts per million (δ ppm) were recorded relative to the residual undeuterated solvent ($CDCl_3$: 7.26 ppm, DMSO-d_6: 2.50 ppm, benzene-d_6: 7.16). Multiplicity is recorded as follows: s = singlet, d = doublet, t = triplet, q = quartet, pent = pentet, sext = sextet, m = multiplet, br = broad with associated combinations. Coupling constant (*J*, Hz) and peak integrations (nH) are also reported. ^{13}C NMR

spectra were recorded on the same machines with complete proton decoupling. The chemical shifts, reported in parts per million (δ ppm) were recorded relative to the residual undeuterated solvent ($CDCl_3$: 77.16 ppm, DMSO-d_6: 39.52, benzene-d_6: 128.06). ^{19}F NMR spectra were recorded on 400 MHz QNP cryoprobe, 400 MHz AVIII HD smart probe and 400 MHz Advance III HD spectrometers.

<u>Chiral SFC Analysis:</u> Performed on a Waters ACQUITY UPC2 System with DAICEL CHIRALPAK IA, IE, IJ, or IK columns (4.6 x 250 mm, 3.0 μm) in a mixed solvent system of supercritical CO_2 and MeOH. A system backpressure of 138 bar was used in all cases.

<u>High Resolution Mass Spectrometry (HRMS):</u> Recorded on a Waters Vion IMS QTOF or AGILENT 6230 LC/TOF at the Department of Chemistry at the University of Cambridge. The ionization method is noted as either positive or negative electrospray ionisation (+/–ESI). Measured values are reported to 4 decimal places and are within ± 5 ppm of the calculated value. The calculated values are based on the most abundant isotope unless otherwise stated in the chemical formula.

<u>Optical Rotations:</u> measured in $CHCl_3$ or CH_2Cl_2 on a Perkin Elmer 343 Polarimeter using a sodium lamp (λ = 589 nm, D-line). $[\alpha]_D$ values are reported at 25.0 °C in degrees mL g^{-1} dm^{-1} with concentration (c) in cg mL^{-1}.

<u>Racemic Reactions:</u> Reactions to obtain racemic SFC traces were run with (*rac*)-sSPhos.

Investigation of Tf-Protected Aniline

A Tf-protected aniline formed the desired product in poor enantioselectivity (**S2**, 48% *ee*) when investigated under similar reaction conditions to those used in Scheme 5.

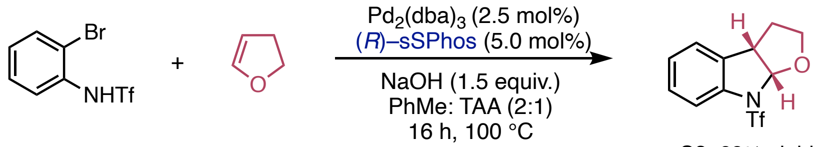

S1 (5.00 equiv.) **S2**, 68% yield 48% *ee*

Synthesis of Substrates in Table 2

Synthesis of 3-(benzyloxy)-2-bromo-5-methoxyphenol (2f)

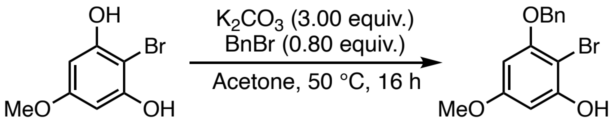

2e **2f, 25% yield**

2-Bromo-5-methoxybenzene-1,3-diol (**2e**) was prepared according to the literature.[7]

3-(benzyloxy)-2-Bromo-5-methoxyphenol (2f)

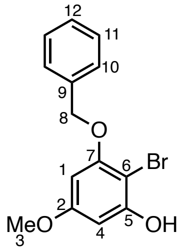

To a solution of 2-bromo-5-methoxybenzene-1,3-diol (**2e**) (175 mg, 0.800 mmol, 1.00 equiv.) in acetone (2 mL) was added K_2CO_3 (332 mg, 2.40 mmol, 3.00 equiv.). The stirred mixture was heated to 50 °C in a preheated aluminum heating block, and benzyl bromide (109 mg, 75.7 µL, 0.640 mmol, 0.800 equiv.) was added slowly. The reaction was stirred at 50 °C for 16 h. Upon completion, the reaction was cooled to room temperature and concentrated under a stream of nitrogen. EtOAc and sat. NH_4Cl (aq.) were added, and the layers were separated. The aqueous phase was further extracted with EtOAc (2×). The combined organic extracts were dried over Mg_2SO_4, filtered, concentrated, and purified by flash column chromatography (0-20% EtOAc in 40-60 °C petroleum ether) to afford the title compound as a colorless oil (62.7 mg, 0.203 mmol, 25% yield).

^{1}H NMR (400 MHz, $CDCl_3$) δ 7.49 – 7.44 (m, 2H, H_{10}), 7.43 – 7.37 (m, 2H, H_{11}), 7.36 – 7.30 (m, 1H, H_{12}), 6.28 (d, J = 2.6 Hz, 1H, H_4), 6.17 (d, J = 2.6 Hz, 1H, H_1), 5.68 (s, 1H, H_O), 5.11 (s, 2H, H_8), 3.75 (s, 3H, H_3).

^{13}C NMR (101 MHz, $CDCl_3$) δ 160.7, 156.0, 154.1, 136.5, 128.7, 128.1, 127.2, 94.1, 93.7, 91.9, 70.9, 55.7.

HRMS m/z: $[M+H]^+$ calculated for $[C_{14}H_{14}BrO_3]^+$ 309.0121, found 309.0122. Δ = +0.3 ppm.

Synthesis of 2-bromo-3-chloro-5-methoxyphenol (2g)

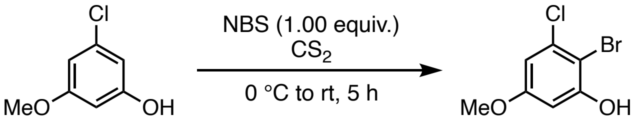

2-Bromo-3-chloro-5-methoxyphenol (2g)

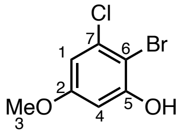

A solution of 3-chloro-5-methoxyphenol (1.30 g, 8.20 mmol, 1.00 equiv.) in CS_2 (20 mL) was cooled to 0 °C. *N*-Bromosuccinimide (1.46 g, 8.20 mmol, 1.00 equiv.) was added in portions, and the reaction was slowly warmed to room temperature over 2 h, and stirred at room temperature for a further 3 h. The reaction mixture was concentrated under a stream of air, and purified by flash column chromatography (0-50% $CHCl_3$ in 40-60 °C petroleum ether) to afford the title compound as a colorless solid (319 mg, 1.34 mmol, 16% yield).

^{1}H NMR (500 MHz, $CDCl_3$) δ 6.66 (d, J = 2.8 Hz, 1H, H_1), 6.51 (d, J = 2.8 Hz, 1H, H_4), 5.67 – 5.64 (m, 1H, H_O), 3.77 (s, 3H, H_3).

^{13}C NMR (126 MHz, $CDCl_3$) δ 160.1, 154.3, 134.7, 109.0, 102.4, 100.4, 55.9.

HRMS m/z: $[M–H]^–$ calculated for $[C_7H_5BrClO_2]^–$ 234.9167, found 234.9163. Δ = –1.7 ppm.

Evidence for formation of depicted structure over other isomers:

Strong HMBC correlation between C_2 (160.1) and both aromatic ^{1}H signals (H_1 and H_4).

Strong HMBC correlation between C_5 (154.3) and one aromatic ^1H signal (H_4), but not the other (H_1).

Strong HMBC correlation between C_7 (134.7) and one aromatic ^1H signal (H_1) but not the other (H_4).

N.B. Upon subjection of the title compound to the reaction conditions described in Table 2 (entries 3 and 4), a product was observed in the ^1H spectrum of the crude reaction which matched that of intermediate **5**, formed via methylation of intermediate **4** (see Scheme 4). This was taken as further evidence of the title compound **2g** having the structure depicted, with the Br occupying the position between the OH and the Cl.

Characterization Data for Products in Scheme 3

General Procedure A: Palladium/(*R*)-sSPhos-Catalyzed Carboetherification of 2,3-Dihydrofuran with 2-Bromophenols

A vial containing a magnetic stirrer bar was sequentially charged with the relevant 2-bromophenol (0.200 mmol, 1.00 equiv.), (*R*)-sSPhos (5.1 mg, 0.010 mmol, 5.0 mol%), NaOH (12.0 mg, 0.300 mmol, 1.50 equiv.), and Pd_2dba_3 (4.6 mg, 0.0050 mmol, 2.5 mol%). The vial was sealed, and evacuated and backfilled with nitrogen (4×). 2,3-Dihydrofuran (70.1 mg. 75.6 µL, 1.00 mmol, 5.00 equiv.) was added under nitrogen. PhMe (1.00 mL) was added under nitrogen. The reaction was stirred at 80 °C and 900 rpm in a preheated aluminum heating block for 16 h. Upon completion, the vial was opened, and the reaction mixture was filtered through celite, eluting with Et_2O. The filtrate was concentrated under a stream of air. The crude product was purified by flash column chromatography.

Determination of Absolute Stereochemistry of Carboetherification Products with 2-Bromophenols

The optical rotation of **3a** was compared to that reported by Mazet and co-workers,[8] as well as Zhang and co-workers,[9] enabling absolute stereochemistry of **3a** to be assigned. Stereochemistry of the remaining products in Scheme 3 (**3b**, **3c**, **3d**) are assigned by analogy.

(3a*S*,8a*R*)-2,3,3a,8a-Tetrahydrofuro[2,3-*b*]benzofuran (3a)

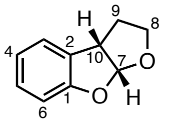

Prepared according to General Procedure A with 2-bromophenol (34.6 mg, 0.200 mmol) as the starting material. Purification by flash column chromatography (0-5% Et_2O in 40-60 °C petroleum ether) afforded the title compound as a colorless oil (23.0 mg, 0.142 mmol, 71% yield, 98% *ee*).

[N.B. The product was found to be volatile when left under high vacuum for prolonged periods. The product was therefore concentrated on a rotary evaporator at 80 mbar and 40 °C.]

^{1}H NMR (500 MHz, $CDCl_3$) δ 7.19 (dd, *J* = 7.5, 1.3 Hz, 1H, H_3), 7.15 (app tdd, *J* = 8.0, 1.3, 0.8 Hz, 1H, H_5), 6.91 (app td, *J* = 7.4, 1.0 Hz, 1H, H_4), 6.81 (d, *J* = 8.0 Hz, 1H, H_6), 6.31 (d, *J* = 5.7 Hz, 1H, H_7), 4.11 – 4.04 (m, 1H, H_8), 4.01 (dd, *J* = 8.6, 5.7 Hz, 1H, H_{10}), 3.62 (ddd, *J* = 12.1, 8.6, 4.9 Hz, 1H, $H_{8'}$), 2.30 (app tdd, *J* = 12.1, 8.6, 7.6 Hz, 1H, H_9), 2.07 (dd, *J* = 12.1, 4.9 Hz, 1H, $H_{9'}$).

^{13}C NMR (126 MHz, $CDCl_3$) δ 159.6, 128.8, 127.8, 124.8, 121.3, 111.0, 109.3, 67.4, 46.7, 33.7.

Chiral SFC Analysis: CHIRALPAK IK (CO_2:MeOH, 99:01, 2.5 mL min^{-1}, 40 °C, 220 nm) indicated 98% *ee*, t_R = 4.80 (major), 5.14 (minor) minutes.

$[\alpha]_D^{25}$ = –92.6° (c 1.15, CH_2Cl_2). Literature values: **$[\alpha]_D^{23}$** = –142.2° (c 0.85, CH_2Cl_2) for 92% *ee*;[8] **$[\alpha]_D^{20}$** = –94.038 (c = 0.5, CH_2Cl_2) for 96% *ee*.[9]

^{1}H and ^{13}C NMR data consistent with the literature.[9]

(3a*S*,8a*R*)-5-Methoxy-2,3,3a,8a-tetrahydrofuro[2,3-*b*]benzofuran (3b)

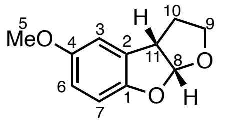

Prepared according to General Procedure A with 2-bromo-4-methoxyphenol (40.6 mg, 0.200 mmol) as the starting material. Purification by flash column chromatography (0-10% Et_2O in 40-60 °C petroleum ether) afforded the title compound as a yellow oil (31.3 mg, 0.163 mmol, 81% yield, 99% *ee*).

^{1}H NMR (700 MHz, $CDCl_3$) δ 6.76 (d, *J* = 2.5 Hz, 1H, H_3), 6.71 (d, *J* = 8.6 Hz, 1H, H_7), 6.68 (dd, *J* = 8.6, 2.5 Hz, 1H, H_6), 6.28 (d, *J* = 5.6 Hz, 1H, H_8), 4.06 (ddd, *J* = 8.6, 7.6, 0.8 Hz, 1H, H_9), 3.98 (dd, *J* = 8.6, 5.6 Hz, 1H, H_{11}), 3.76 (s, 3H, H_5), 3.62 (ddd, *J* = 12.1, 8.6, 4.8 Hz, 1H, $H_{9'}$), 2.28 (app tdd, *J* = 12.1, 8.6, 7.6 Hz, 1H, H_{10}), 2.06 (ddd, *J* = 12.1, 4.8, 0.8 Hz, 1H, $H_{10'}$).

^{13}C NMR (176 MHz, $CDCl_3$) δ 154.7, 153.7, 128.6, 113.7, 111.3, 110.9, 109.3, 67.3, 56.1, 47.2, 33.6.

Chiral SFC Analysis: CHIRALPAK IJ (CO_2:MeOH, 95:05, 2.5 mL min^{-1}, 40 °C, 220 nm) indicated 99% *ee*, t_R = 3.22 (major), 3.61 (minor) minutes.

$[\alpha]_D^{25}$ = –122° (c 1.57, $CHCl_3$).

Data consistent with the literature.[8]

(3a*S*,8a*R*)-5-(trifluoromethyl)-2,3,3a,8a-Tetrahydrofuro[2,3-*b*]benzofuran (3c)

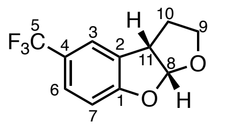

Prepared according to General Procedure A with 2-bromo-4-(trifluoromethyl)phenol (48.2 mg, 0.200 mmol) as the starting material. Purification by flash column chromatography (0-5% Et_2O in 40-60 °C petroleum ether) afforded the title compound, alongside several unidentified trace impurities, as a yellow oil. (14.3 mg, 0.0621 mmol [not accounting for impurities], 31% yield [not accounting for impurities], 94% *ee*).

[N.B. The product was found to be volatile when left under high vacuum for prolonged periods. The product was therefore concentrated on a rotary evaporator at 80 mbar and 40 °C.]

^{1}H NMR (500 MHz, C_6D_6) δ 7.15 – 7.12 (m, 1H, H_6), 7.04 – 7.01 (m, 1H, H_3), 6.52 (dd, J = 8.4, 0.9 Hz, 1H, H_7), 5.97 (d, J = 5.7 Hz, 1H, H_8), 3.53 (ddd, J = 8.7, 7.7, 0.8 Hz, 1H, H_9), 3.14 (ddd, J = 12.1, 8.7, 4.9 Hz, 1H, $H_{9'}$), 3.01 (dd, J = 8.8, 5.7 Hz, 1H, H_{11}), 1.51 – 1.40 (m, 1H, H_{10}), 1.15 (ddd, J = 12.4, 4.9, 0.8 Hz, 1H, $H_{10'}$).

^{13}C NMR (126 MHz, C_6D_6) δ 162.7 (q, J = 1.3 Hz), 129.3, 126.9 (q, J = 3.9 Hz), 125.4 (d, J = 271.1 Hz), 123.4 (q, J = 32.2 Hz), 122.4 (q, J = 3.7 Hz), 112.3, 109.3, 66.9, 46.0, 33.3.

^{19}F NMR (376 MHz, $CDCl_3$) δ –61.1.

Chiral SFC Analysis: CHIRALPAK IJ (CO_2:MeOH, 99:01, 2.5 mL min^{-1}, 40 °C, 230 nm) indicated 94% *ee*, t_R = 2.17 (major), 2.65 (minor) minutes.

$[\alpha]_D^{25}$ = –62.0° (c 0.95, $CHCl_3$).

^{1}H and ^{13}C data consistent with the literature.[10]

(3a*S*,8a*R*)-4-Methyl-2,3,3a,8a-tetrahydrofuro[2,3-*b*]benzofuran (3d)

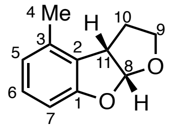

Prepared according to General Procedure A with 2-bromo-3-methylphenol (37.4 mg, 0.200 mmol) as the starting material. Purification by flash column chromatography (0-5% Et_2O in 40-60 °C petroleum ether) afforded the title compound as a colorless solid (19.0 mg, 0.108 mmol, 54% yield, >99% *ee*).

^{1}H NMR (400 MHz, $CDCl_3$) δ 7.05 (app t, J = 7.8 Hz, 1H, H_6), 6.71 (d, J = 7.8 Hz, 1H, H_5), 6.64 (d, J = 7.8 Hz, 1H, H_7), 6.34 (d, J = 5.8 Hz, 1H, H_8), 4.09 (ddd, J = 8.7, 7.7, 1.0 Hz, 1H, H_9), 3.96 (dd, J = 9.0, 5.8 Hz, 1H, H_{11}), 3.67 (ddd, J = 12.0, 8.7, 5.2 Hz, 1H, $H_{9'}$), 2.32 (s, 3H, H_4), 2.29 – 2.20 (m, 1H, H_{10}), 2.11 – 2.01 (m, 1H, $H_{10'}$).

^{13}C NMR (101 MHz, $CDCl_3$) δ 159.4, 134.8, 128.7, 126.4, 122.3, 111.1, 106.6, 67.1, 45.9, 32.0, 18.5.

HRMS m/z: $[M+H]^+$ calculated for $[C_{11}H_{13}O_2]^+$ 177.0910, found 177.0910. Δ = 0.0 ppm.

Chiral SFC Analysis: CHIRALPAK IK (CO_2:MeOH, 99:01, 2.5 mL min^{-1}, 40 °C, 220 nm) indicated >99% *ee*, t_R = 6.03 (major), 6.63 (minor) minutes.

$[\alpha]_D^{25}$ = –190.4° (c 0.95, $CHCl_3$).

Characterization Data for Compounds in Scheme 4

(3a*S*,8a*R*)-4-Chloro-2,3,3a,8a-tetrahydrofuro[2,3-*b*]benzofuran (3h)

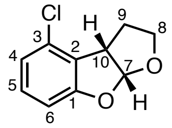

A vial containing a magnetic stirrer bar was sequentially charged with 2-bromo-3-chlorophenol (415 mg, 2.00 mmol, 1.00 equiv.), (*R*)-sSPhos (10.2 mg, 0.0200 mmol, 1.00 mol%), NaOH (120 mg, 3.00 mmol, 1.50 equiv.), and Pd_2dba_3 (9.2 mg, 0.010 mmol, 0.50 mol%). The vial was sealed, and evacuated and backfilled with nitrogen (4×). 2,3-Dihydrofuran (701 mg, 0.756 mL, 10.0 mmol, 5.00 equiv.) was added under nitrogen. PhMe (10.0 mL) was added under nitrogen. The reaction was stirred at 80 °C and 900 rpm in a preheated aluminum heating block for 3 days.

Upon completion, the reaction mixture was filtered through celite, eluting with Et_2O. The filtrate was concentrated under a stream of air. The crude product was purified by flash column chromatography (0-2% EtOAc in 40-60 °C petroleum ether) to afford the title compound along with a 14% mol/mol impurity (total sample mass: 264 mg), suspected to be a Heck product arising from migratory insertion of the palladium (II) intermediate into 2,3-dihydrofuran and subsequent β-hydride elimination. This impurity was removed to < 5% according to the following procedure.

To 124 mg of the columned sample (124 mg/264 mg = 47.0% of the total) was added NaOH (aq.) (2 M, 1 mL) and $CDCl_3$ (1 mL), and the layers separated. The aqueous layer was further extracted with $CDCl_3$ (2×1 mL). The combined organic extracts were dried over Mg_2SO_4, filtered, and concentrated under reduced pressure to afford the title compound as a colorless oil (104 mg, 0.529 mmol, 56% corrected yield, 99% *ee*).

^{1}H NMR (400 MHz, $CDCl_3$) δ 7.08 (app t, J = 8.0 Hz, 1H, H_5), 6.87 (d, J = 8.0 Hz, 1H, H_4), 6.70 (d, J = 8.0 Hz, 1H, H_6), 6.35 (d, J = 5.7 Hz, 1H, H_7), 4.11 (ddd, J = 8.7, 6.9, 1.5 Hz, 1H, H_8), 4.05 (ddd, J = 7.8, 5.7, 1.9 Hz, 1H, H_{10}), 3.65 (ddd, J = 11.8, 8.7, 5.8 Hz, 1H, $H_{8'}$), 2.34 – 2.18 (m, 2H, H_9).

^{13}C NMR (101 MHz, $CDCl_3$) δ 160.5, 130.8, 130.0, 126.0, 121.4, 111.2, 107.7, 67.4, 46.7, 31.5.

HRMS m/z: $[M+H]^+$ calculated for $[C_{10}H_{10}ClO_2]^+$ 197.0364, found 197.0367. Δ = +1.5 ppm.

Chiral SFC Analysis: CHIRALPAK IJ (CO_2:MeOH, 99:01, 2.5 mL min^{-1}, 40 °C, 220 nm) indicated 99% *ee*, t_R = 3.52 (major), 3.72 (minor) minutes.

$[\alpha]_D^{25}$ = –252° (c 0.79, $CHCl_3$).

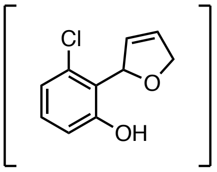

Suspected impurity
Same MW as 3h

[N.B. The remaining 140 mg of the columned sample (i.e. still containing the 14% mol/mol impurity) was subjected to the unoptimized borylation conditions described below, for the preparation of an authentic sample of the minor regioisomer (**S5**) in the borylation/oxidation step.]

Synthesis of (3a*S*,8a*R*)-4-chloro-2,3,3a,8a-tetrahydrofuro[2,3-*b*]benzofuran-7-ol (S5) and (3a*S*,8a*R*)-4-chloro-2,3,3a,8a-tetrahydrofuro[2,3-*b*]benzofuran-6-ol (4)

[N.B. Relating to the synthesis of **S5** and **4**: We found that performing the reaction under slightly different conditions (different ligand, higher catalyst loadings, purer starting material) favored the formation of **4** as the major product rather than **S5** (5:1 selectivity vs 1:1.4 selectivity). Both procedures are described below.]

(3a*S*,8a*R*)-4-Chloro-2,3,3a,8a-tetrahydrofuro[2,3-*b*]benzofuran-7-ol (S5)

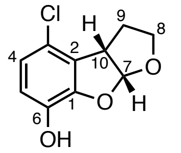

[N.B. Unoptimized procedure for preparation of an authentic sample of minor regioisomer **S5**.]

An oven-dried vial and stirrer bar were cooled under vacuum, and charged with the sample of (3a*S*,8a*R*)-4-chloro-2,3,3a,8a-tetrahydrofuro[2,3-*b*]benzofuran containing a 14% mol/mol impurity (see earlier) (**3h**) (140 mg, 0.612 mmol [accounting for the impurity], 1.00 equiv.), 3,4,7,8-tetramethyl-1,10-phenanthroline (5.7 mg, 0.024 mmol, 4.0 mol%), B_2pin_2 (233 mg, 0.918 mmol, 1.50 equiv.), and $[Ir(COD)(OMe)]_2$ (8.0 mg, 0.012 mmol, 2.0 mol%). The vial was sealed, and evacuated and backfilled with nitrogen (4×). THF (3 mL) was added under nitrogen, and the reaction was stirred at 65 °C in a preheated aluminum heating block for 16 h. Upon completion, MeOH (3 mL) was added. $NaHCO_3$ (252 mg, 3.00 mmol) and H_2O_2 (100 vol, 0.72 mL, 7.1 mmol) were added, and the mixture was stirred at room temperature for 1 h. The solvent was removed under a stream of air. Water (2 mL) and CH_2Cl_2 (2 mL) were added, and the layers separated. The aqueous phase was further extracted with CH_2Cl_2 (2×). The combined organic extracts were dried over Mg_2SO_4 and filtered. ^{1}H NMR of the crude reaction mixture in $CDCl_3$ showed a 1:1.4 mixture of regioisomers (**4**:**S5**). The crude mixture was purified by flash column chromatography (0-10% EtOAc in 40-60 °C petroleum ether) to sequentially afford (3a*S*,8a*R*)-4-chloro-2,3,3a,8a-tetrahydrofuro[2,3-*b*]benzofuran-6-ol (**4**) as a colorless solid (31.5 mg, 0.148 mmol, 24% yield) and the title compound (**S5**) as a colorless solid. Due to the challenging separation of regioisomers, the isolated sample of **S5** contained a 7% mol/mol impurity of regioisomer **4** (46.5 mg, 0.203 mmol [accounting for impurity], 33% yield).

^{1}H NMR (500 MHz, $CDCl_3$) δ 6.77 (d, J = 8.6 Hz, 1H, H_4), 6.73 (dd, J = 8.6, 0.6 Hz, 1H, H_5), 6.41 (d, J = 5.7 Hz, 1H, H_7), 4.87 (s, 1H, H_O), 4.18 – 4.06 (m, 2H, 1×H_8 and H_{10}), 3.69 (ddd, J = 11.9, 8.8, 5.3 Hz, 1H, $H_{8'}$), 2.38 – 2.19 (m, 2H, 2×H_9).

^{13}C NMR (176 MHz, $CDCl_3$) δ 147.0, 138.4, 126.1, 121.9, 121.5, 116.8, 112.1, 67.8, 47.8, 31.3.

Evidence for formation of depicted structure over C_4-oxidized product (N.B. C_4 refers to compound numbering shown above, not the IUPAC fused-ring heterocyclic nomenclature):

Strong HMBC correlation between C_3 (121.5) and both aromatic ^{1}H signals (H_4 and H_5).

Strong HMBC correlation between C_1 (147.0) and one aromatic ^{1}H signal (H_5), but not the other (H_4).

HRMS m/z: $[M–H]^-$ calculated for $[C_{10}H_8ClO_3]^-$ 211.0167, found 211.0175. Δ = +3.8 ppm.

$[\alpha]_D^{25}$ = –271° (c 0.35, $CHCl_3$).

(3a*S*,8a*R*)-4-Chloro-2,3,3a,8a-tetrahydrofuro[2,3-*b*]benzofuran-6-ol (4)

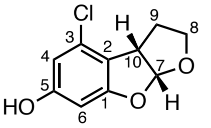

An oven-dried vial and stirrer bar were cooled under vacuum, and charged with (3a*S*,8a*R*)-4-chloro-2,3,3a,8a-tetrahydrofuro[2,3-*b*]benzofuran (**3h**) (12.6 mg, 0.0641 mmol, 1.00 equiv.), 4,4′-di-*tert*-butyl-2,2′-bipyridine (1.2 mg, 0.0045 mmol, 7.0 mol%), B_2pin_2 (24.4 mg, 0.0962 mmol, 1.50 equiv.), and $[Ir(COD)(OMe)]_2$ (1.5 mg, 0.0022 mmol, 3.5 mol%). The vial was sealed, and evacuated and backfilled with nitrogen (4×). THF (0.3 mL) was added under nitrogen, and the reaction was stirred and heated to 65 °C in a preheated aluminum heating block for 16 h. Upon completion, the THF was removed under a stream of air, and the residue redissolved in MeOH: THF (0.2 mL and 0.2 mL). $NaHCO_3$ (21.0 mg, 0.250 mmol) and H_2O_2 (100 vol, 0.06 mL, 0.6 mmol) were added, and the mixture was stirred at room temperature for 1 h. The solvent was removed under a stream of air. Water (0.5 mL) and $CDCl_3$ (0.5 mL) were added, and the layers were separated. The aqueous phase was further extracted with $CDCl_3$ (2×). The combined organic extracts were dried over Mg_2SO_4 and filtered. ^{1}H NMR of the crude reaction mixture in $CDCl_3$ showed a 5:1 mixture of regioisomers (**4**:**S5**). The crude mixture was purified by flash column chromatography (0-7.5% EtOAc in 40-60 °C petroleum ether) to afford the title compound (single regioisomer) as a colorless solid (6.5 mg, 0.031 mmol, 48% yield, 99% *ee*).

[N.B. The preparation of an authentic sample of the minor regioisomer (**S5**) is described earlier.]

^{1}H NMR (500 MHz, $CDCl_3$) δ 6.41 (d, *J* = 2.1 Hz, 1H, H_4), 6.34 (d, *J* = 5.7 Hz, 1H, H_7), 6.25 (d, *J* = 2.1 Hz, 1H, H_6), 5.65 (s, 1H, H_O), 4.11 (ddd, *J* = 8.7, 7.3, 1.2 Hz, 1H, H_8), 4.02 – 3.96 (m, 1H, H_{10}), 3.67 (ddd, *J* = 12.0, 8.7, 5.3 Hz, 1H, $H_{8'}$), 2.32 – 2.13 (m, 2H, H_9).

^{13}C NMR (126 MHz, $CDCl_3$) δ 161.1, 157.4, 130.9, 118.0, 111.9, 108.8, 96.2, 67.6, 46.2, 31.6.

HRMS m/z: $[M+H]^+$ calculated for $[C_{10}H_{10}ClO_3]^+$ 213.0313, found 213.0308. Δ = –2.3 ppm.

Chiral SFC Analysis: CHIRALPAK IE (CO_2:MeOH, 95:05, 2.5 mL min^{-1}, 40 °C, 220 nm) indicated 99% *ee*, t_R = 8.17 (minor), 9.10 (major) minutes.

$[\alpha]_D^{25}$ = –184° (c 0.49, $CHCl_3$).

(3a*S*,8a*R*)-4-Chloro-6-methoxy-2,3,3a,8a-tetrahydrofuro[2,3-*b*]benzofuran (5)

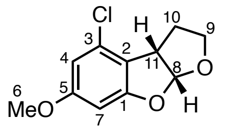

To a solution of (3a*S*,8a*R*)-4-chloro-2,3,3a,8a-tetrahydrofuro[2,3-*b*]benzofuran-6-ol (**4**) (31.5 mg, 0.148 mmol, 1.00 equiv.) in DMF (0.5 mL) was added K_2CO_3 (61.4 mg, 0.444 mmol, 3.00 equiv.). The reaction was stirred for 15 minutes followed by the addition of a solution of iodomethane (42.0 mg, 0.296 mmol, 2.00 equiv.) dissolved in DMF (0.5 mL). The reaction was stirred at room temperature for 2 days, after which it was judged complete by TLC. Water and CH_2Cl_2 were added, and the aqueous phase was extracted with CH_2Cl_2 (3×). The combined organic extracts were dried over Mg_2SO_4, and filtered through celite (eluting with EtOAc) to afford the title compound as an orange solid (33.5 mg, 0.148 mmol, quant., 99% *ee*).

^{1}H NMR (500 MHz, $CDCl_3$) δ 6.44 (d, *J* = 2.1 Hz, 1H, H_4), 6.33 (d, *J* = 5.7 Hz, 1H, H_8), 6.29 (dd, *J* = 2.1, 0.6 Hz, 1H, H_7), 4.10 (ddd, *J* = 8.7, 7.2, 1.1 Hz, 1H, H_9), 4.02 – 3.96 (m, 1H, H_{11}), 3.75 (s, 3H, H_6), 3.65 (ddd, *J* = 11.9, 8.7, 5.4 Hz, 1H, $H_{9'}$), 2.29 – 2.15 (m, 2H, H_{10}).

^{13}C NMR (126 MHz, $CDCl_3$) δ 161.4, 161.2, 130.8, 118.1, 112.0, 107.3, 94.7, 67.5, 55.9, 46.2, 31.7.

HRMS m/z: $[M+H]^+$ calculated for $[C_{11}H_{12}ClO_3]^+$ 227.0469, found 227.0473. Δ = +1.8 ppm.

Chiral SFC Analysis: CHIRALPAK IA (CO_2:MeOH, 99:01, 2.5 mL min^{-1}, 40 °C, 220 nm) indicated 99% *ee*, t_R = 5.95 (minor), 7.67 (major) minutes.

$[\alpha]_D^{25}$ = –140° (c 0.99, $CHCl_3$).

[N.B. The racemic SFC trace for the title compound was obtained through the reaction of chlorinated phenol **2g** with 2,3-dihydrofuran using (*rac*)-sSPhos i.e. analogous conditions to those in entry 3 of Table 2, but using a racemic ligand.]

(3a*S*,8a*R*)-6-Methoxy-2,3,3a,8a-tetrahydrofuro[2,3-*b*]benzofuran-4-ol (1)

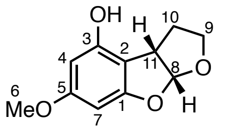

Reaction conditions were based on a report by Buchwald and co-workers.[11] A vial containing a magnetic stirrer bar was sequentially charged with (3a*S*,8a*R*)-4-chloro-6-methoxy-2,3,3a,8a-tetrahydrofuro[2,3-*b*]benzofuran (**5**) (12.0 mg, 0.0529 mmol, 1.00 equiv.), ^{t}BuXPhos (3.6 mg, 0.0085 mmol, 16 mol%), KOH (11.9 mg, 0.212 mmol, 4.00 equiv.), and Pd_2dba_3 (1.9 mg, 0.0021 mmol, 4.0 mol%). The vial was sealed, and evacuated and backfilled with nitrogen (4×). 1,4-Dioxane (0.1 mL) and water (0.1 mL) were added under nitrogen, and the reaction was stirred and heated to 100 °C in a preheated aluminum heating block for 3 days. Upon completion, sat. NH_4Cl (aq.) (0.3 mL) was added, and and the aqueous layer was extracted with chloroform (3 × 0.5 mL). The combined organic extracts were dried over $MgSO_4$ and purified by flash column chromatography (0-25% EtOAc in 40-60 °C petroleum ether) to afford the title compound as a colorless solid (8.4 mg, 0.040 mmol, 76% yield).

^{1}H NMR (700 MHz, $CDCl_3$) δ 6.32 (d, J = 5.7 Hz, 1H, H_8), 6.03 (d, J = 2.1 Hz, 1H, H_7), 5.90 (d, J = 2.1 Hz, 1H, H_4), 5.27 (br s, 1H, H_O), 4.08 (ddd, J = 8.6, 7.2, 1.1 Hz, 1H, H_9), 3.98 (ddd, J = 7.3, 5.7, 1.1 Hz, 1H, H_{11}), 3.72 (s, 3H, H_6), 3.67 (ddd, J = 11.7, 8.6, 5.2 Hz, 1H, $H_{9'}$), 2.22 – 2.09 (m, 2H, H_{10}).

^{13}C NMR (176 MHz, $CDCl_3$) δ 161.9, 161.8, 152.9, 112.1, 105.6, 95.1, 88.6, 67.6, 55.7, 44.2, 31.6.

^{1}H and ^{13}C data consistent with the literature.[12]

$[\alpha]_D^{25}$ = –109° (c 0.28, $CHCl_3$).

Both the sign and magnitude of the optical rotation of **1** were consistent with previous reports:

Literature values: $[\alpha]_D^{20}$ = –115.9° (c 0.55, $CHCl_3$) for 94% *ee*, same enantiomer.[13] $[\alpha]_D^{23}$ = −148° (c 0.5, $CHCl_3$), same enantiomer.[14] $[\alpha]_D^{25}$ = –149.9° (c 0.92, $CHCl_3$), same enantiomer.[12]

Characterization Data for Products in Scheme 5

General Procedure B: Palladium/(*R*)-sSPhos-Catalyzed Carboamination of 2,3-Dihydrofuran with 2-Bromoanilines

A vial containing a magnetic stirrer bar was sequentially charged with the relevant Ts-protected 2-bromoaniline (0.200 mmol, 1.00 equiv.), (*R*)-sSPhos (5.1 mg, 0.010 mmol, 5.0 mol%), NaOH (12.0 mg, 0.300 mmol, 1.50 equiv.), and Pd_2dba_3 (4.6 mg, 0.0050 mmol, 2.5 mol%). The vial was sealed, and evacuated and backfilled with nitrogen (4×). 2,3-Dihydrofuran (70.1 mg. 75.6 µL, 1.00 mmol, 5.00 equiv.) was added under nitrogen. PhMe (0.66 mL) and *tert*-amyl alcohol (0.33 mL) were added under nitrogen. The reaction was stirred at 80 °C and 900 rpm in a preheated aluminum heating block for 16 h. Upon completion, the reaction mixture was filtered through celite, eluting with EtOAc. The filtrate was concentrated under a stream of air. The crude product was purified by flash column chromatography.

Determination of Absolute Stereochemistry for Carboamination Products with 2-Bromoanilines

The optical rotation of **7a** was compared to that reported by Zhang and co-workers, enabling absolute stereochemistry of **7a** to be assigned.[9] Stereochemistry of the remaining products in Scheme 5 (**7b**, **7c**) are assigned by analogy.

As expected, the absolute configurations of products arising from carboamination and carboetherification of the 2-bromophenol and 2-bromoaniline series respectively were found to be consistent in terms of the face of approach of 2,3-dihydrofuran.

(3a*S*,8a*S*)-8-Tosyl-3,3a,8,8a-tetrahydro-2*H*-furo[2,3-*b*]indole (7a)

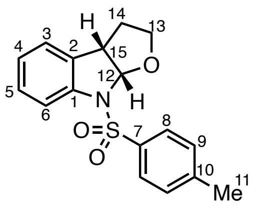

Prepared according to General Procedure B with *N*-(2-bromophenyl)-4-methylbenzenesulfonamide (**6a**) (65.2 mg, 0.200 mmol) as the starting material. Purification by flash column chromatography (0-20% EtOAc in 40-60 °C petroleum ether) afforded the title compound as a colorless solid (58.0 mg, 0.184 mmol, 92% yield, 86% *ee*).

^{1}H NMR (500 MHz, $CDCl_3$) δ 7.89 – 7.83 (m, 2H, H_8), 7.36 (d, *J* = 8.1 Hz, 1H, H_6), 7.25 – 7.22 (m, 2H, H_9), 7.19 – 7.14 (m, 1H, H_5), 7.13 (d, *J* = 7.5 Hz, 1H, H_3), 6.98 (app td, *J* = 7.5, 1.0 Hz, 1H, H_4), 6.26 (d, *J* = 6.6 Hz, 1H, H_{12}), 3.97 (ddd, *J* = 8.7, 7.4, 1.0 Hz, 1H, H_{13}), 3.90 (app t, *J* = 7.6 Hz, 1H, H_{15}), 3.31 (ddd, *J* = 11.8, 8.7, 4.8 Hz, 1H, $H_{13'}$), 2.37 (s, 3H, H_{11}), 2.34 – 2.24 (m, 1H, H_{14}), 2.01 (dd, *J* = 12.3, 4.8 Hz, 1H, $H_{14'}$).

^{13}C NMR (126 MHz, $CDCl_3$) δ 144.0, 141.6, 136.7, 131.5, 129.7, 128.5, 127.5, 125.0, 123.6, 112.9, 95.9, 66.5, 45.6, 33.8, 21.6.

Chiral SFC Analysis: CHIRALPAK IJ (CO_2:MeOH, 90:10, 2.5 mL min^{-1}, 40 °C, 220 nm) indicated 86% *ee*, t_R = 5.27 (minor), 6.23 (major) minutes.

$[\alpha]_D^{25}$ = –20.6° (c 1.45, $CHCl_3$). Literature value: **$[\alpha]_D^{20}$** = +24.960° (c 0.625, $CHCl_3$) for 96% *ee*, opposite enantiomer.[9]

^{1}H and ^{13}C data consistent with the literature.[9]

(3a*S*,8a*S*)-5-Methoxy-8-tosyl-3,3a,8,8a-tetrahydro-2*H*-furo[2,3-*b*]indole (7b)

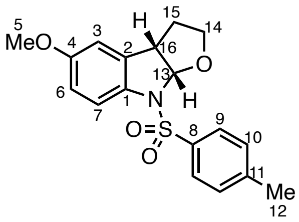

Prepared according to General Procedure B with *N*-(2-bromo-4-methoxyphenyl)-4-methylbenzenesulfonamide (**6b**) (71.2 mg, 0.200 mmol) as the starting material. Purification by flash column chromatography (0-30% EtOAc in 40-60 °C petroleum ether) afforded the title compound as a colorless solid (42.0 mg, 0.122 mmol, 61% yield, 88% *ee*).

^{1}H NMR (500 MHz, $CDCl_3$) δ 7.81 – 7.75 (m, 2H, H_9), 7.33 (d, *J* = 8.8 Hz, 1H, H_7), 7.24 – 7.19 (m, 2H, H_{10}), 6.71 (dd, *J* = 8.8, 2.6, 1H, H_6), 6.67 (dd, *J* = 2.6, 1.0 Hz, 1H, H_3), 6.17 (d, *J* = 6.6 Hz, 1H, H_{13}), 3.96 (ddd, *J* = 8.7, 7.5, 1.1 Hz, 1H, H_{14}), 3.80 (dd, *J* = 8.5, 6.6 Hz, 1H, H_{16}), 3.74

(s, 3H, H_5), 3.34 (ddd, J = 11.9, 8.7, 4.8 Hz, 1H, $H_{14'}$), 2.36 (s, 3H, H_{12}), 2.25 (app tdd, J = 11.9, 8.5, 7.5 Hz, 1H, H_{15}), 1.99 (dd, J = 11.9, 4.8 Hz, 1H, $H_{15'}$).

^{13}C NMR (126 MHz, $CDCl_3$) δ 156.8, 143.9, 136.4, 135.2, 133.3, 129.7, 127.4, 114.3, 113.4, 111.0, 96.3, 66.6, 55.8, 45.8, 33.7, 21.6.

Chiral SFC Analysis: CHIRALPAK IJ (CO_2:MeOH, 90:10, 2.5 mL min^{-1}, 40 °C, 220 nm) indicated 88% *ee*, t_R = 6.85 (minor), 8.16 (major) minutes.

$[\alpha]_D^{25}$ = –62.2° (c 2.10, $CHCl_3$).

Data consistent with the literature.[9]

(3a*S*,8a*S*)-8-Tosyl-5-(trifluoromethyl)-3,3a,8,8a-tetrahydro-2*H*-furo[2,3-*b*]indole (7c)

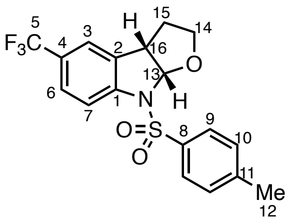

Prepared according to General Procedure B with *N*-(2-bromo-4-(trifluoromethyl)phenyl)-4-methylbenzenesulfonamide (**6c**) (78.8 mg, 0.200 mmol) as the starting material. Purification by flash column chromatography (0-20% EtOAc in 40-60 °C petroleum ether) afforded the title compound as a pink solid (72.4 mg, 0.189 mmol, 94% yield, 93% *ee*).

^{1}H NMR (700 MHz, $CDCl_3$) δ 7.91 – 7.85 (m, 2H, H_9), 7.45 – 7.40 (m, 2H, H_6 and H_7), 7.39 – 7.36 (m, 1H, H_3), 7.29 – 7.26 (m, 2H, H_{10}), 6.35 (d, J = 6.6 Hz, 1H, H_{13}), 4.04 – 3.91 (m, 2H, 1×H_{14} and H_{16}), 3.28 (ddd, J = 11.9, 9.0, 4.8 Hz, 1H, $H_{14'}$), 2.39 (s, 3H, H_{12}), 2.37 – 2.30 (m, 1H, H_{15}), 2.04 (dd, J = 12.3, 4.8 Hz, 1H, $H_{15'}$).

^{13}C NMR (176 MHz, $CDCl_3$) δ 144.5 (2×), 136.4, 132.2, 129.9, 127.6, 126.2 (q, J = 4.0 Hz), 125.7 (q, J = 32.7 Hz), 124.2 (q, J = 271.4 Hz), 122.2 (q, J = 4.3 Hz), 112.3, 96.3, 66.5, 45.4, 33.8, 21.7.

^{19}F NMR (376 MHz, $CDCl_3$) δ –61.6.

Chiral SFC Analysis: CHIRALPAK IJ (CO_2:MeOH, 90:10, 2.5 mL min^{-1}, 40 °C, 220 nm) indicated 93% *ee*, t_R = 3.38 (minor), 3.62 (major) minutes.

[α]$_D$^{25} = +3.0° (c 1.81, $CHCl_3$). Literature value: **[α]$_D$^{20}** = +4.896° (c 0.625, $CHCl_3$) for 94% *ee*, opposite enantiomer.[9] [N.B. The same sign of the optical rotation between the literature and measured values would normally imply formation of the same enantiomer. However, we haven't concluded this for **7c** because of the low magnitudes of the optical rotations observed. It seems likely that trace impurities with much larger optical rotation magnitudes than the title compound are affecting the sign of the observed optical rotation.

We reasoned that assigning the absolute configuration in analogy with other scope entries, for which several data points comparing optical rotations with the literature exist, provides better evidence for the enantiomer of **7c** formed than comparing the optical rotation of **7c** with the one reported literature value.

^{1}H, ^{13}C, and ^{19}F data consistent with the literature.[9]

References

1. Pearce-Higgins, R.; Hogenhout, L. N.; Docherty, P. J.; Whalley, D. M.; Chuentragool, P.; Lee, N.; Lam, N. Y. S.; McGuire, T. M.; Valette, D.; Phipps, R. J., An Enantioselective Suzuki–Miyaura Coupling To Form Axially Chiral Biphenols. *J. Am. Chem. Soc.* **2022,** *144*, 15026-15032.
2. Kadarauch, M.; Whalley, D. M.; Phipps, R. J., sSPhos: A General Ligand for Enantioselective Arylative Phenol Dearomatization via Electrostatically-Directed Palladium Catalysis. *J. Am. Chem. Soc.* **2023,** *145*, 25553-25558.
3. Zalesskiy, S. S.; Ananikov, V. P., $Pd_2(dba)_3$ as a Precursor of Soluble Metal Complexes and Nanoparticles: Determination of Palladium Active Species for Catalysis and Synthesis. *Organometallics* **2012,** *31*, 2302-2309.
4. Liwosz, T. W.; Chemler, S. R., Copper-Catalyzed Oxidative Amination and Allylic Amination of Alkenes. *Chem. Eur. J.* **2013,** *19*, 12771-12777.
5. Fujita, T.; Sugiyama, K.; Sanada, S.; Ichitsuka, T.; Ichikawa, J., Platform for Ring-Fluorinated Benzoheterole Derivatives: Palladium-Catalyzed Regioselective 1,1-Difluoroallylation and Heck Cyclization. *Org. Lett.* **2016,** *18*, 248-251.
6. Vaith, J.; Rodina, D.; Spaulding, G. C.; Paradine, S. M., Pd-Catalyzed Heteroannulation Using N-Arylureas as a Sterically Undemanding Ligand Platform. *J. Am. Chem. Soc.* **2022,** *144*, 6667-6673.
7. Gou, H.; Zhang, J.; Li, P.; Li, C.; Wang, H.; Hong, W., A practical total synthesis of wedelolactone. *Synth. Commun.* **2023,** *53*, 1126-1133.
8. Borrajo-Calleja, G. M.; Bizet, V.; Mazet, C., Palladium-Catalyzed Enantioselective Intermolecular Carboetherification of Dihydrofurans. *J. Am. Chem. Soc.* **2016,** *138*, 4014-4017.
9. Tao, M.; Tu, Y.; Liu, Y.; Wu, H.; Liu, L.; Zhang, J., Pd/Xiang-Phos-catalyzed enantioselective intermolecular carboheterofunctionalization under mild conditions. *Chem. Sci.* **2020,** *11*, 6283-6288.
10. Borrajo-Calleja, G. M.; Bizet, V.; Besnard, C.; Mazet, C., Mechanistic Investigation of the Pd-Catalyzed Intermolecular Carboetherification and Carboamination of 2,3-

Dihydrofuran: Similarities, Differences, and Evidence for Unusual Reaction Intermediates. *Organometallics* **2017,** *36*, 3553-3563.
11. Anderson, K. W.; Ikawa, T.; Tundel, R. E.; Buchwald, S. L., The Selective Reaction of Aryl Halides with KOH: Synthesis of Phenols, Aromatic Ethers, and Benzofurans. *J. Am. Chem. Soc.* **2006,** *128*, 10694-10695.
12. Huang, W.-L.; Raja, A.; Hong, B.-C.; Lee, G.-H., Organocatalytic Enantioselective Michael–Acetalization–Reduction–Nef Reaction for a One-Pot Entry to the Functionalized Aflatoxin System. Total Synthesis of (−)- Dihydroaflatoxin D2 and (−)- and (+)-Microminutinin. *Org. Lett.* **2017,** *19*, 3494-3497.
13. Falcone, N. A.; Bosse, A. T.; Park, H.; Yu, J.-Q.; Davies, H. M. L.; Sorensen, E. J., A C–H Functionalization Strategy Enables an Enantioselective Formal Synthesis of (−)-Aflatoxin B2. *Org. Lett.* **2021,** *23*, 9393-9397.
14. Zhou, G.; Corey, E. J., Short, Enantioselective Total Synthesis of Aflatoxin B2 Using an Asymmetric [3+2]-Cycloaddition Step. *J. Am. Chem. Soc.* **2005,** *127*, 11958-11959.

(3a*S*,8a*R*)-2,3,3a,8a-Tetrahydrofuro[2,3-*b*]benzofuran (3a)

Chiral SFC Analysis: CHIRALPAK IK (CO_2:MeOH, 99:01, 2.5 mL min^{-1}, 40 °C, 220 nm) indicated 98% *ee*, t_R = 4.80 (major), 5.14 (minor) minutes.

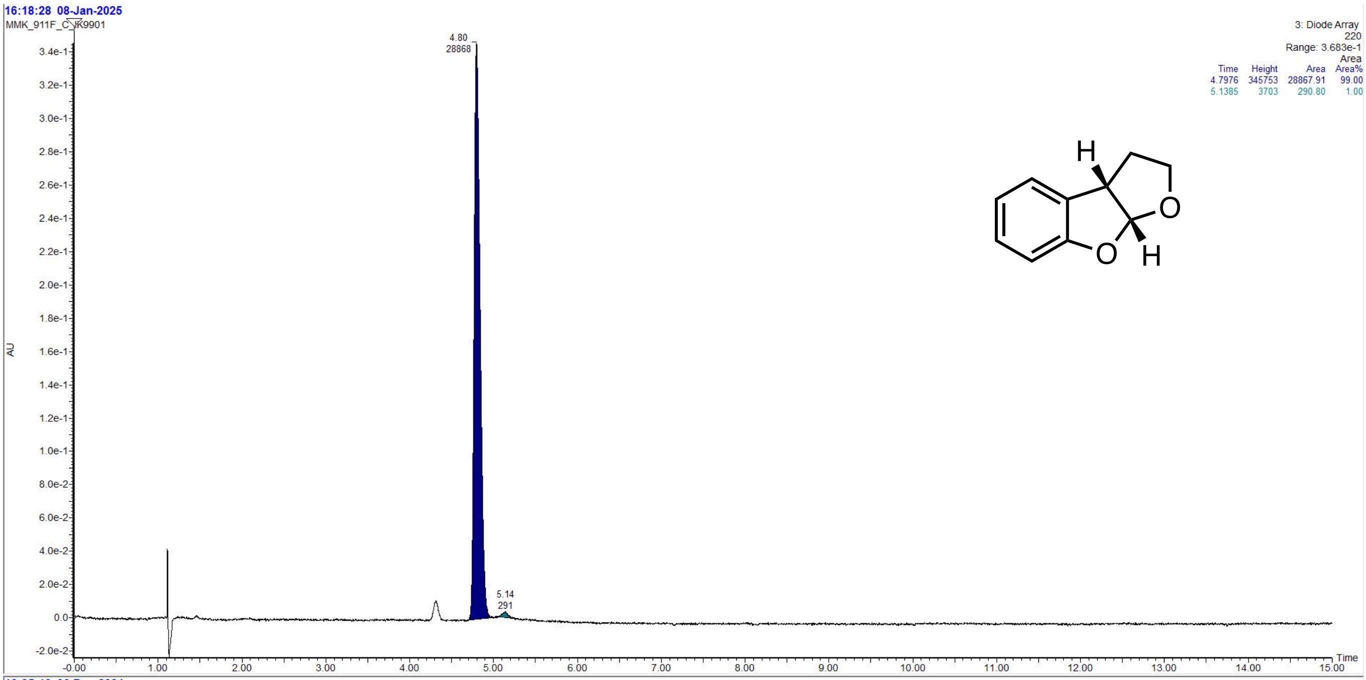

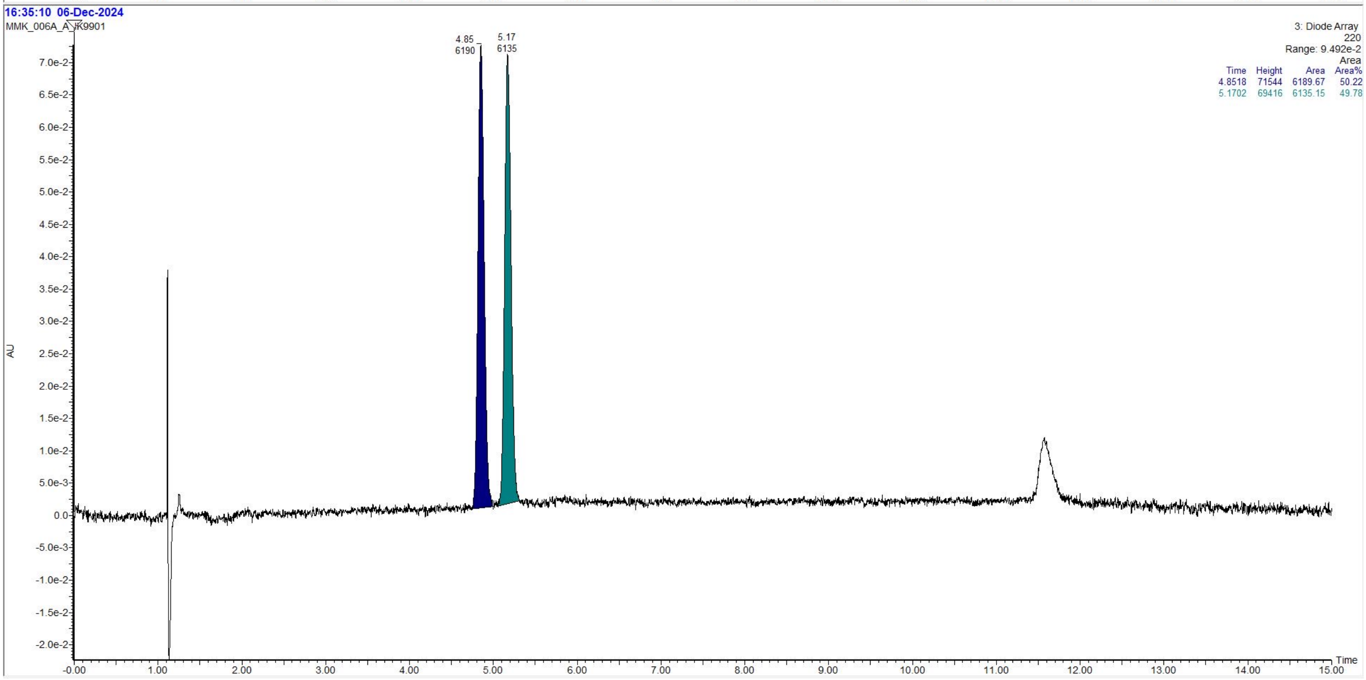

(3a*S*,8a*R*)-5-Methoxy-2,3,3a,8a-tetrahydrofuro[2,3-*b*]benzofuran (3b)

Chiral SFC Analysis: CHIRALPAK IJ (CO_2:MeOH, 95:05, 2.5 mL min^{-1}, 40 °C, 220 nm) indicated 99% *ee*, t_R = 3.22 (major), 3.61 (minor) minutes.

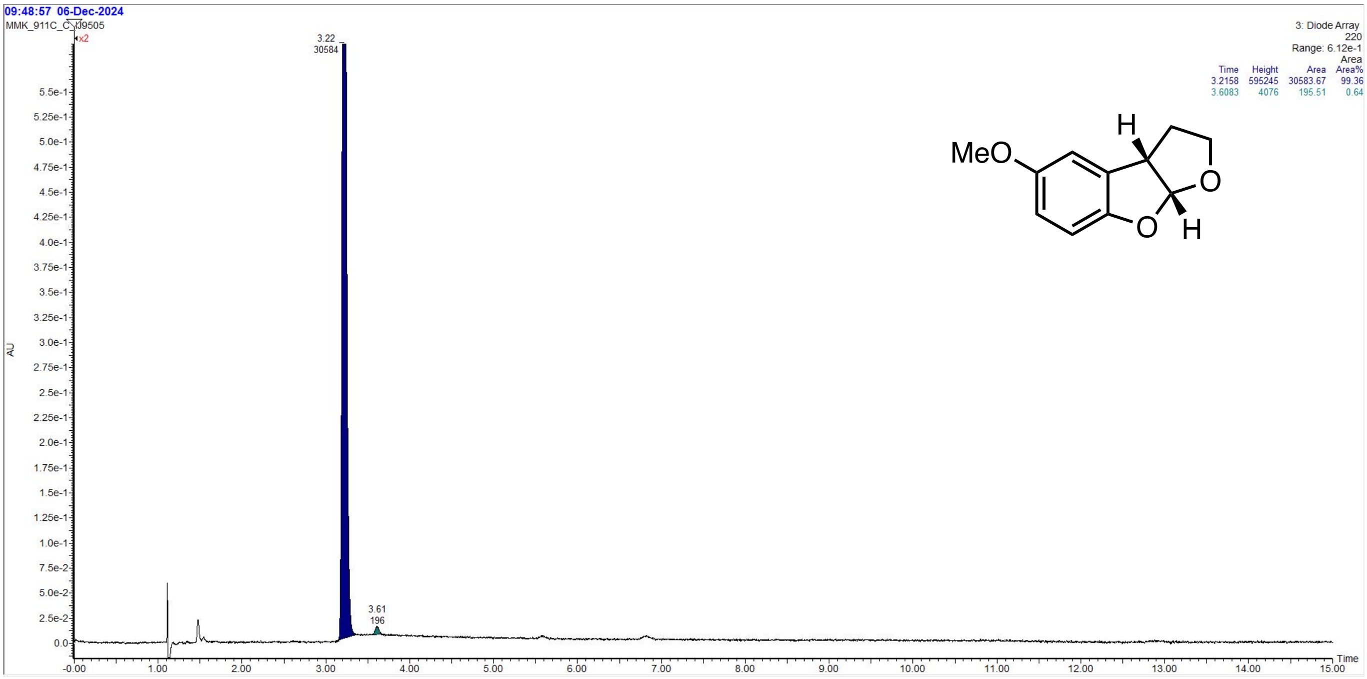

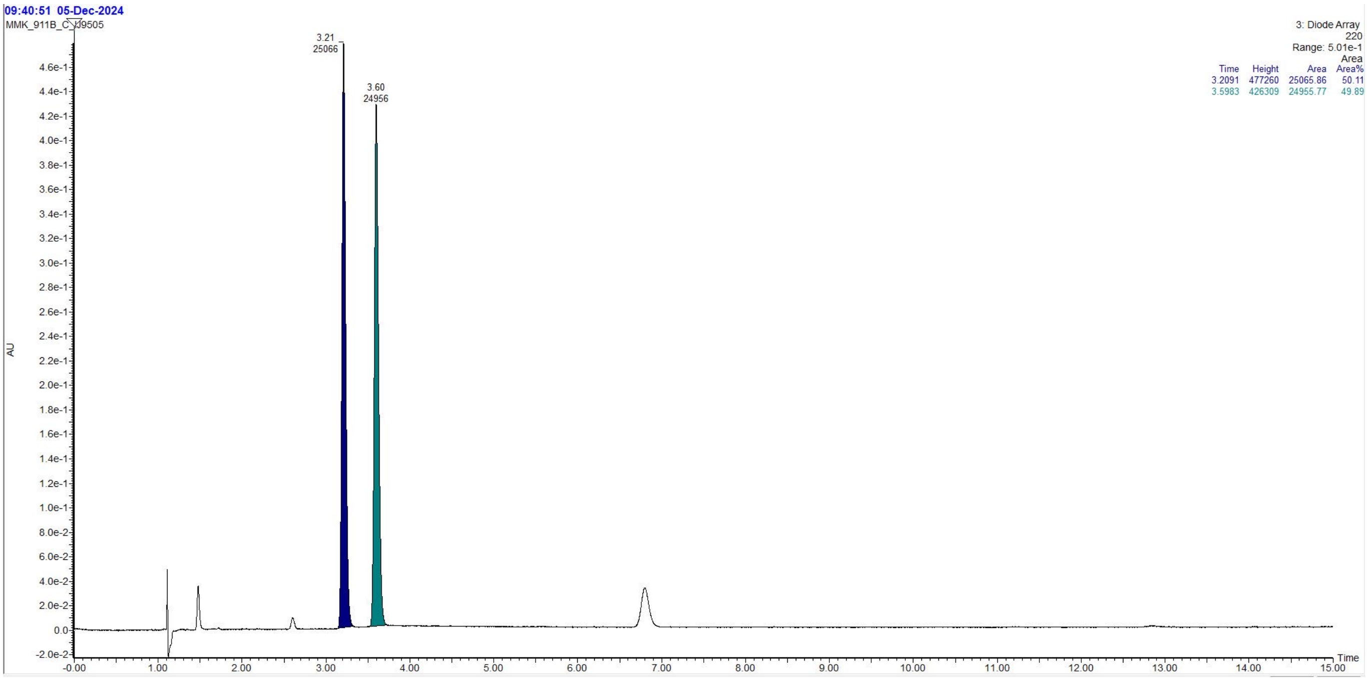

(3a*S*,8a*R*)-5-(trifluoromethyl)-2,3,3a,8a-Tetrahydrofuro[2,3-*b*]benzofuran (3c)

Chiral SFC Analysis: CHIRALPAK IJ (CO_2:MeOH, 99:01, 2.5 mL min^{-1}, 40 °C, 230 nm) indicated 94% *ee*, t_R = 2.17 (major), 2.65 (minor) minutes.

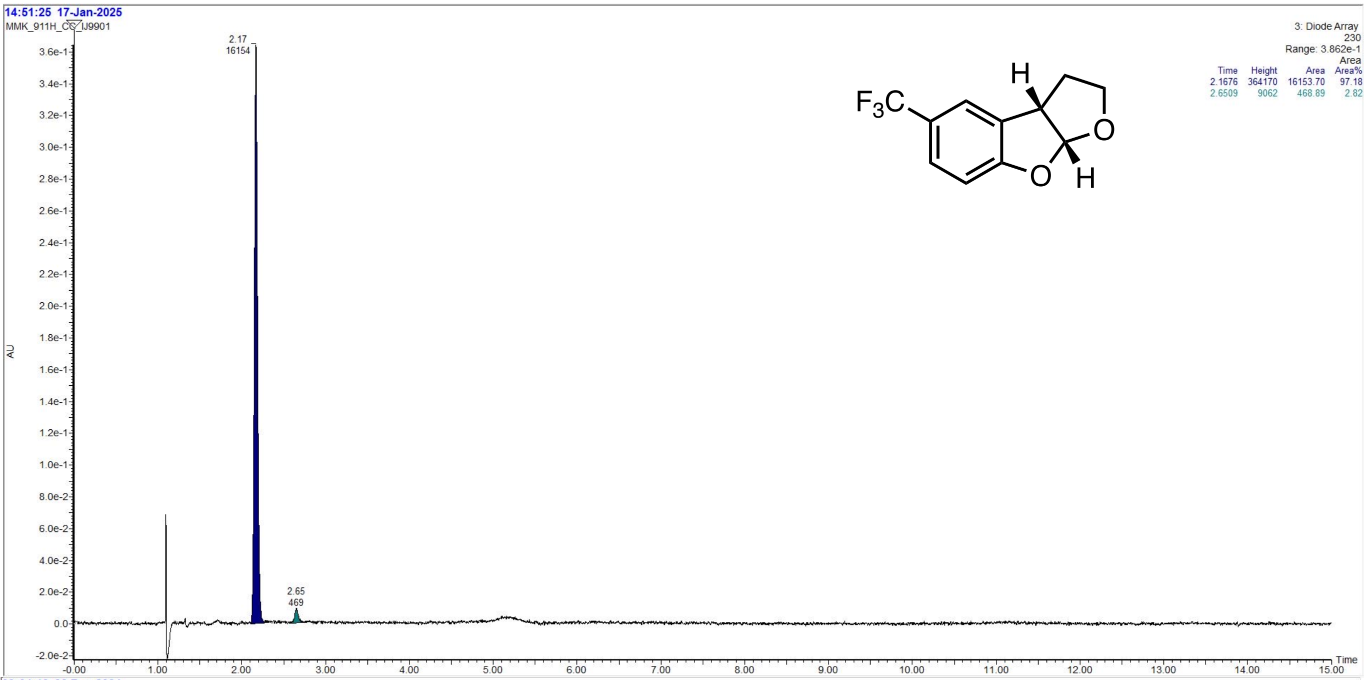

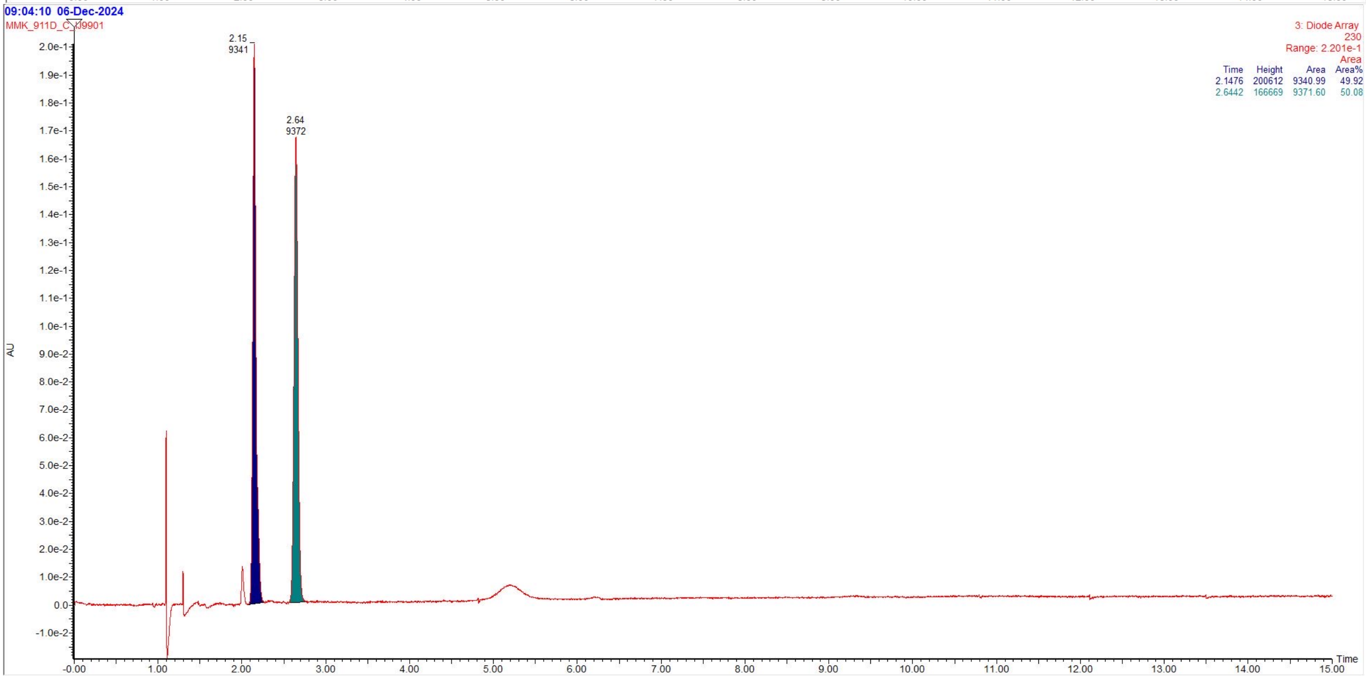

(3a*S*,8a*R*)-4-Methyl-2,3,3a,8a-tetrahydrofuro[2,3-*b*]benzofuran (3d)

Chiral SFC Analysis: CHIRALPAK IK (CO_2:MeOH, 99:01, 2.5 mL min^{-1}, 40 °C, 220 nm) indicated >99% *ee*, t_R = 6.03 (major), 6.63 (minor) minutes.

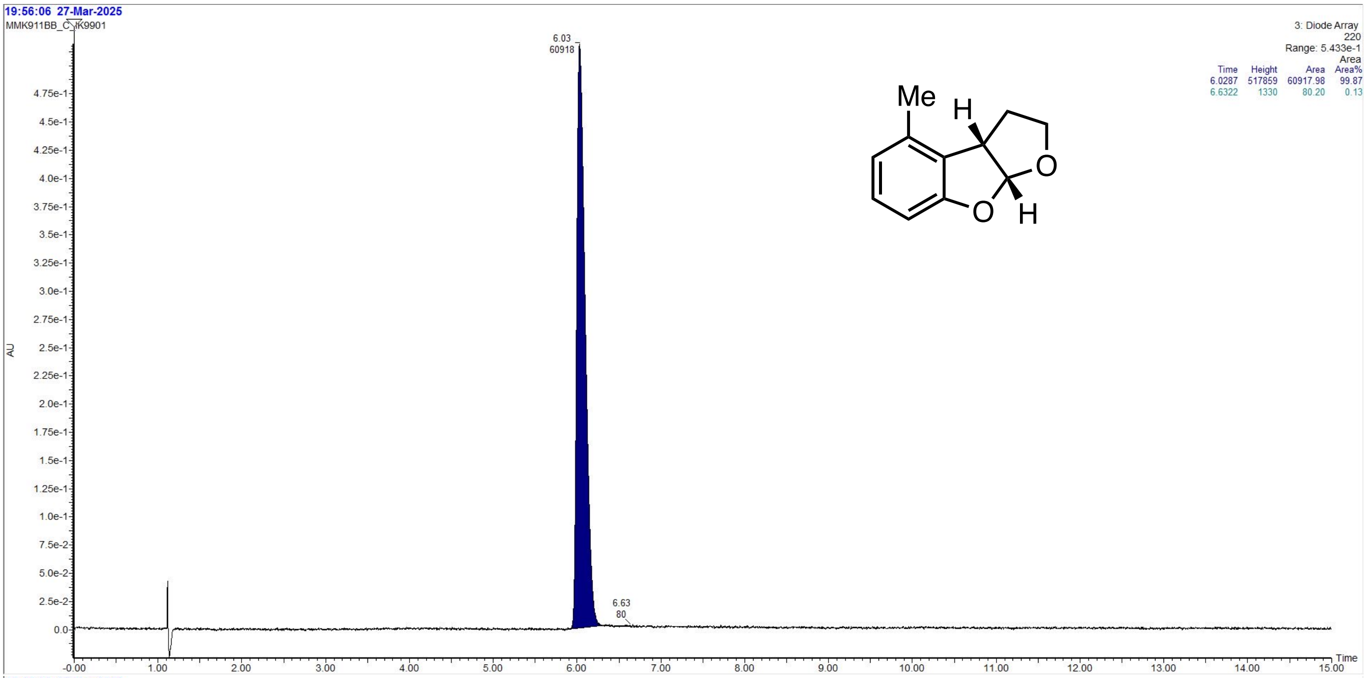

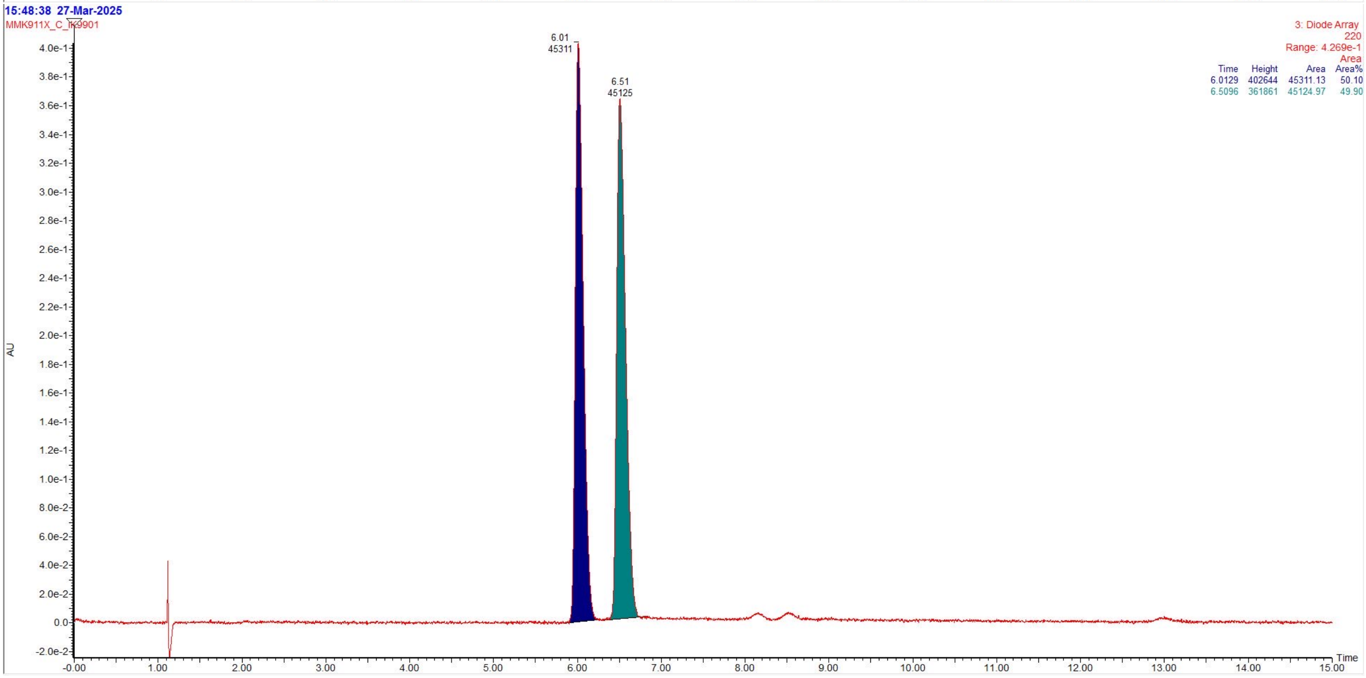

(3a*S*,8a*R*)-4-Chloro-2,3,3a,8a-tetrahydrofuro[2,3-*b*]benzofuran (3h)

Chiral SFC Analysis: CHIRALPAK IJ (CO_2:MeOH, 99:01, 2.5 mL min^{-1}, 40 °C, 220 nm) indicated 99% *ee*, t_R = 3.52 (major), 3.72 (minor) minutes.

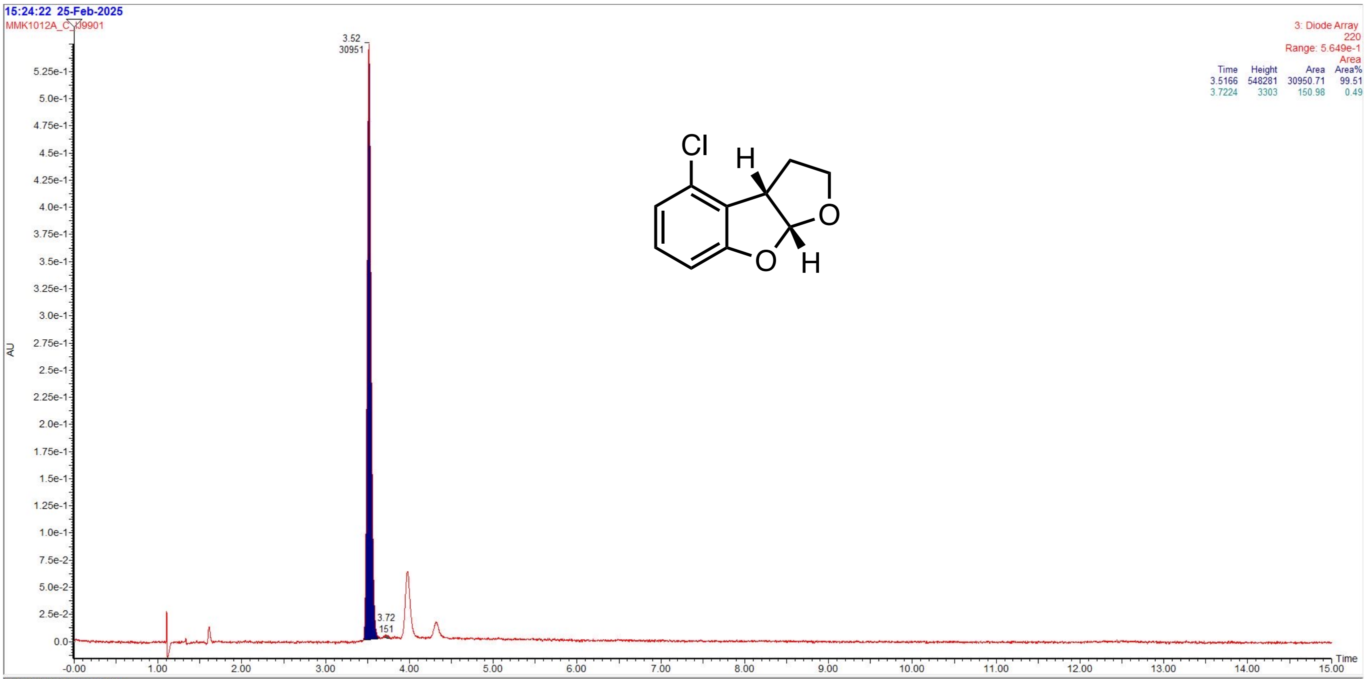

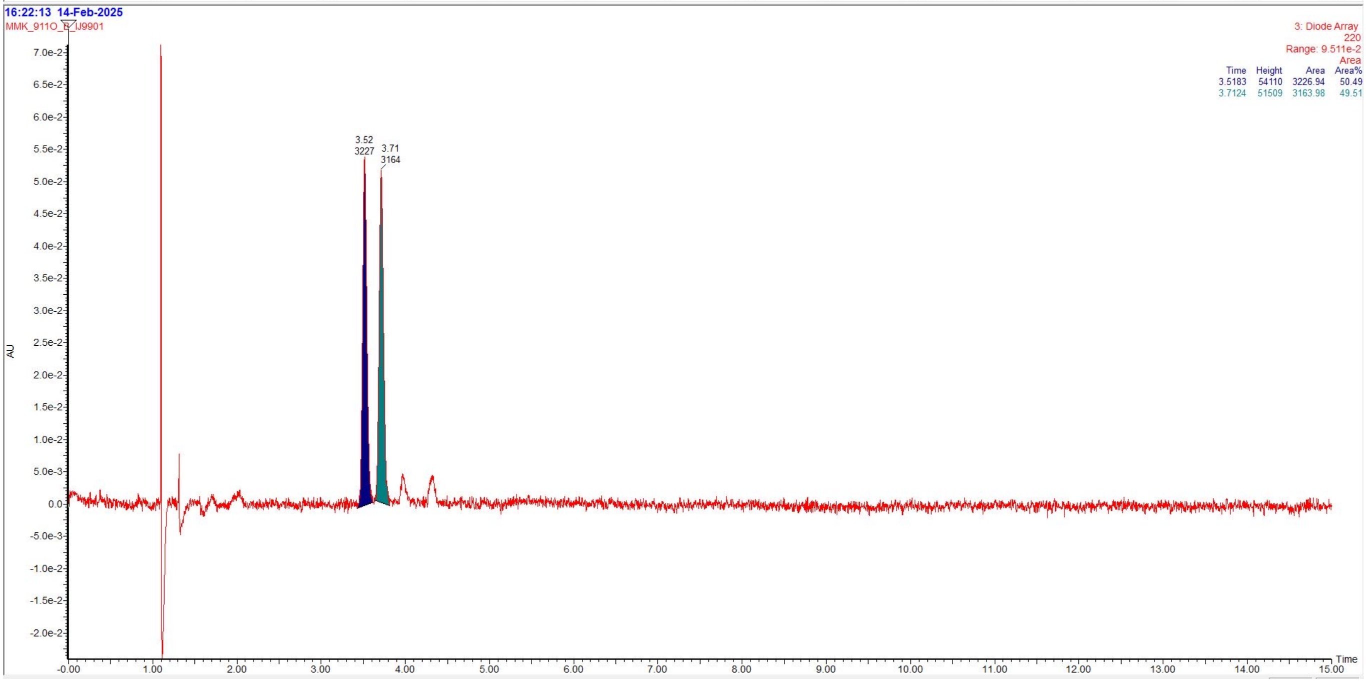

(3a*S*,8a*R*)-4-Chloro-2,3,3a,8a-tetrahydrofuro[2,3-*b*]benzofuran-6-ol (4)

Chiral SFC Analysis: CHIRALPAK IE (CO_2:MeOH, 95:05, 2.5 mL min^{-1}, 40 °C, 220 nm) indicated 99% *ee*, t_R = 8.17 (minor), 9.10 (major) minutes.

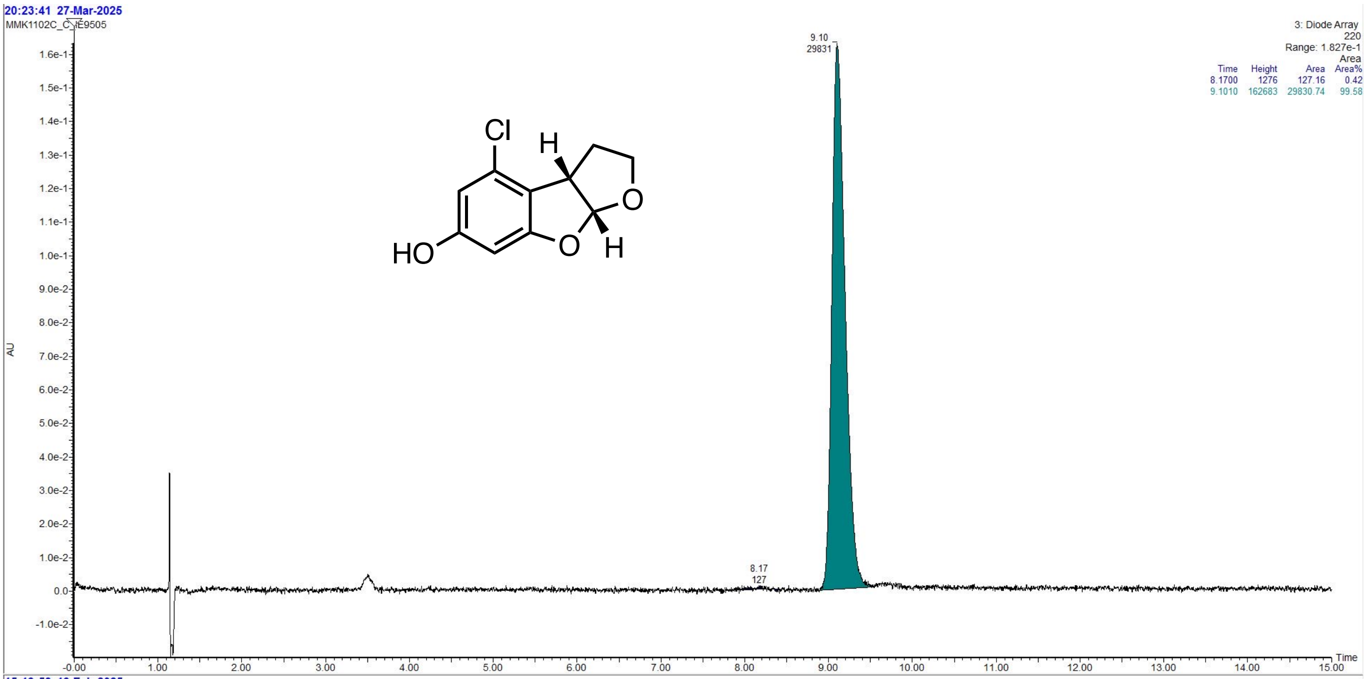

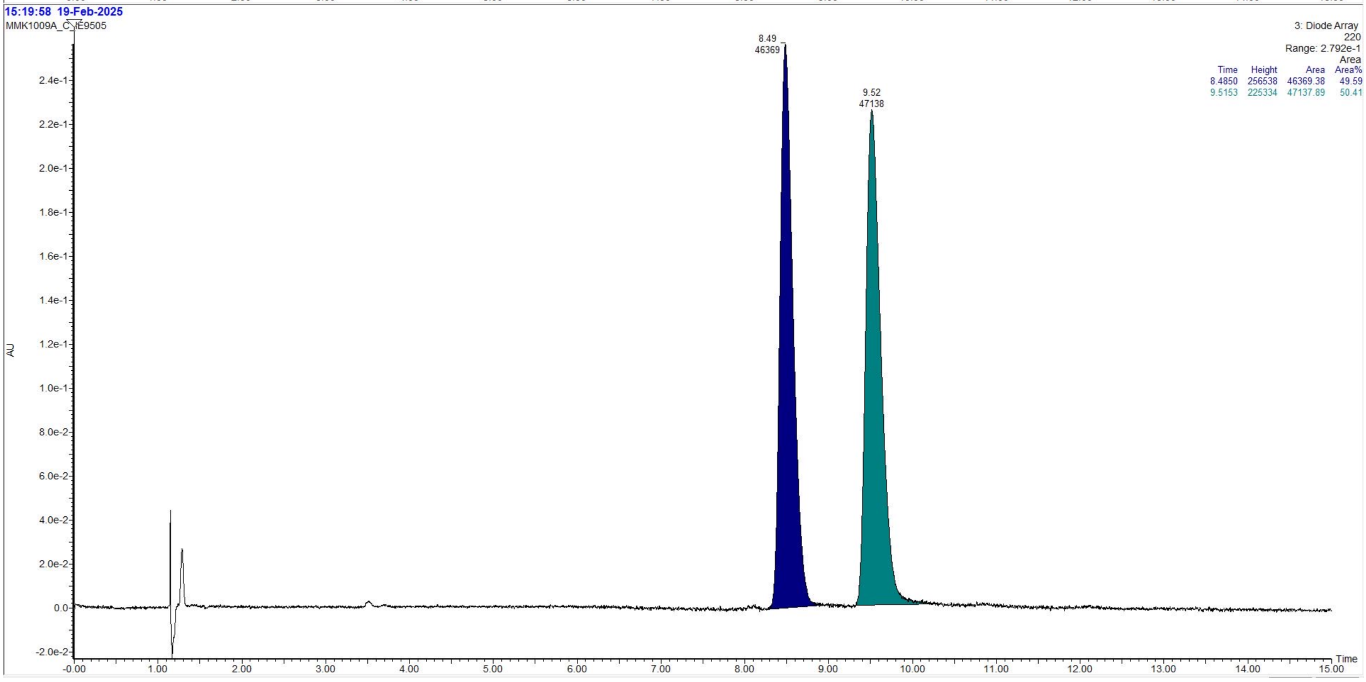

(3a*S*,8a*R*)-4-Chloro-6-methoxy-2,3,3a,8a-tetrahydrofuro[2,3-*b*]benzofuran (5)

Chiral SFC Analysis: CHIRALPAK IA (CO_2:MeOH, 99:01, 2.5 mL min^{-1}, 40 °C, 220 nm) indicated 99% *ee*, t_R = 5.95 (minor), 7.67 (major) minutes.

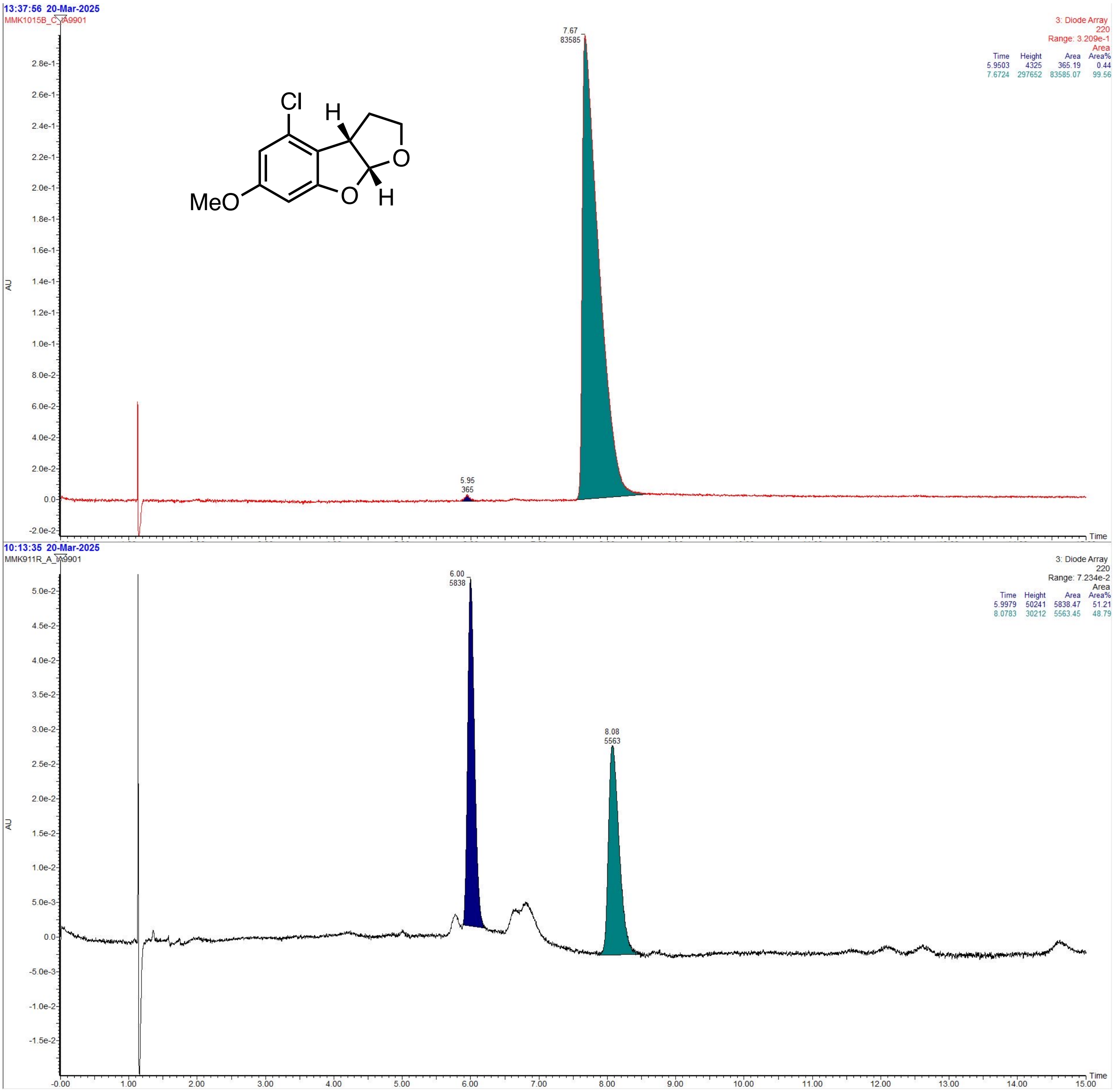

(3a*S*,8a*S*)-8-Tosyl-3,3a,8,8a-tetrahydro-2*H*-furo[2,3-*b*]indole (7a)

Chiral SFC Analysis: CHIRALPAK IJ (CO_2:MeOH, 90:10, 2.5 mL min^{-1}, 40 °C, 220 nm) indicated 86% *ee*, t_R = 5.27 (minor), 6.23 (major) minutes.

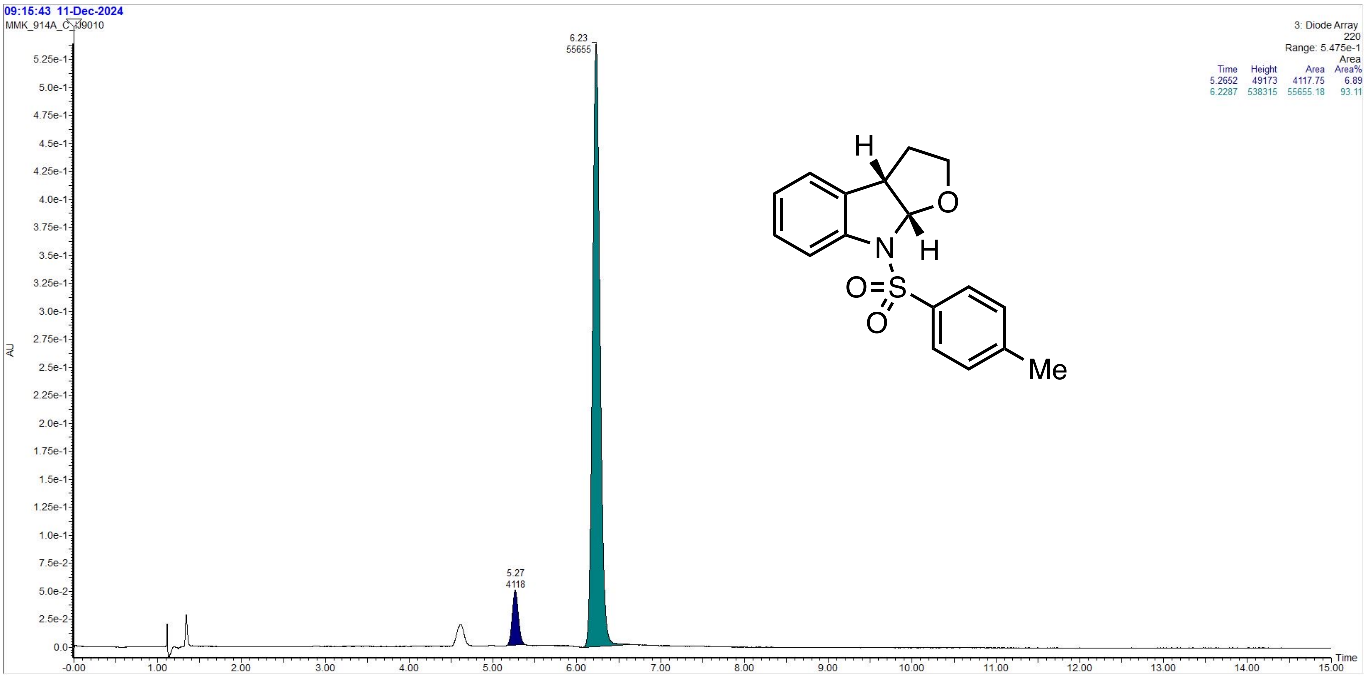

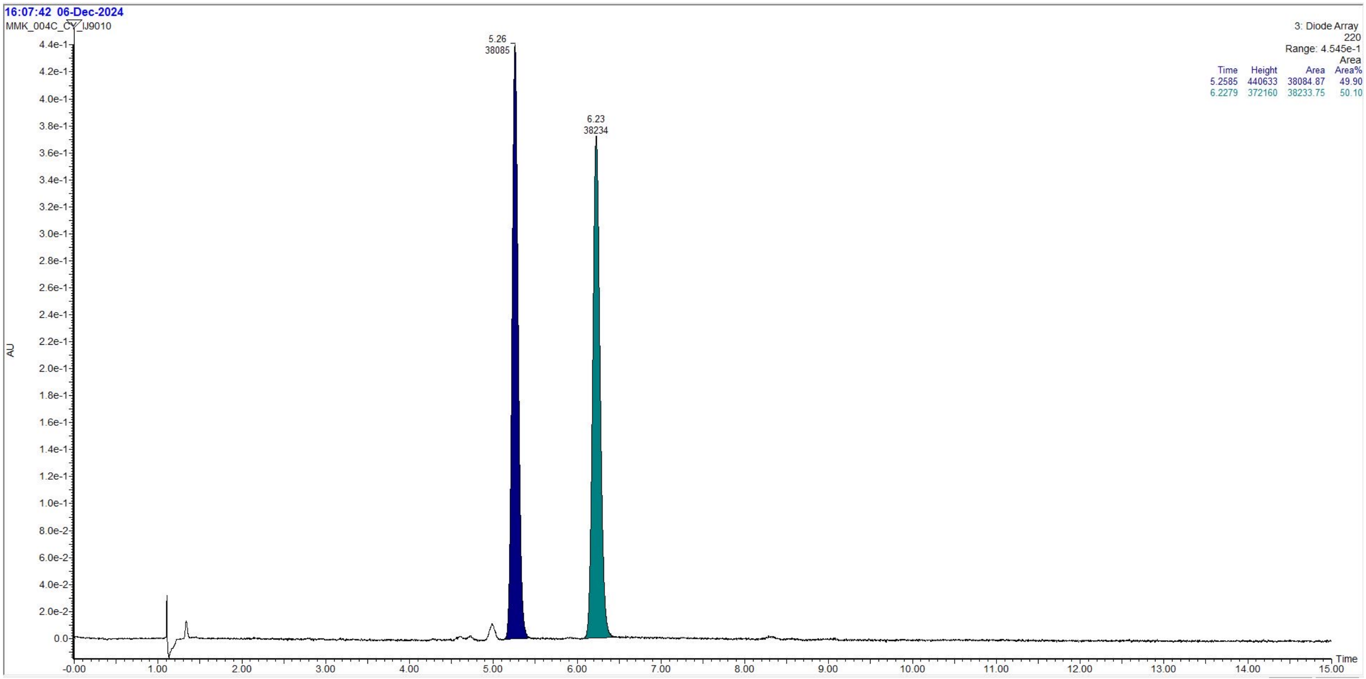

(3a*S*,8a*S*)-5-Methoxy-8-tosyl-3,3a,8,8a-tetrahydro-2*H*-furo[2,3-*b*]indole (7b)

Chiral SFC Analysis: CHIRALPAK IJ (CO_2:MeOH, 90:10, 2.5 mL min^{-1}, 40 °C, 220 nm) indicated 88% *ee*, t_R = 6.85 (minor), 8.16 (major) minutes.

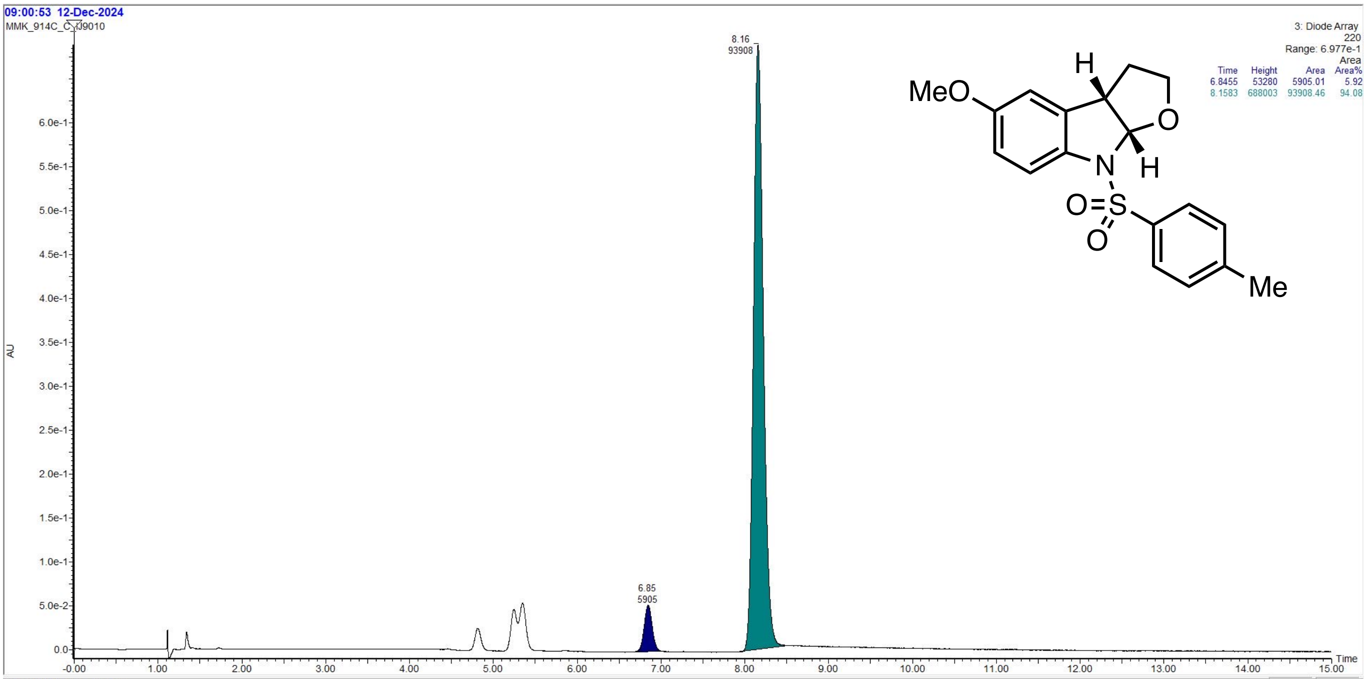

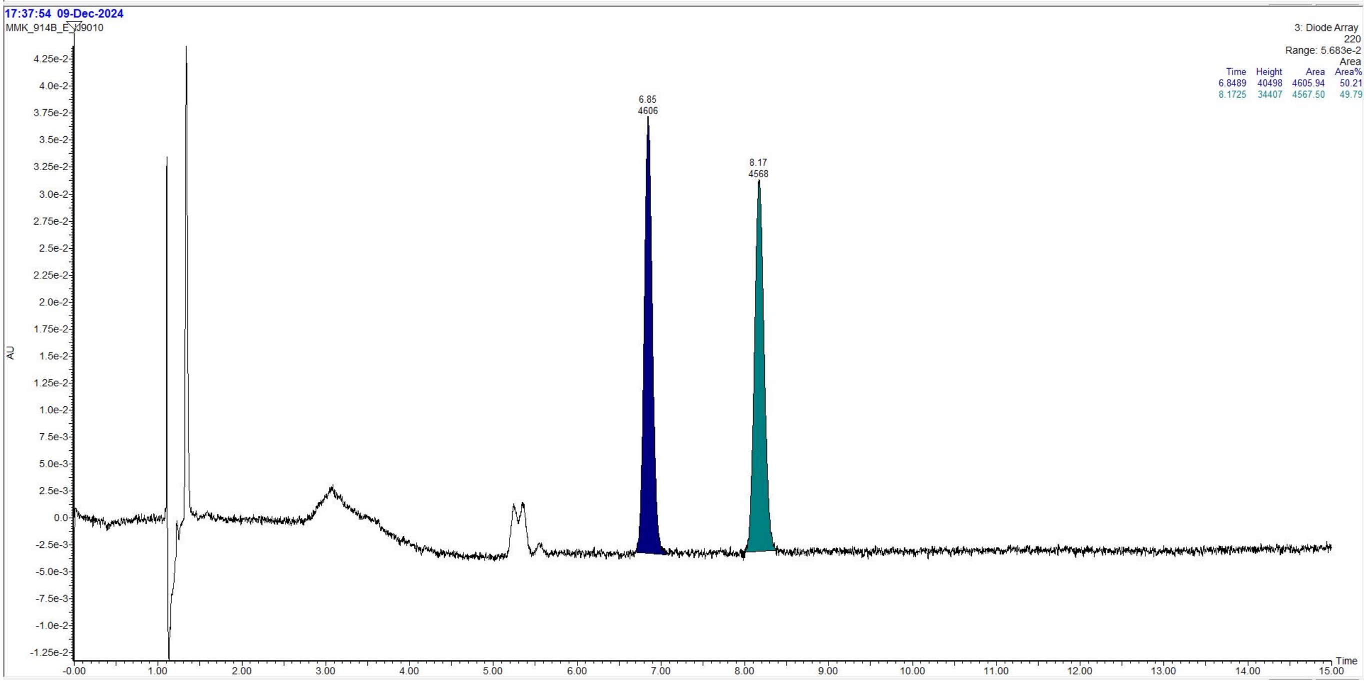

(3a*S*,8a*S*)-8-Tosyl-5-(trifluoromethyl)-3,3a,8,8a-tetrahydro-2*H*-furo[2,3-*b*]indole (7c)

Chiral SFC Analysis: CHIRALPAK IJ (CO_2:MeOH, 90:10, 2.5 mL min^{-1}, 40 °C, 220 nm) indicated 93% *ee*, t_R = 3.38 (minor), 3.62 (major) minutes.

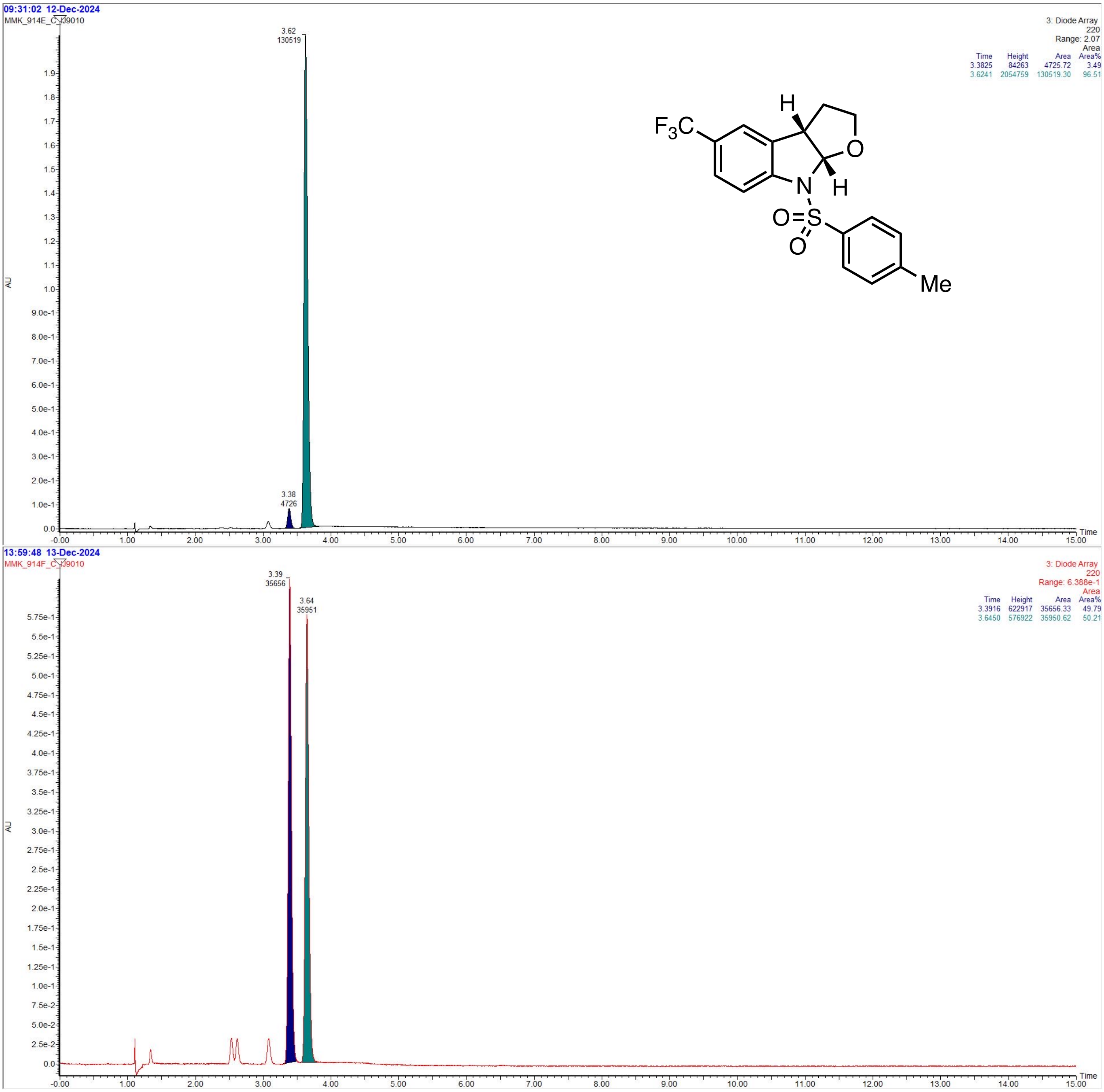

^{1}H NMR (400 MHz, $CDCl_3$): 3-(benzyloxy)-2-Bromo-5-methoxyphenol (2f)

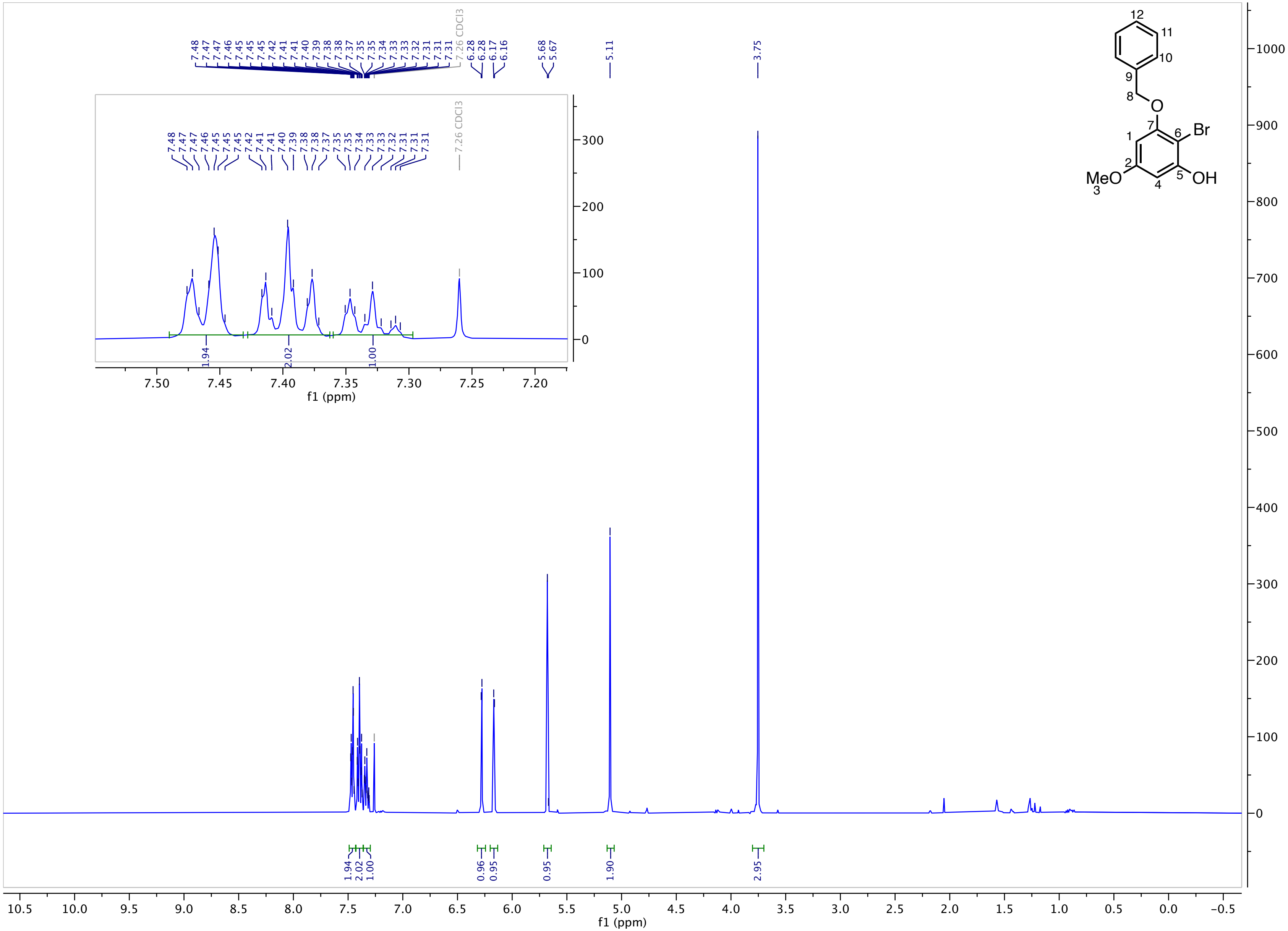

^{13}C NMR (101 MHz, $CDCl_3$): 3-(benzyloxy)-2-Bromo-5-methoxyphenol (**2f**)

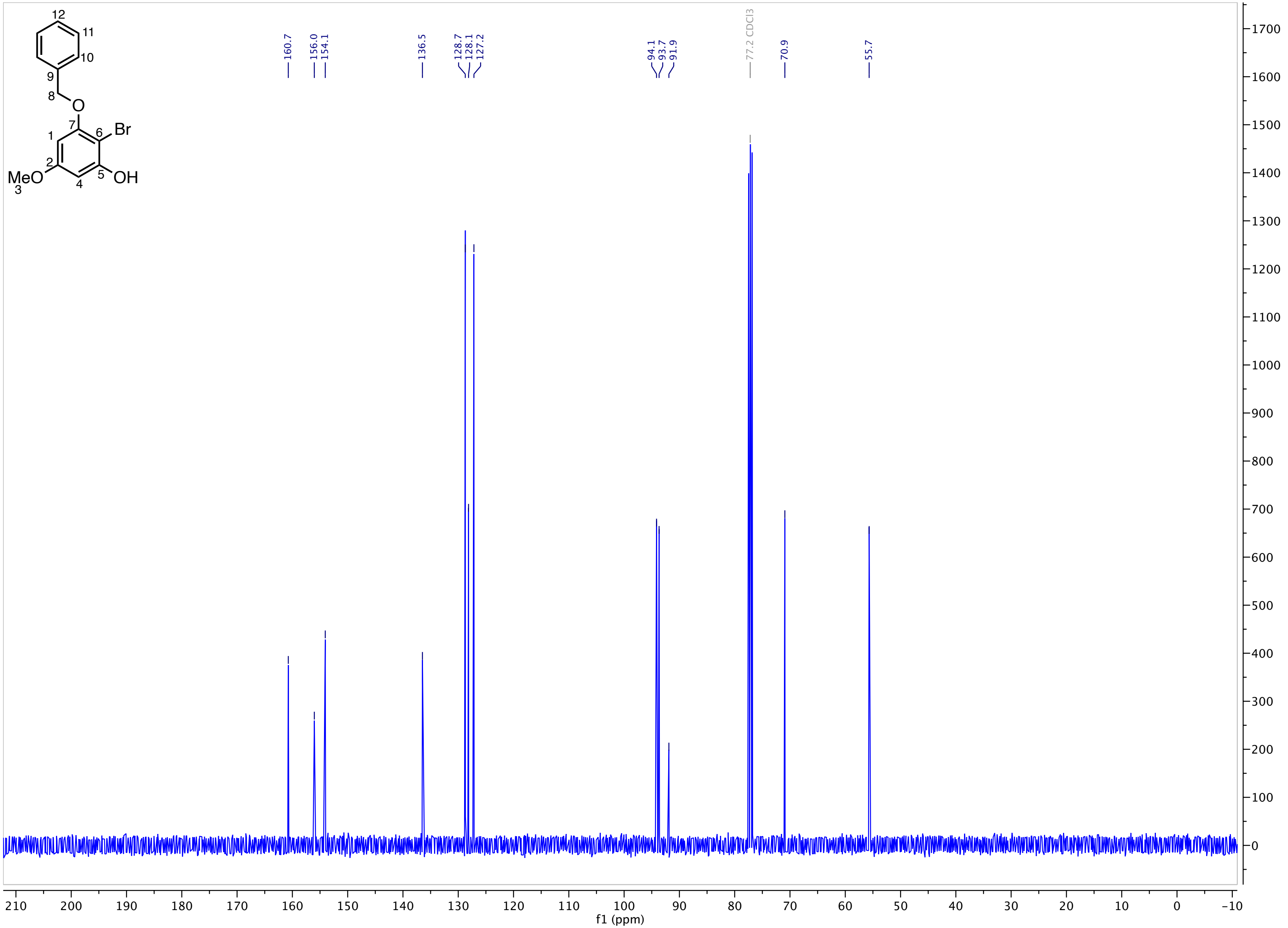

^{1}**H NMR** (500 MHz, $CDCl_3$): 2-Bromo-3-chloro-5-methoxyphenol (**2g**)

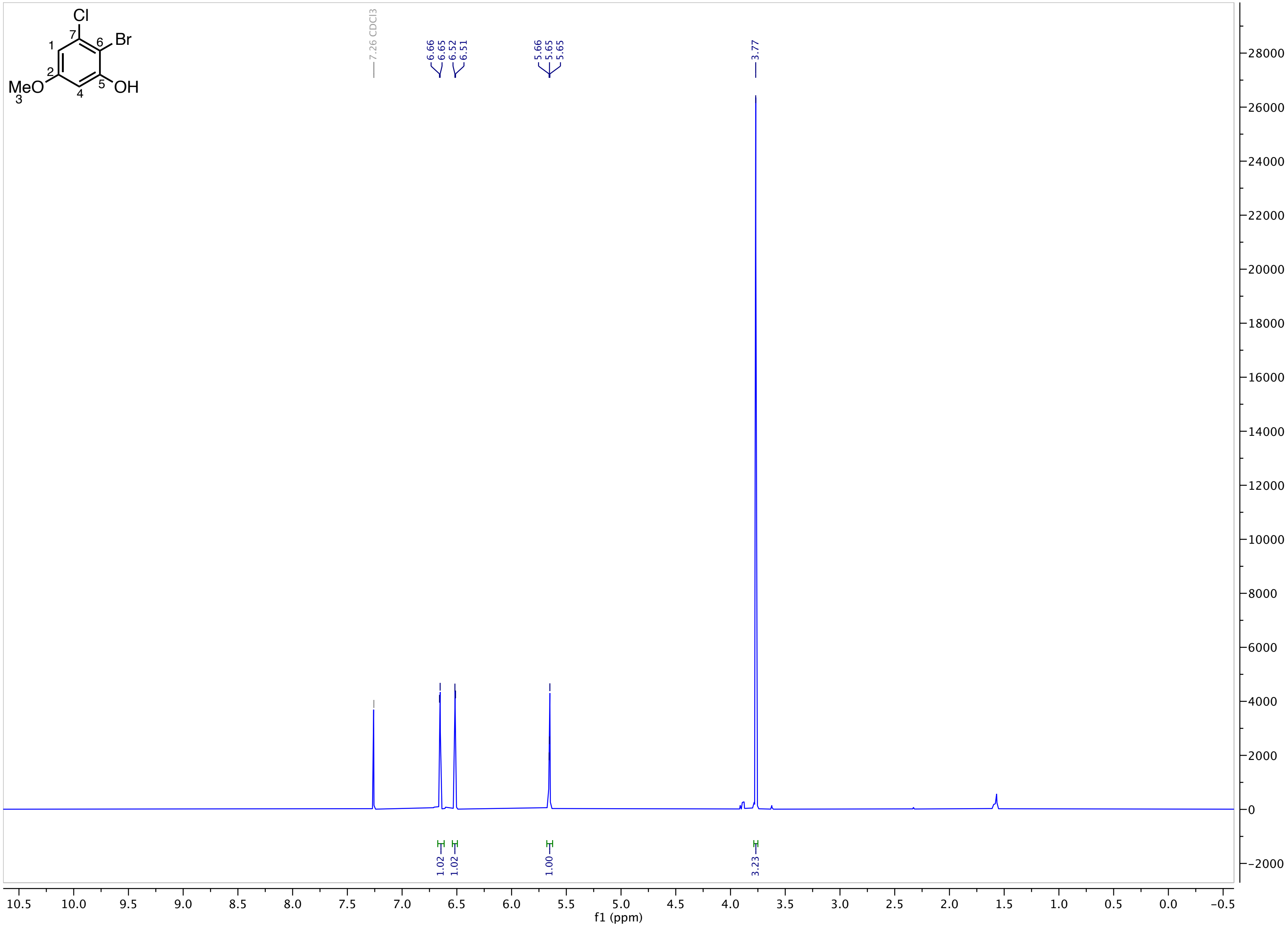

^{13}**C NMR** (126 MHz, $CDCl_3$): 2-Bromo-3-chloro-5-methoxyphenol (**2g**)

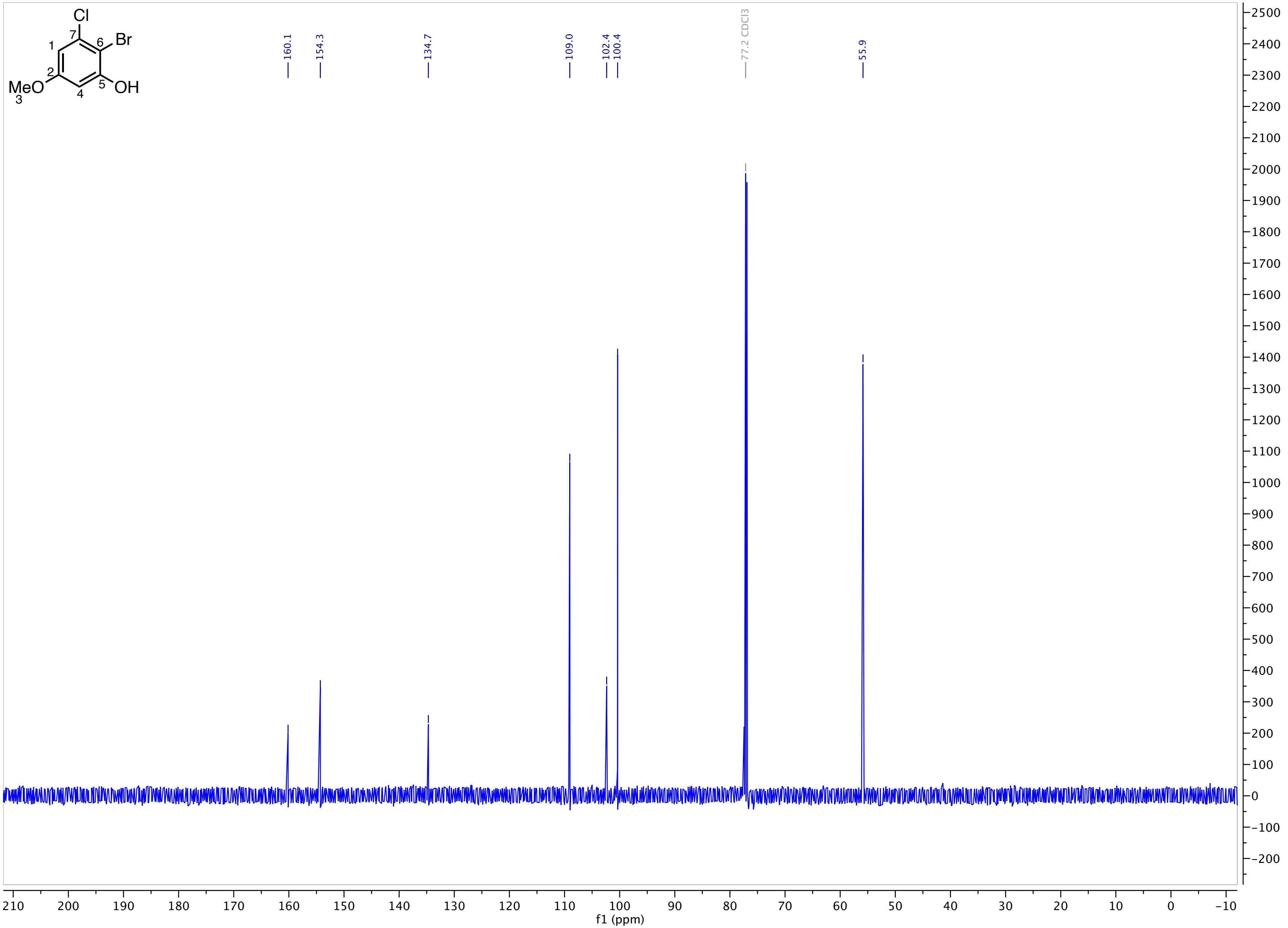

^{1}H NMR (500 MHz, $CDCl_3$): (3a*S*,8a*R*)-2,3,3a,8a-Tetrahydrofuro[2,3-*b*]benzofuran (**3a**)

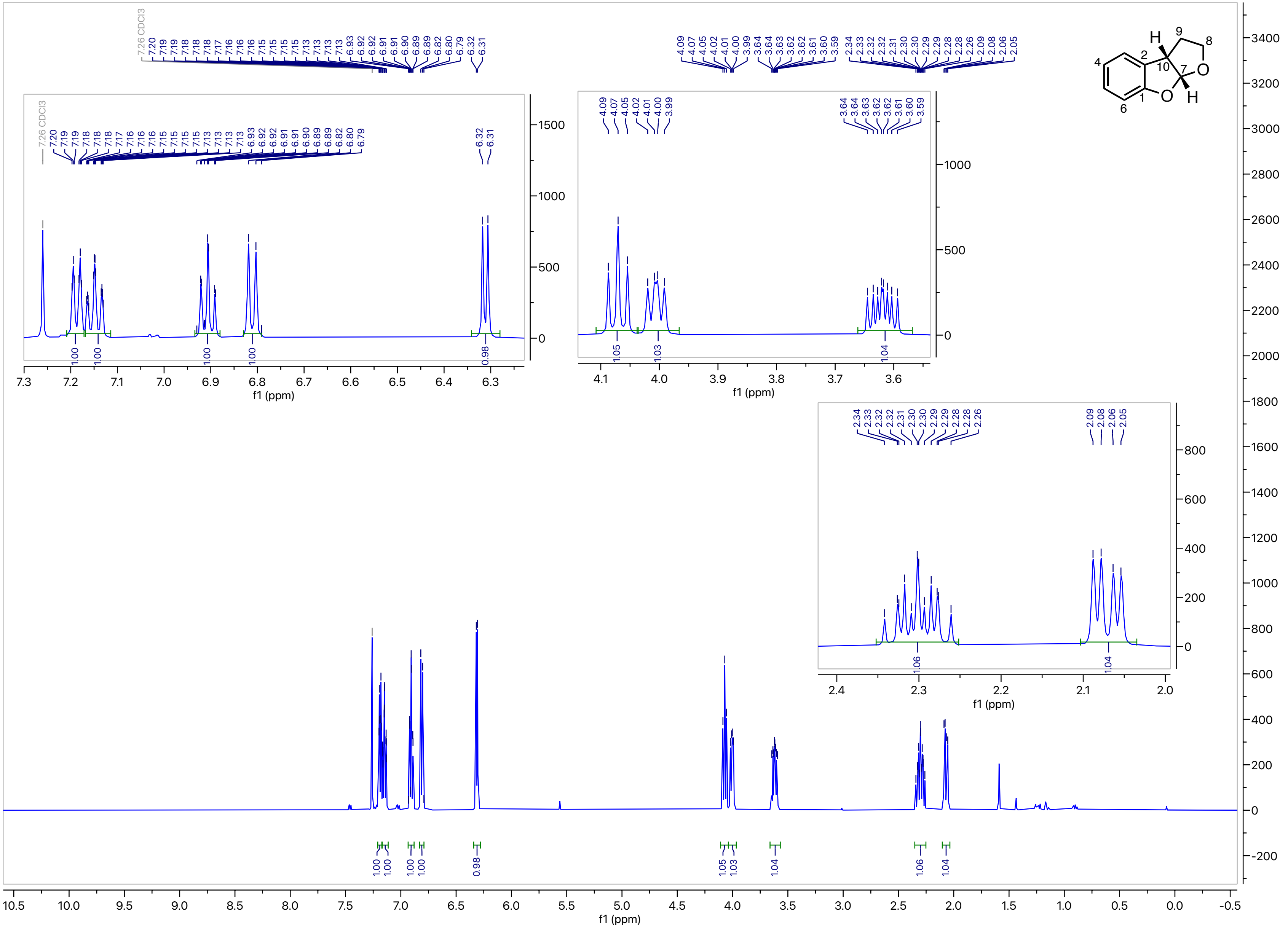

^{13}C NMR (126 MHz, $CDCl_3$): (3a*S*,8a*R*)-2,3,3a,8a-Tetrahydrofuro[2,3-*b*]benzofuran (**3a**)

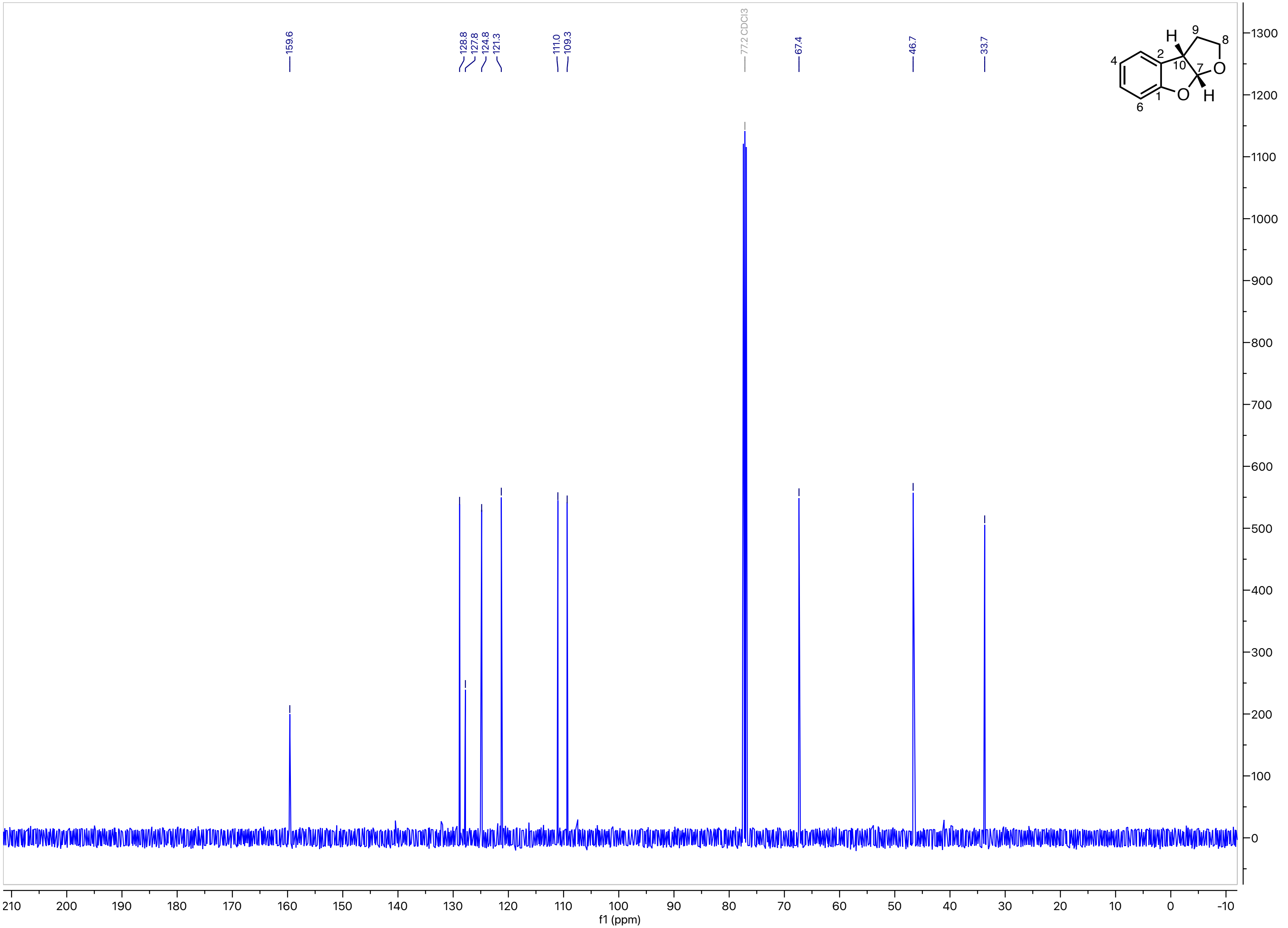

^{1}H NMR (700 MHz, $CDCl_3$): (3a*S*,8a*R*)-5-Methoxy-2,3,3a,8a-tetrahydrofuro[2,3-*b*]benzofuran (**3b**)

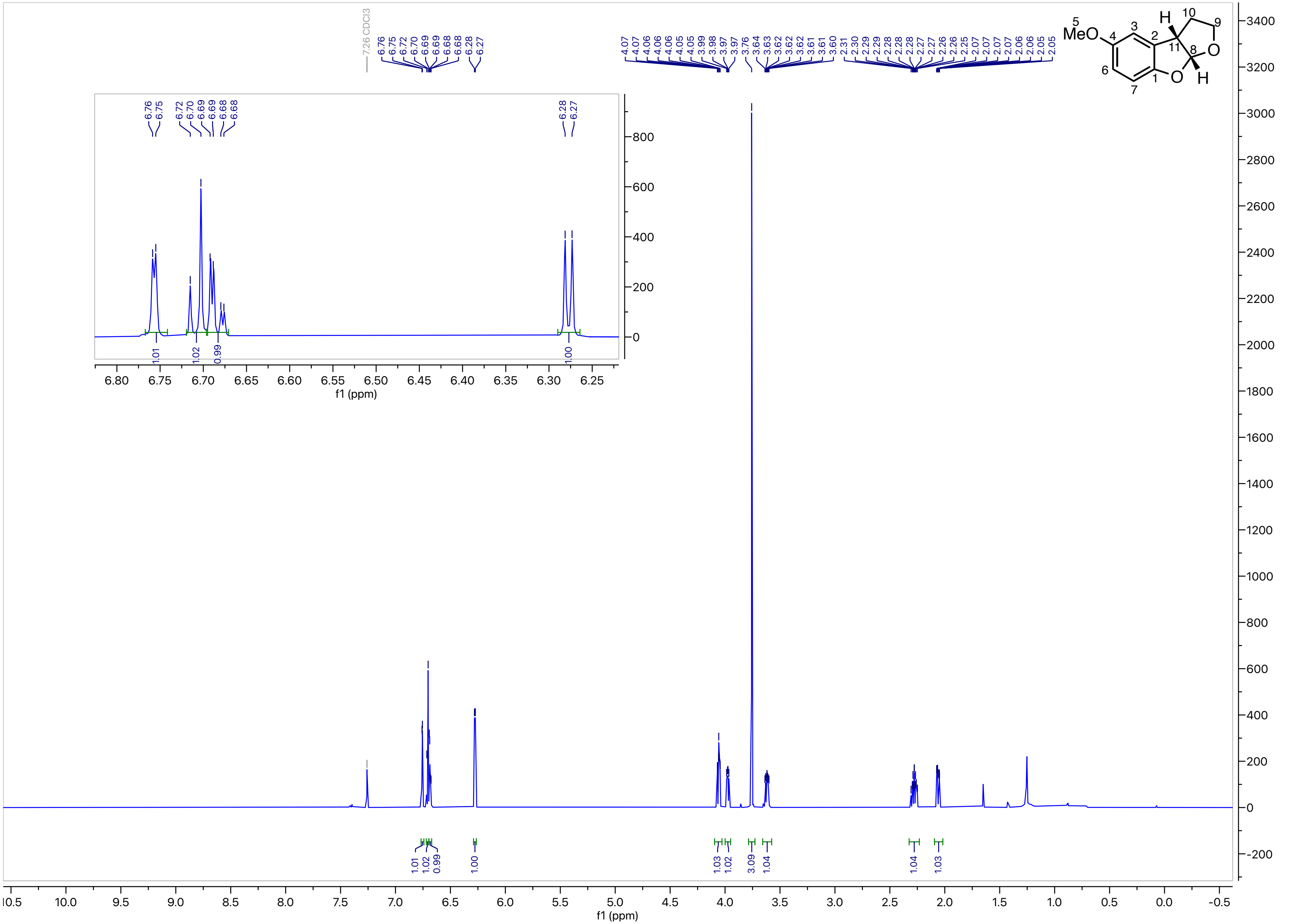

^{13}C NMR (176 MHz, $CDCl_3$): (3a*S*,8a*R*)-5-Methoxy-2,3,3a,8a-tetrahydrofuro[2,3-*b*]benzofuran (**3b**)

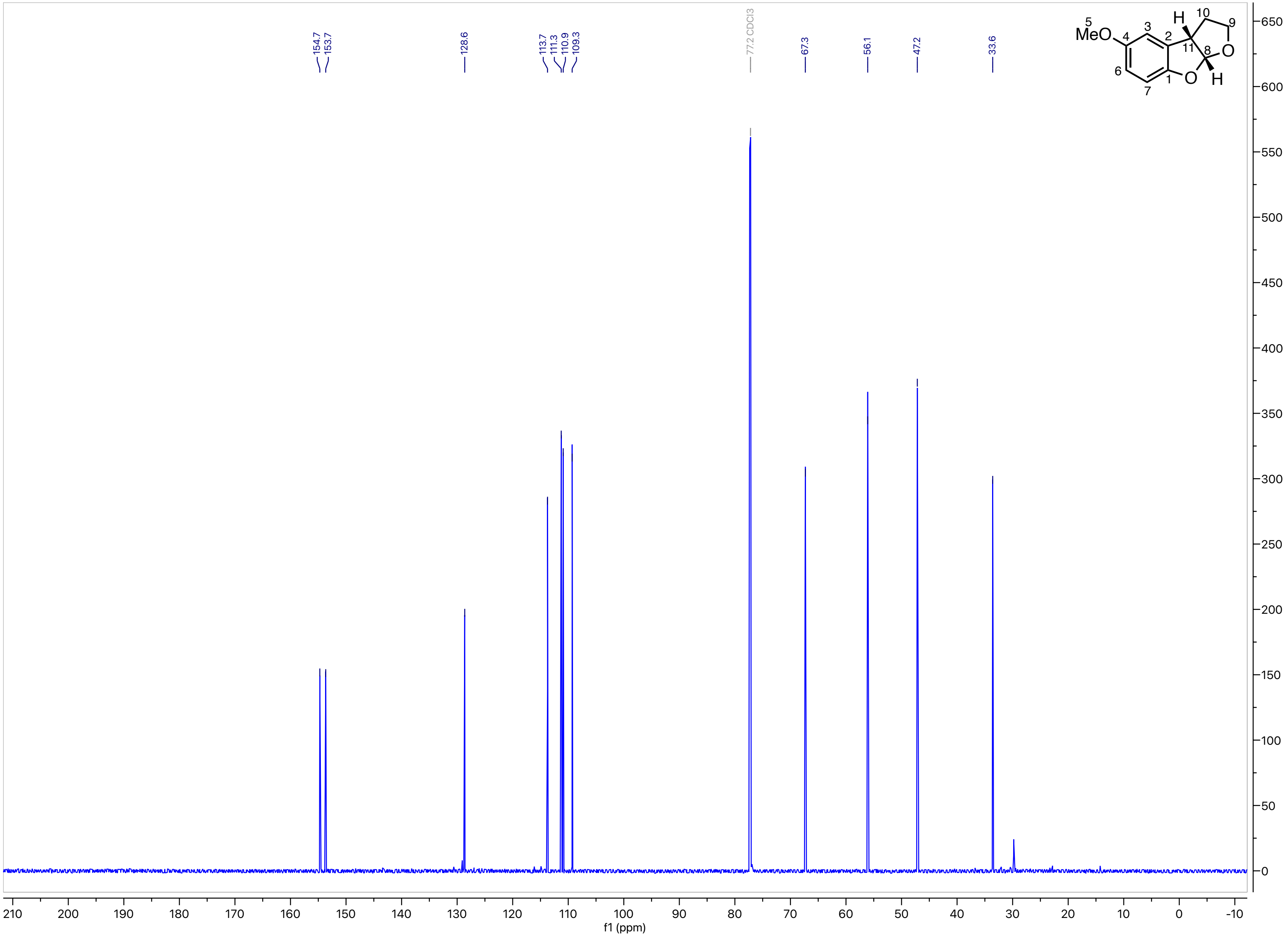

^{1}H NMR (500 MHz, benzene-d_6): (3a*S*,8a*R*)-5-(trifluoromethyl)-2,3,3a,8a-Tetrahydrofuro[2,3-*b*]benzofuran (**3c**)

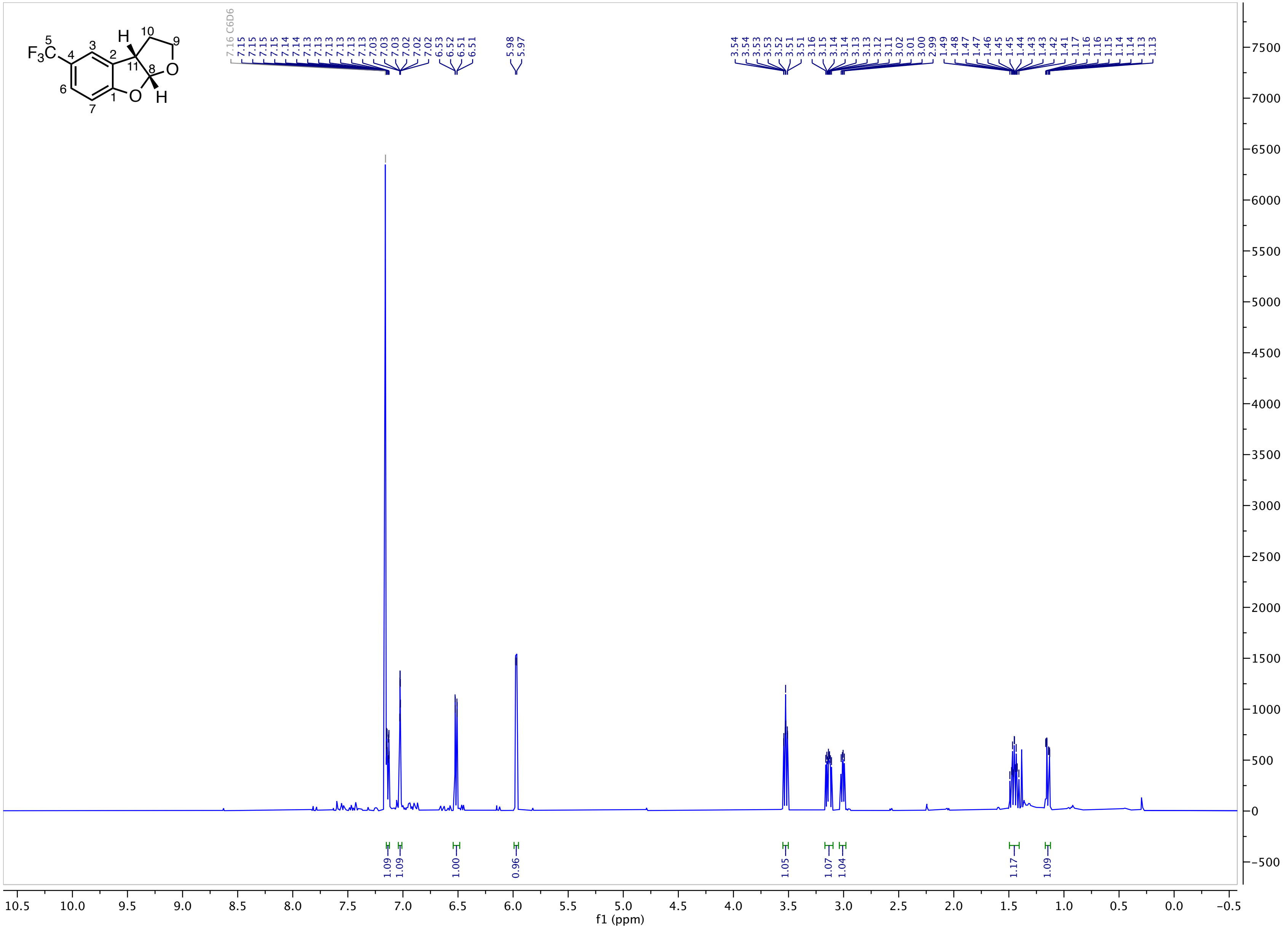

^{13}C NMR (126 MHz, benzene-d_6): (3a*S*,8a*R*)-5-(trifluoromethyl)-2,3,3a,8a-Tetrahydrofuro[2,3-*b*]benzofuran (**3c**)

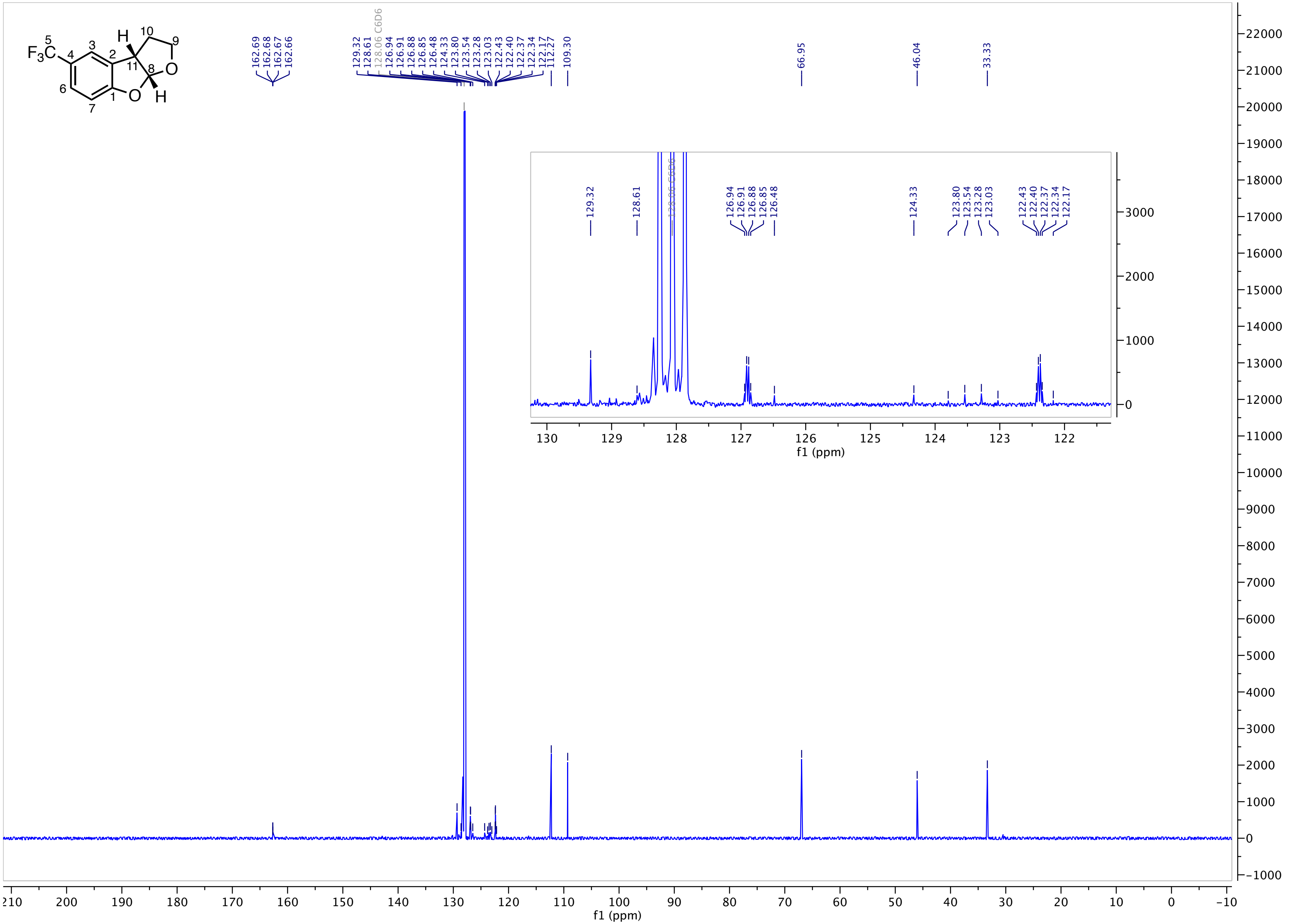

^{19}F NMR (376 MHz, $CDCl_3$): (3a*S*,8a*R*)-5-(trifluoromethyl)-2,3,3a,8a-Tetrahydrofuro[2,3-*b*]benzofuran (**3c**)

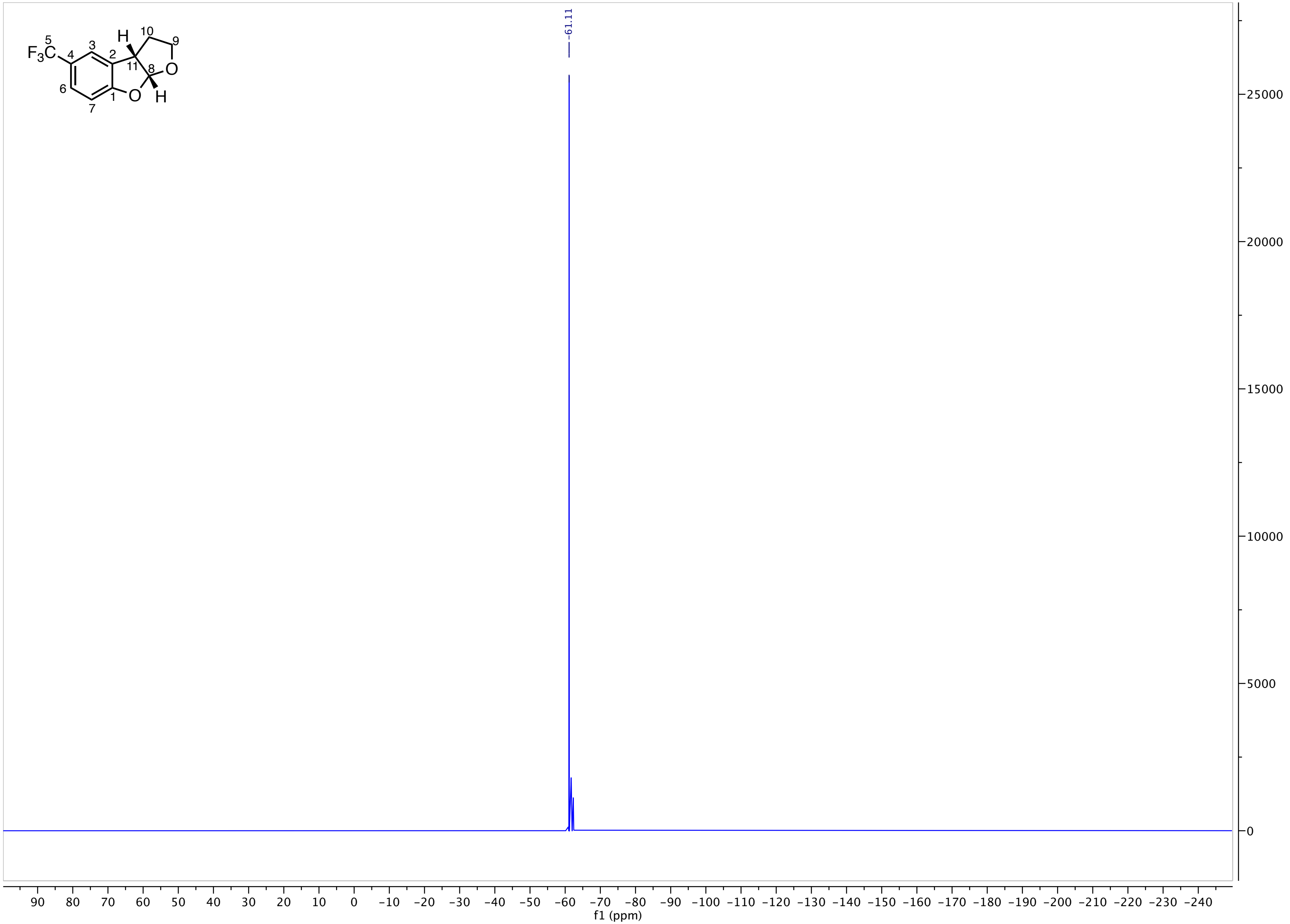

^{13}C NMR (400 MHz, $CDCl_3$): (3a*S*,8a*R*)-4-Methyl-2,3,3a,8a-tetrahydrofuro[2,3-*b*]benzofuran (**3d**)

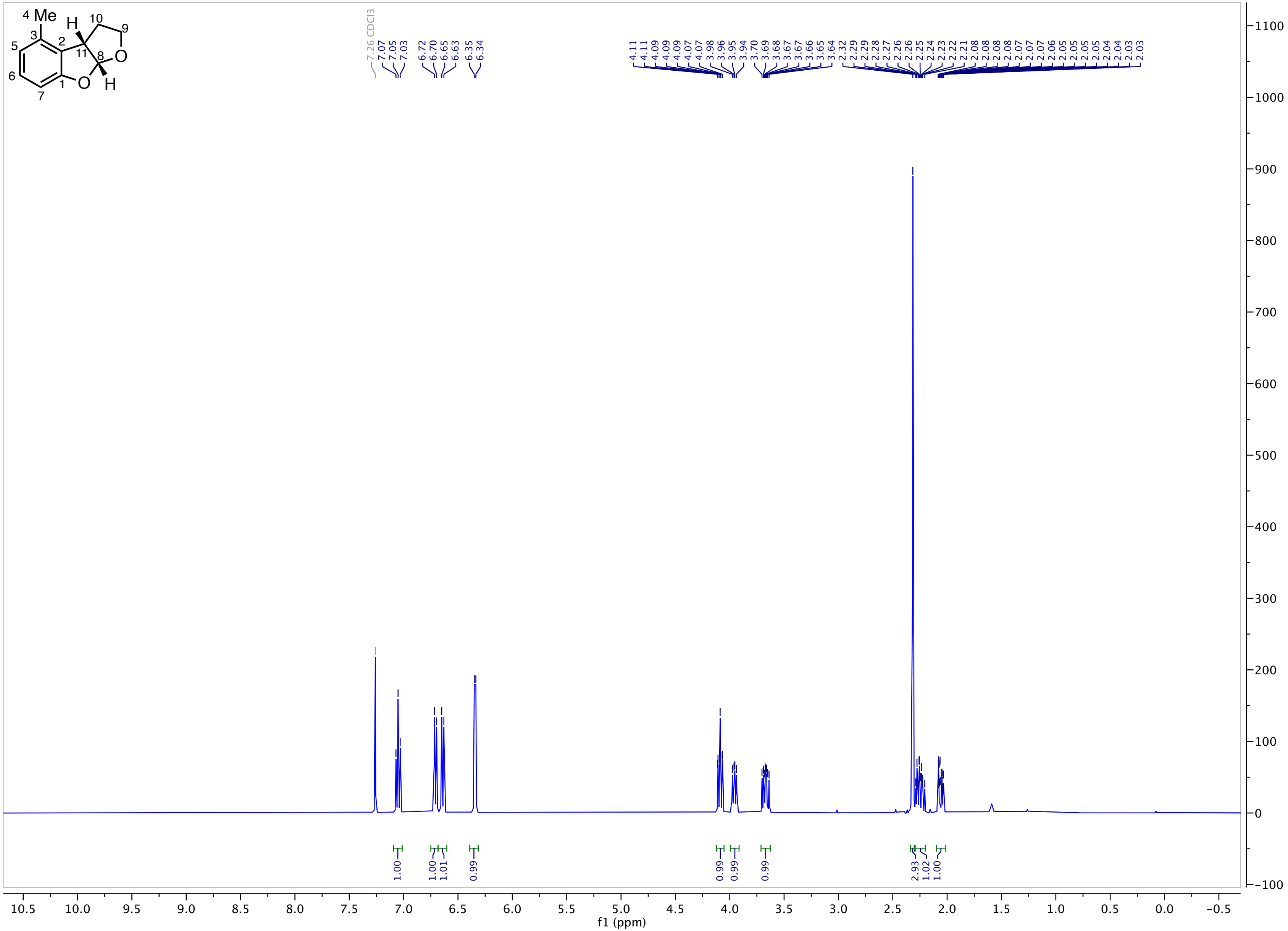

^{13}C NMR (101 MHz, $CDCl_3$): (3a*S*,8a*R*)-4-Methyl-2,3,3a,8a-tetrahydrofuro[2,3-*b*]benzofuran (**3d**)

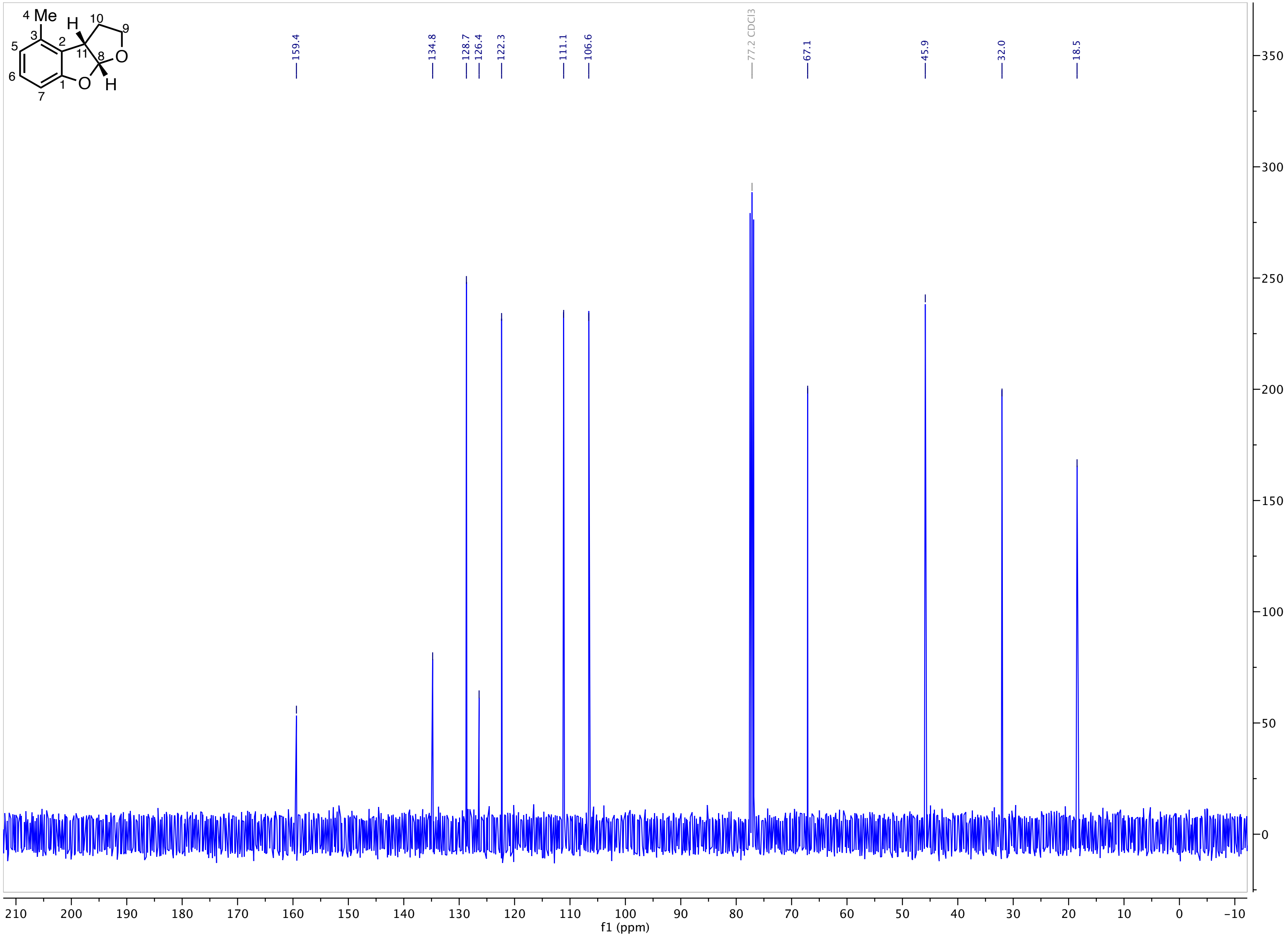

^{1}H NMR (400 MHz, $CDCl_3$): (3a*S*,8a*R*)-4-Chloro-2,3,3a,8a-tetrahydrofuro[2,3-*b*]benzofuran (**3h**)

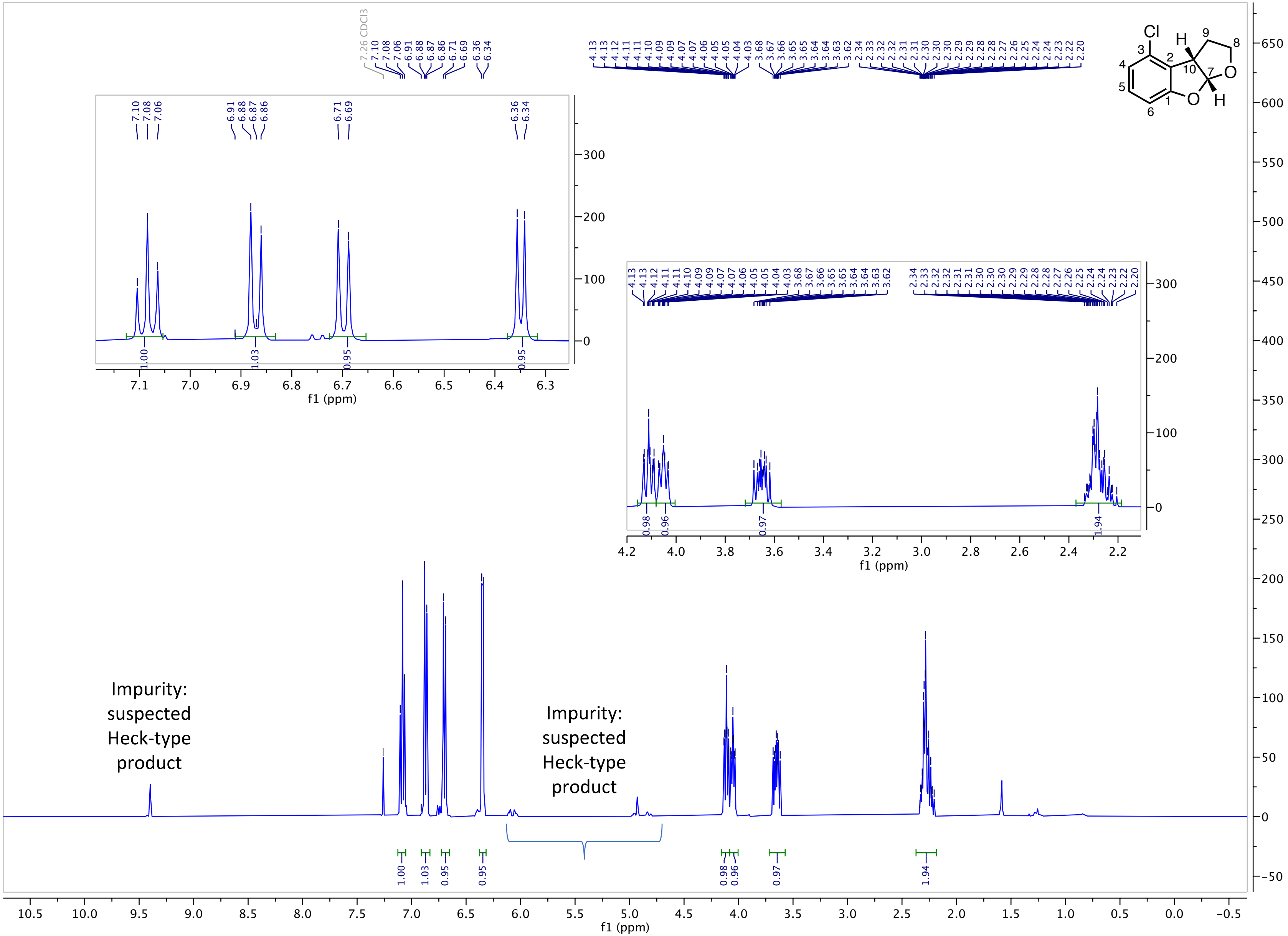

^{13}C NMR (101 MHz, $CDCl_3$): (3a*S*,8a*R*)-4-Chloro-2,3,3a,8a-tetrahydrofuro[2,3-*b*]benzofuran (**3h**)

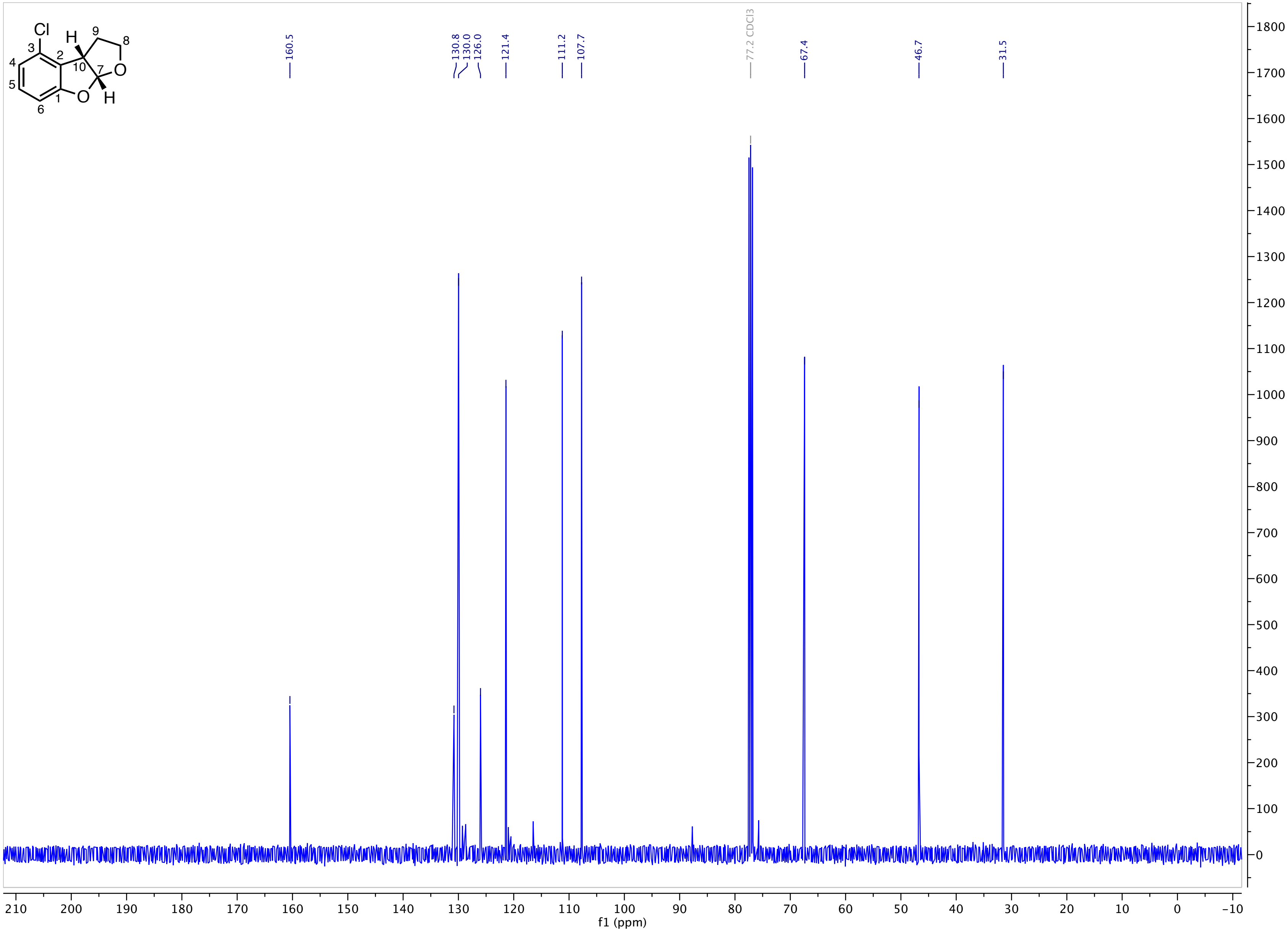

^{1}H NMR (500 MHz, $CDCl_3$): (3a*S*,8a*R*)-4-Chloro-2,3,3a,8a-tetrahydrofuro[2,3-*b*]benzofuran-7-ol (**S5**)

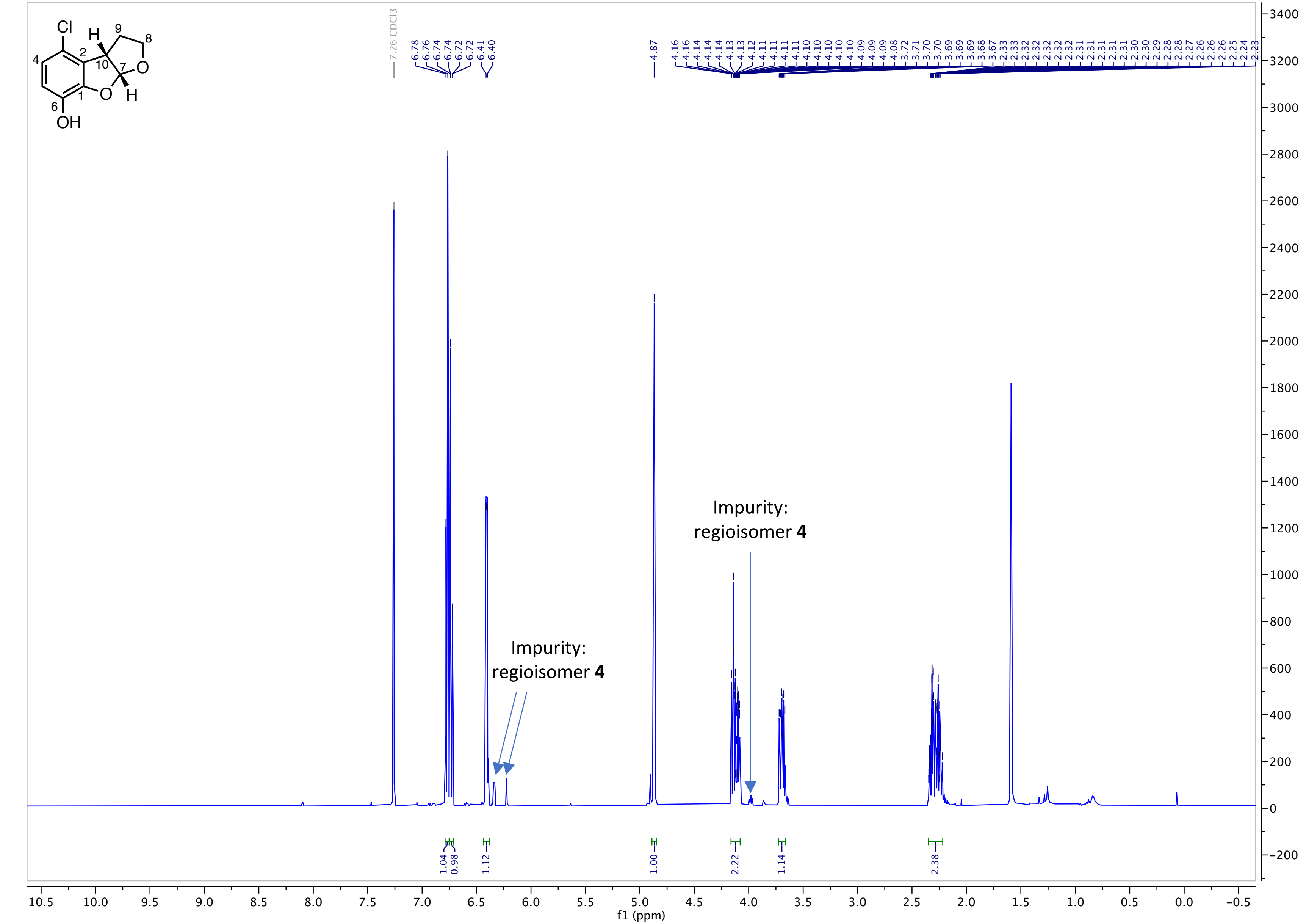

^{13}C NMR (176 MHz $CDCl_3$): (3a*S*,8a*R*)-4-Chloro-2,3,3a,8a-tetrahydrofuro[2,3-*b*]benzofuran-7-ol (**S5**)

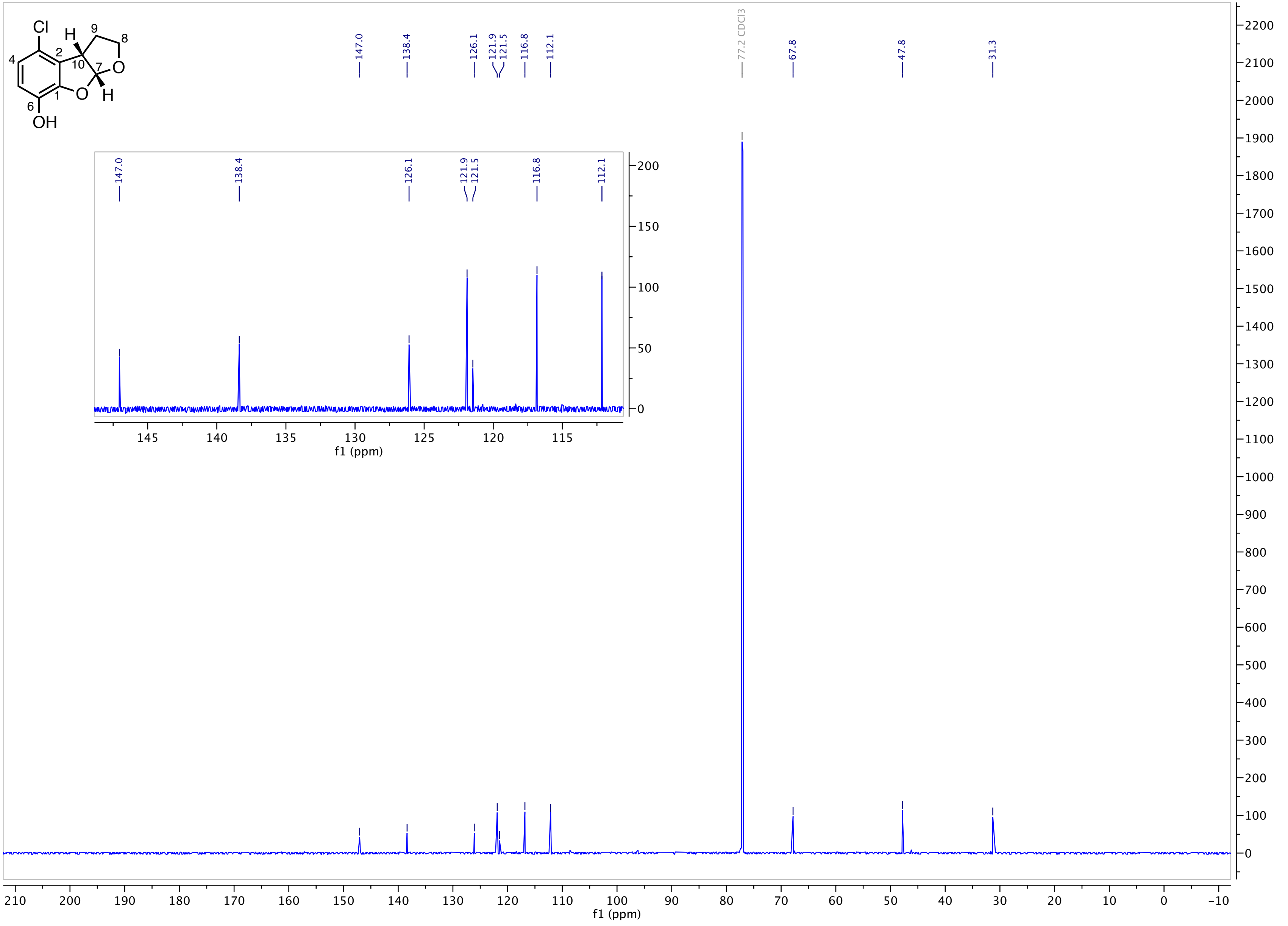

^{1}H NMR (500 MHz, $CDCl_3$): (3a*S*,8a*R*)-4-Chloro-2,3,3a,8a-tetrahydrofuro[2,3-*b*]benzofuran-6-ol (**4**)

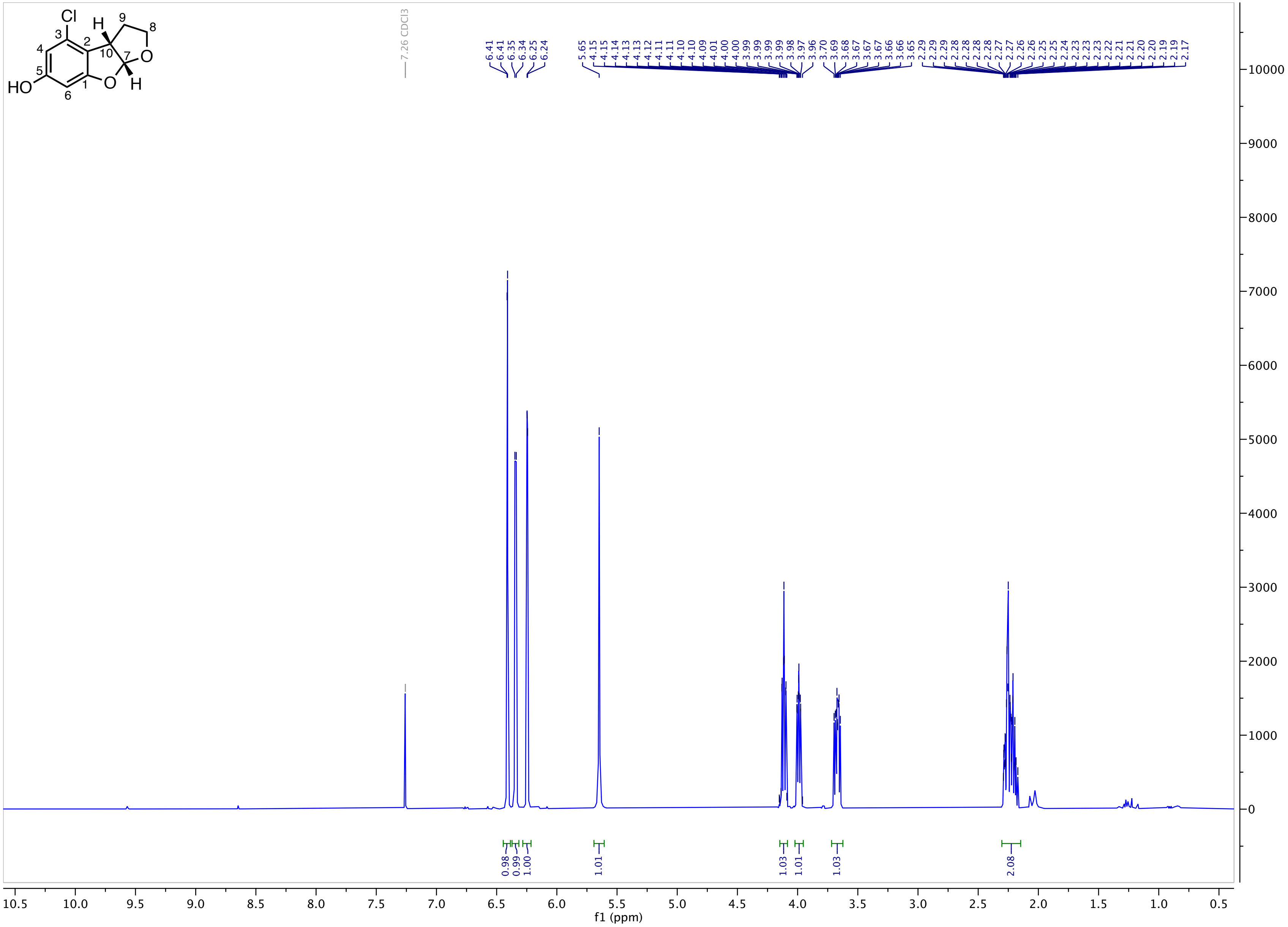

^{13}C NMR (126 MHz, $CDCl_3$): (3a*S*,8a*R*)-4-Chloro-2,3,3a,8a-tetrahydrofuro[2,3-*b*]benzofuran-6-ol (**4**)

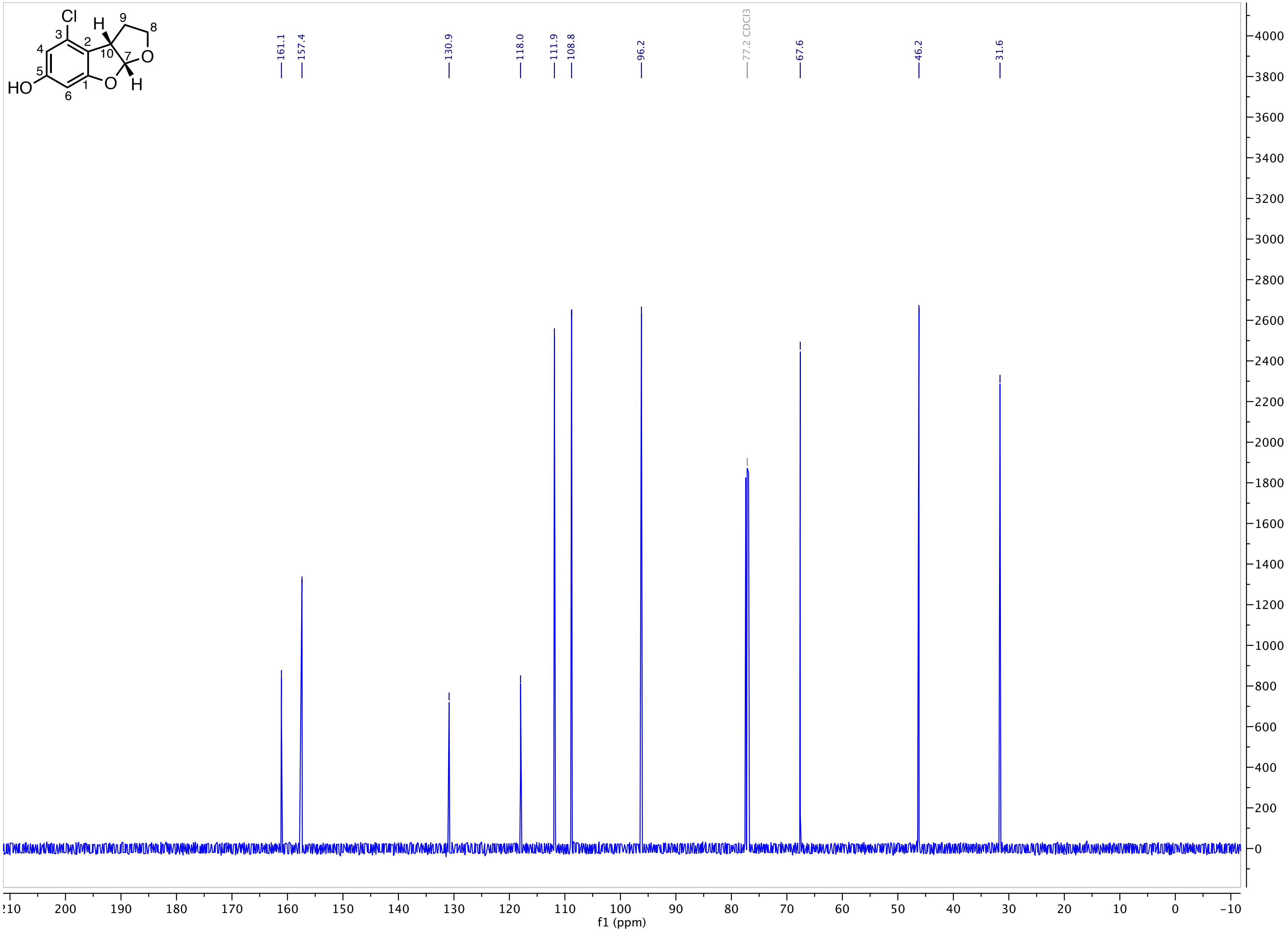

^{1}H NMR (500 MHz, $CDCl_3$): (3a*S*,8a*R*)-4-Chloro-6-methoxy-2,3,3a,8a-tetrahydrofuro[2,3-*b*]benzofuran (**5**)

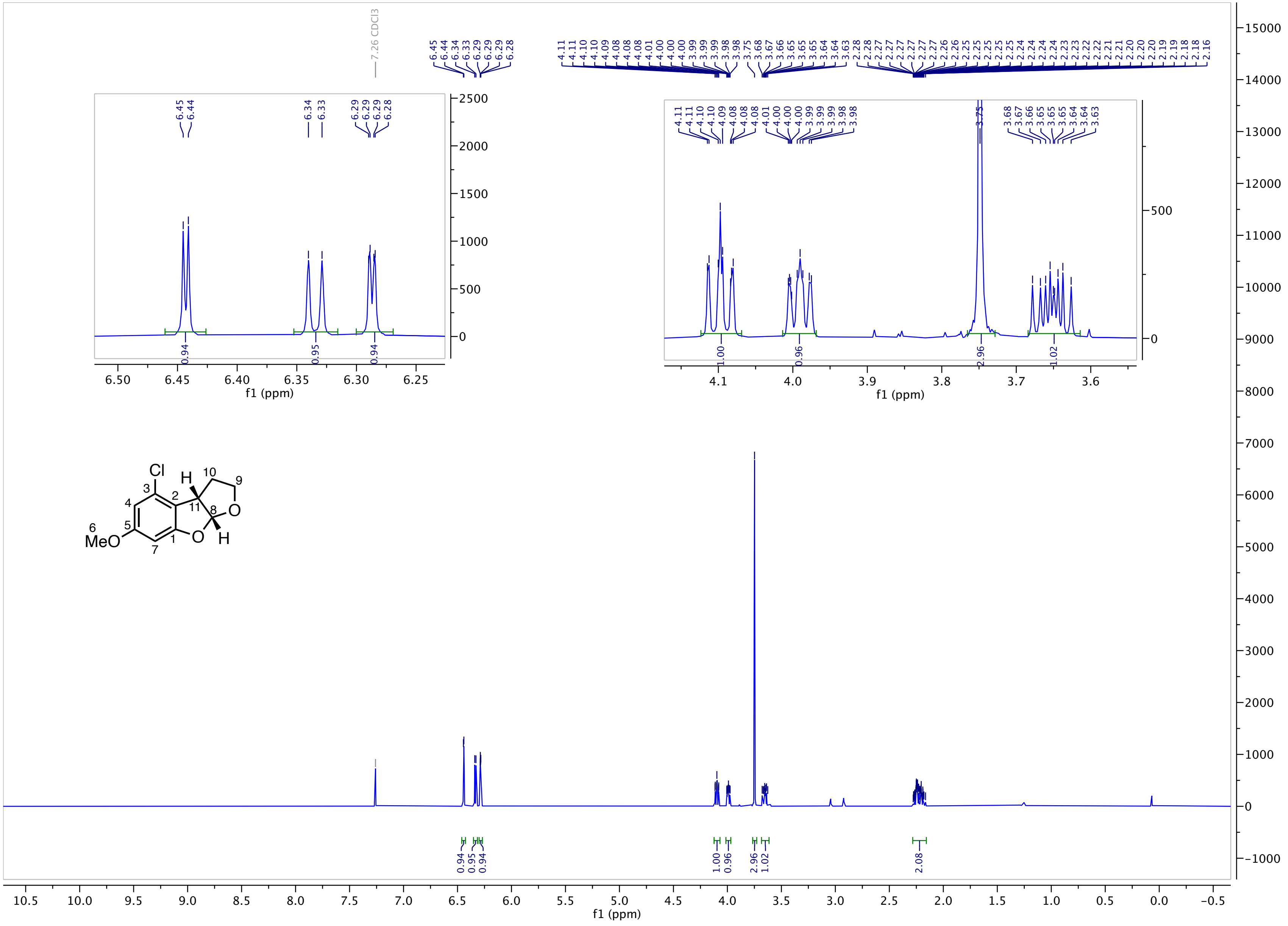

^{13}C NMR (126 MHz, $CDCl_3$): (3a*S*,8a*R*)-4-Chloro-6-methoxy-2,3,3a,8a-tetrahydrofuro[2,3-*b*]benzofuran (**5**)

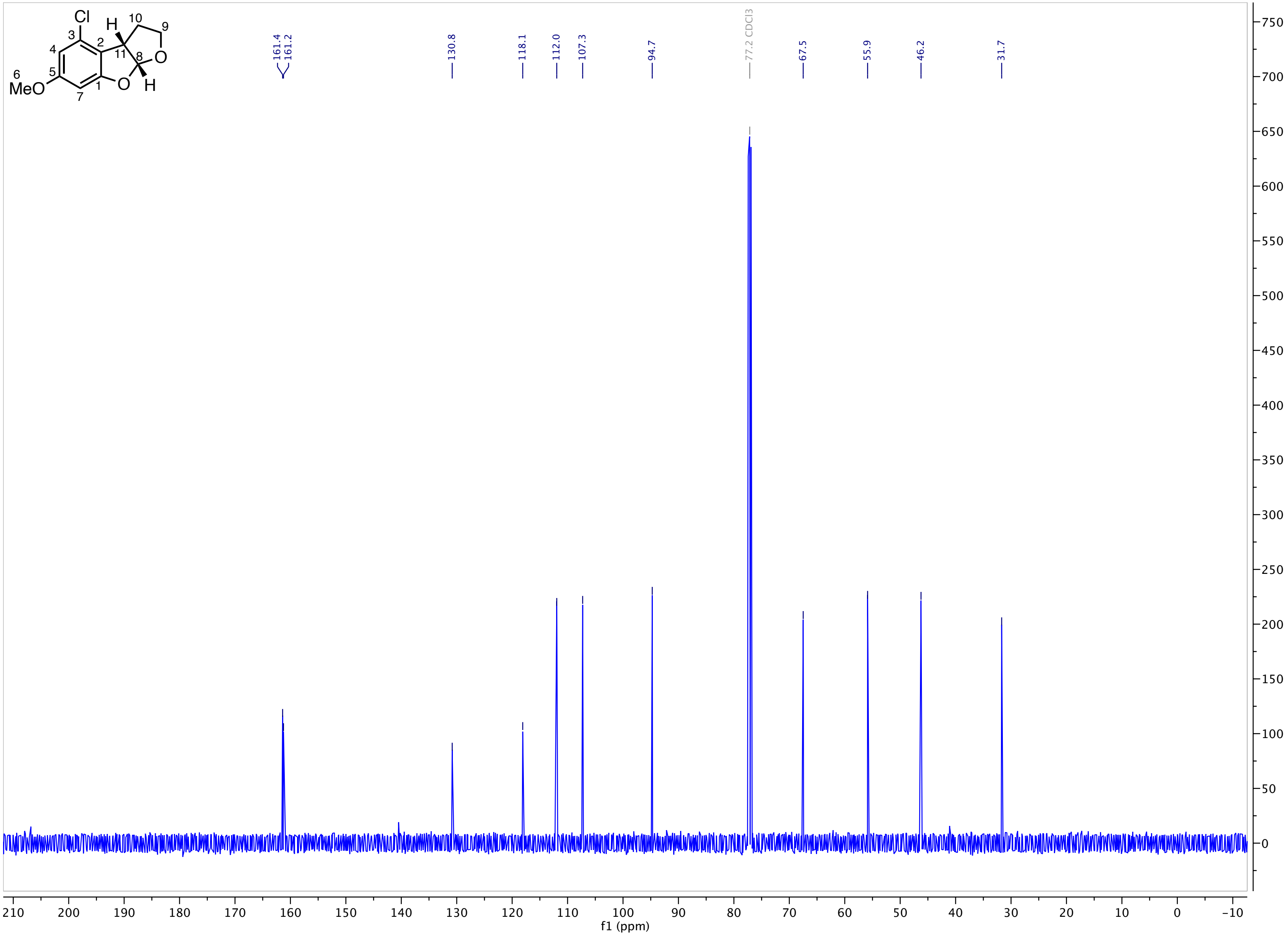

^{1}H NMR (700 MHz, $CDCl_3$): (3a*S*,8a*R*)-6-Methoxy-2,3,3a,8a-tetrahydrofuro[2,3-*b*]benzofuran-4-ol (**1**)

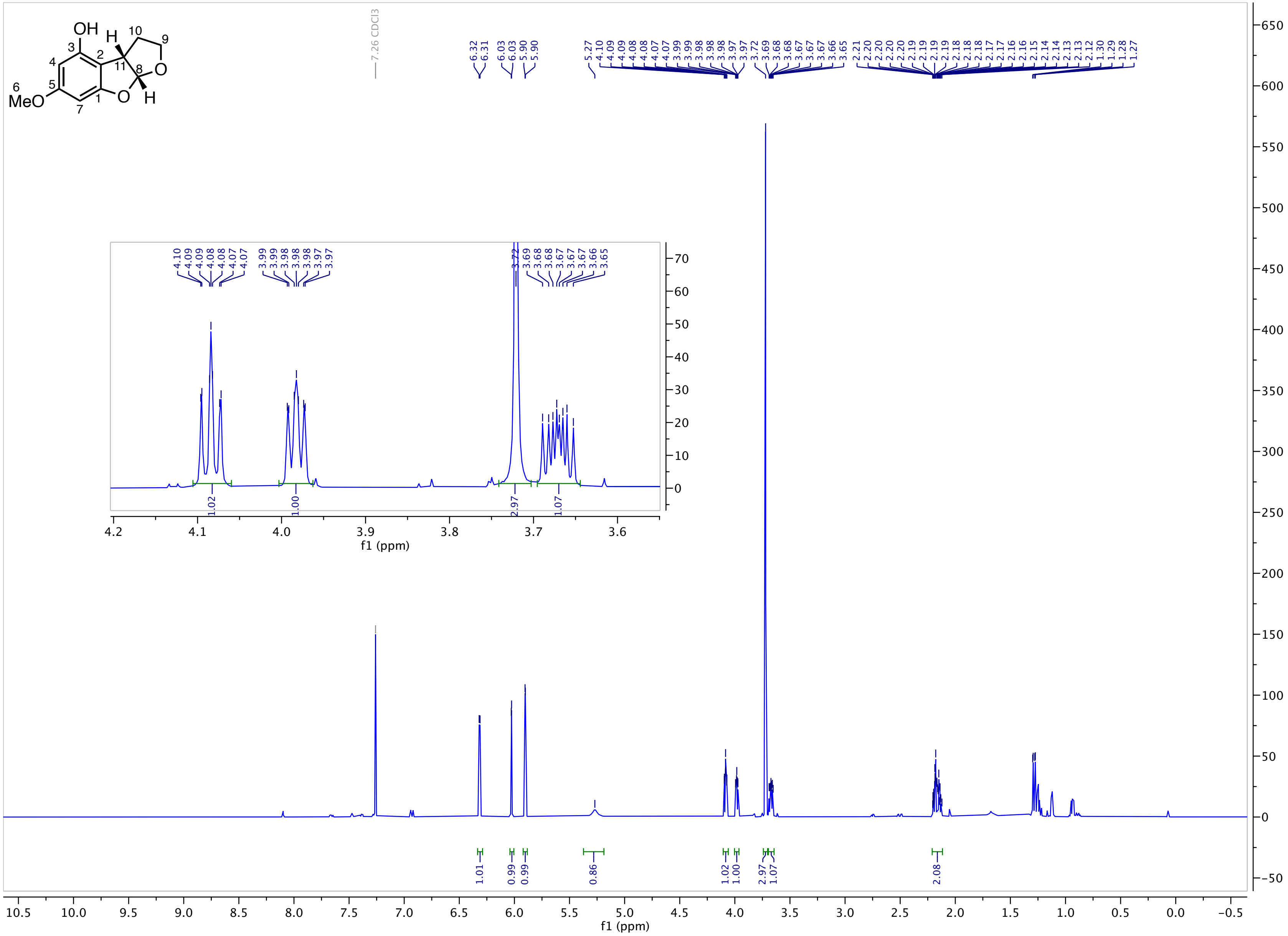

^{13}C NMR (176 MHz, $CDCl_3$): (3a*S*,8a*R*)-6-Methoxy-2,3,3a,8a-tetrahydrofuro[2,3-*b*]benzofuran-4-ol (**1**)

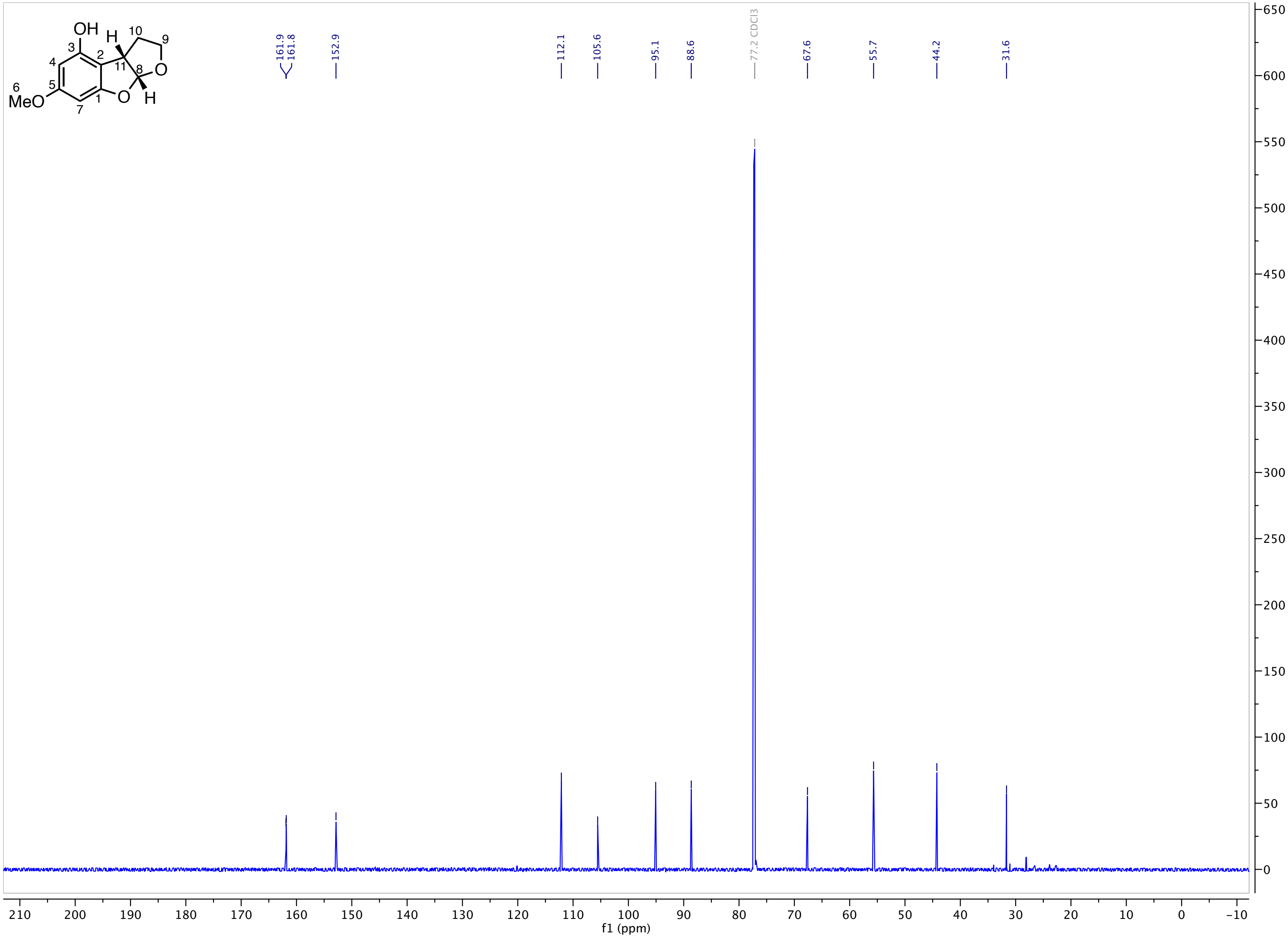

^{1}H NMR (500 MHz, $CDCl_3$): (3a*S*,8a*S*)-8-Tosyl-3,3a,8,8a-tetrahydro-2*H*-furo[2,3-*b*]indole (**7a**)

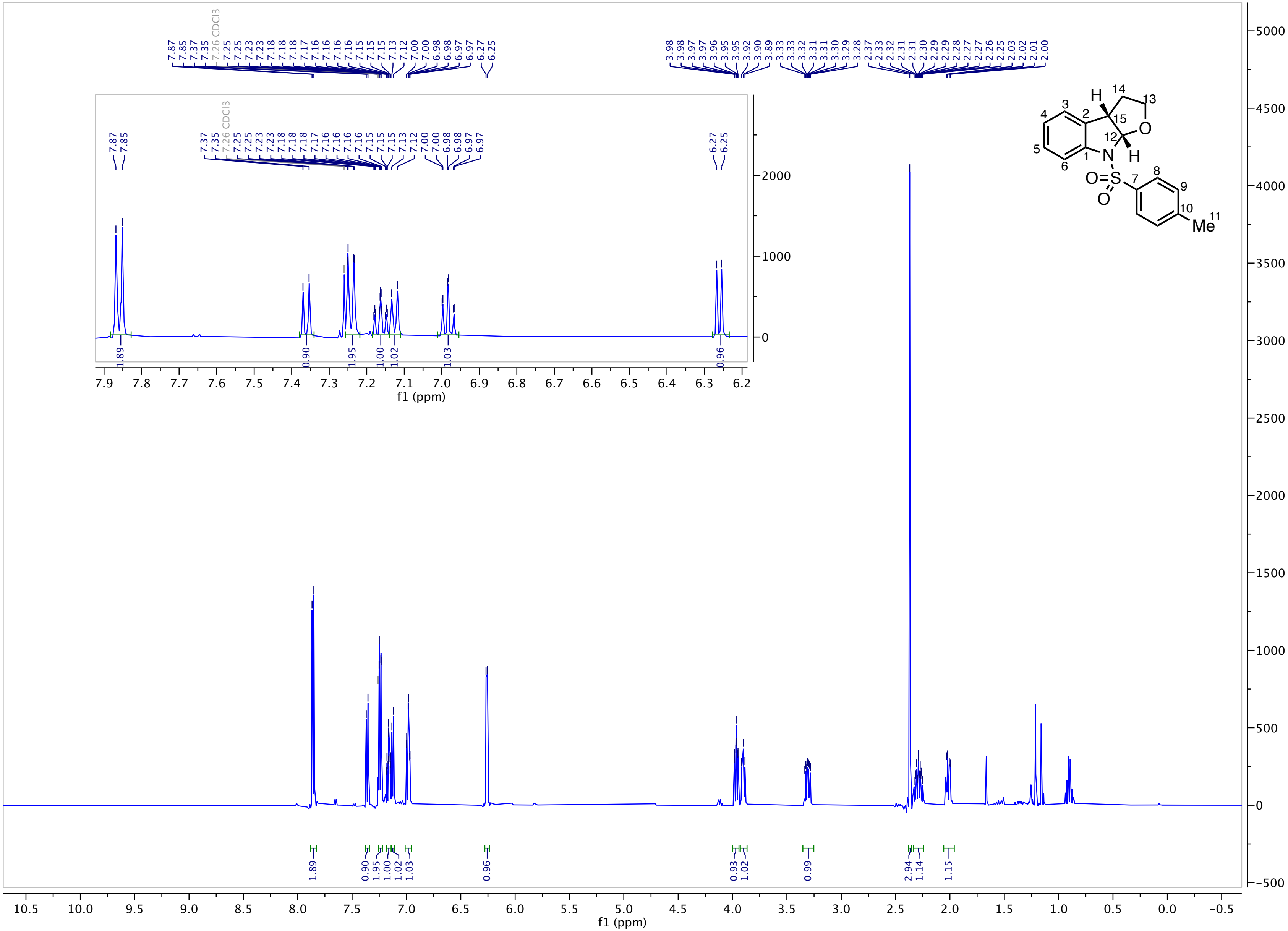

^{13}C NMR (126 MHz, $CDCl_3$): (3a*S*,8a*S*)-8-Tosyl-3,3a,8,8a-tetrahydro-2*H*-furo[2,3-*b*]indole (**7a**)

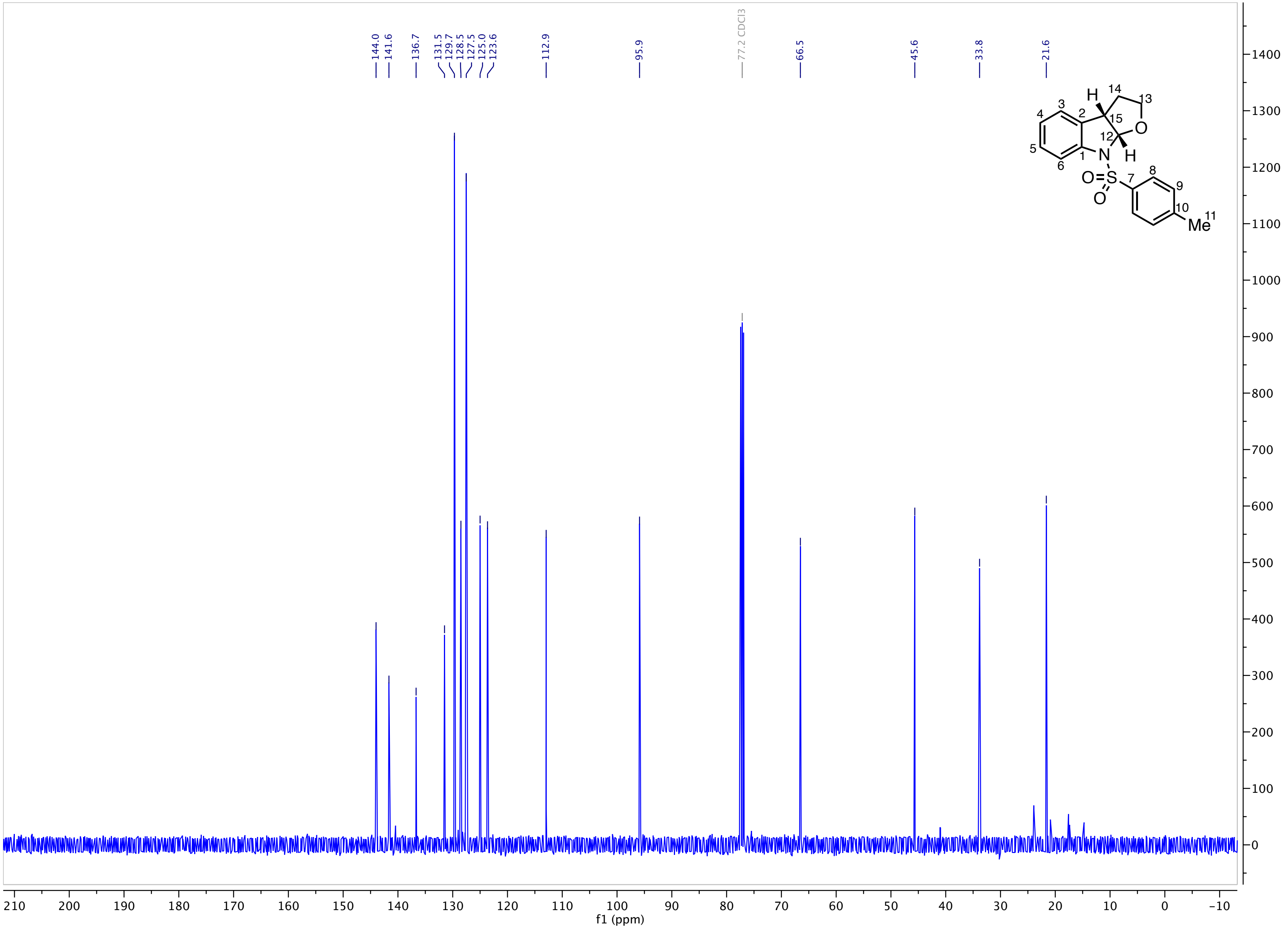

^{1}H NMR (500 MHz, $CDCl_3$): (3a*S*,8a*S*)-5-Methoxy-8-tosyl-3,3a,8,8a-tetrahydro-2*H*-furo[2,3-*b*]indole (**7b**)

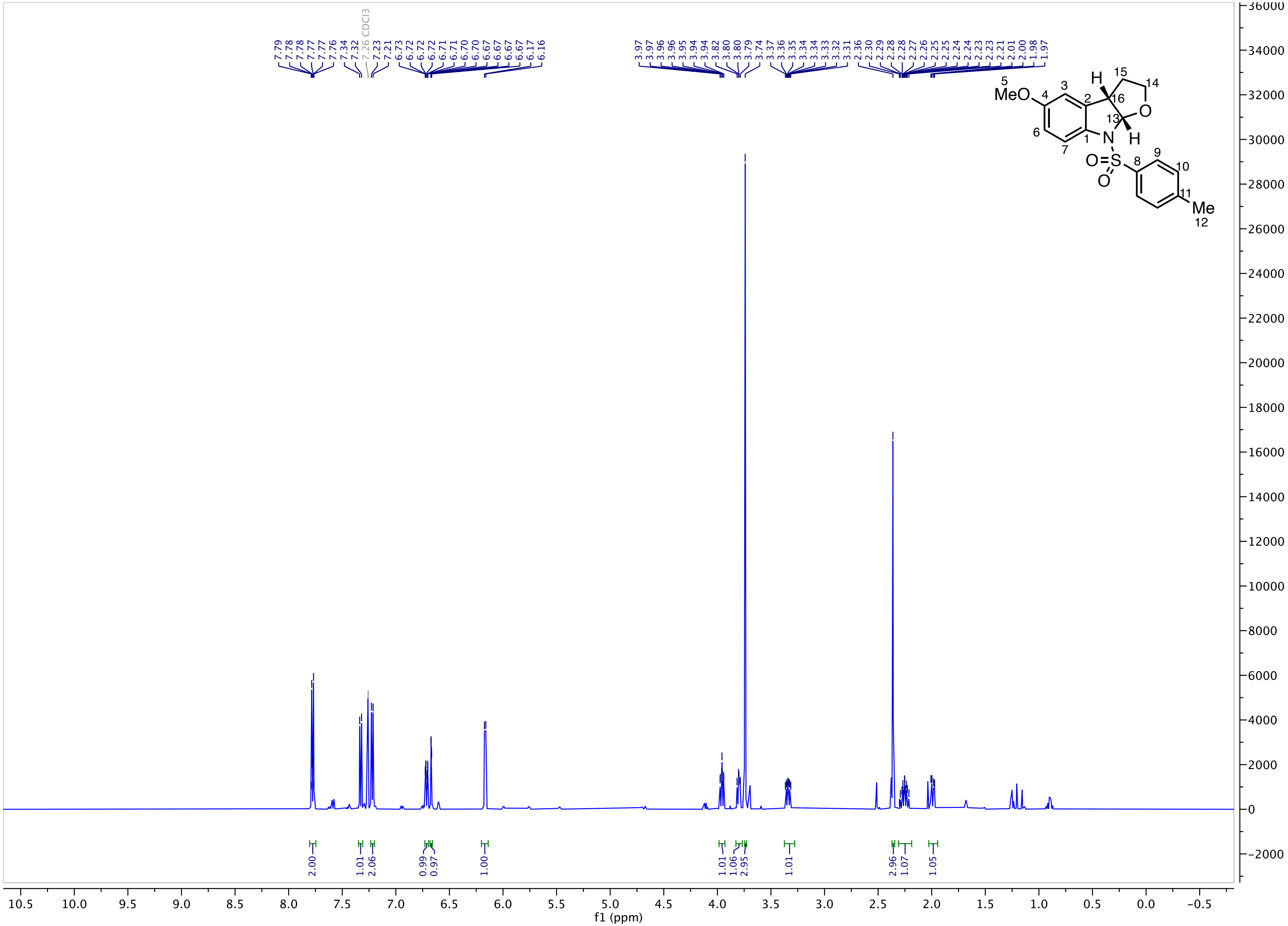

^{13}**C NMR** (126 MHz, $CDCl_3$): (3a*S*,8a*S*)-5-Methoxy-8-tosyl-3,3a,8,8a-tetrahydro-2*H*-furo[2,3-*b*]indole (**7b**)

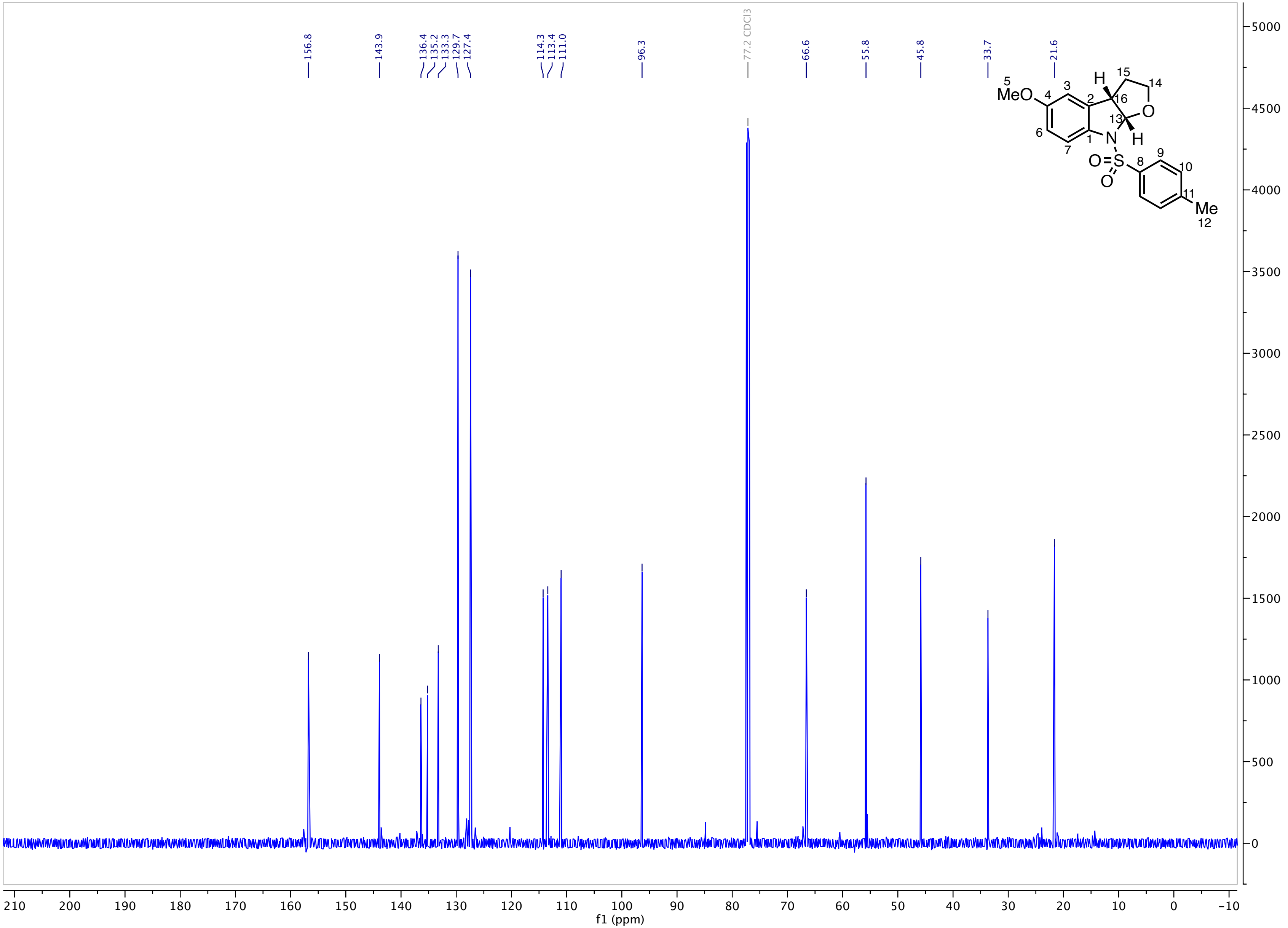

^{1}H NMR (700 MHz, $CDCl_3$): (3a*S*,8a*S*)-8-Tosyl-5-(trifluoromethyl)-3,3a,8,8a-tetrahydro-2*H*-furo[2,3-*b*]indole (**7c**)

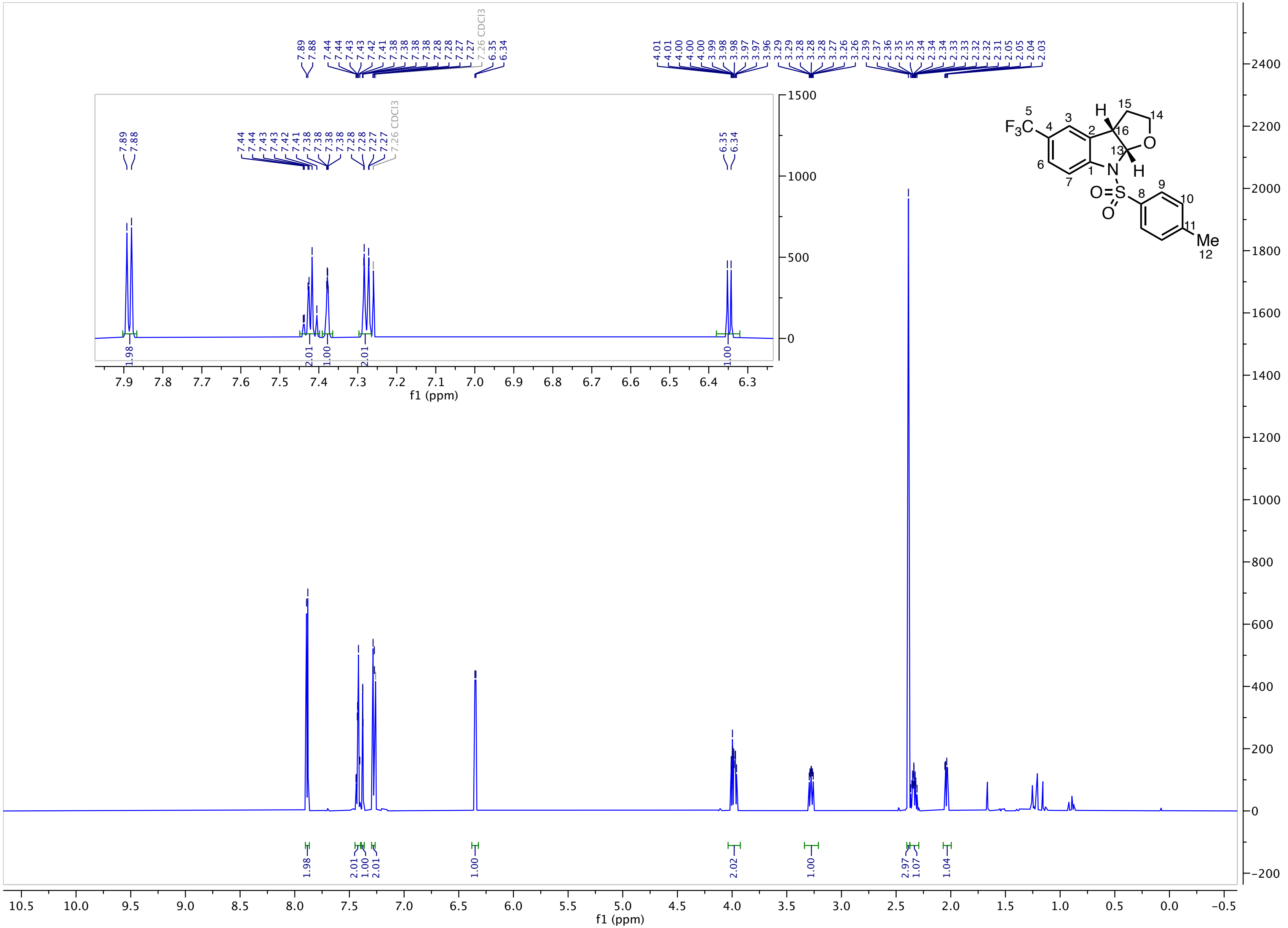

^{13}C NMR (176 MHz, $CDCl_3$): (3a*S*,8a*S*)-8-Tosyl-5-(trifluoromethyl)-3,3a,8,8a-tetrahydro-2*H*-furo[2,3-*b*]indole (**7c**)

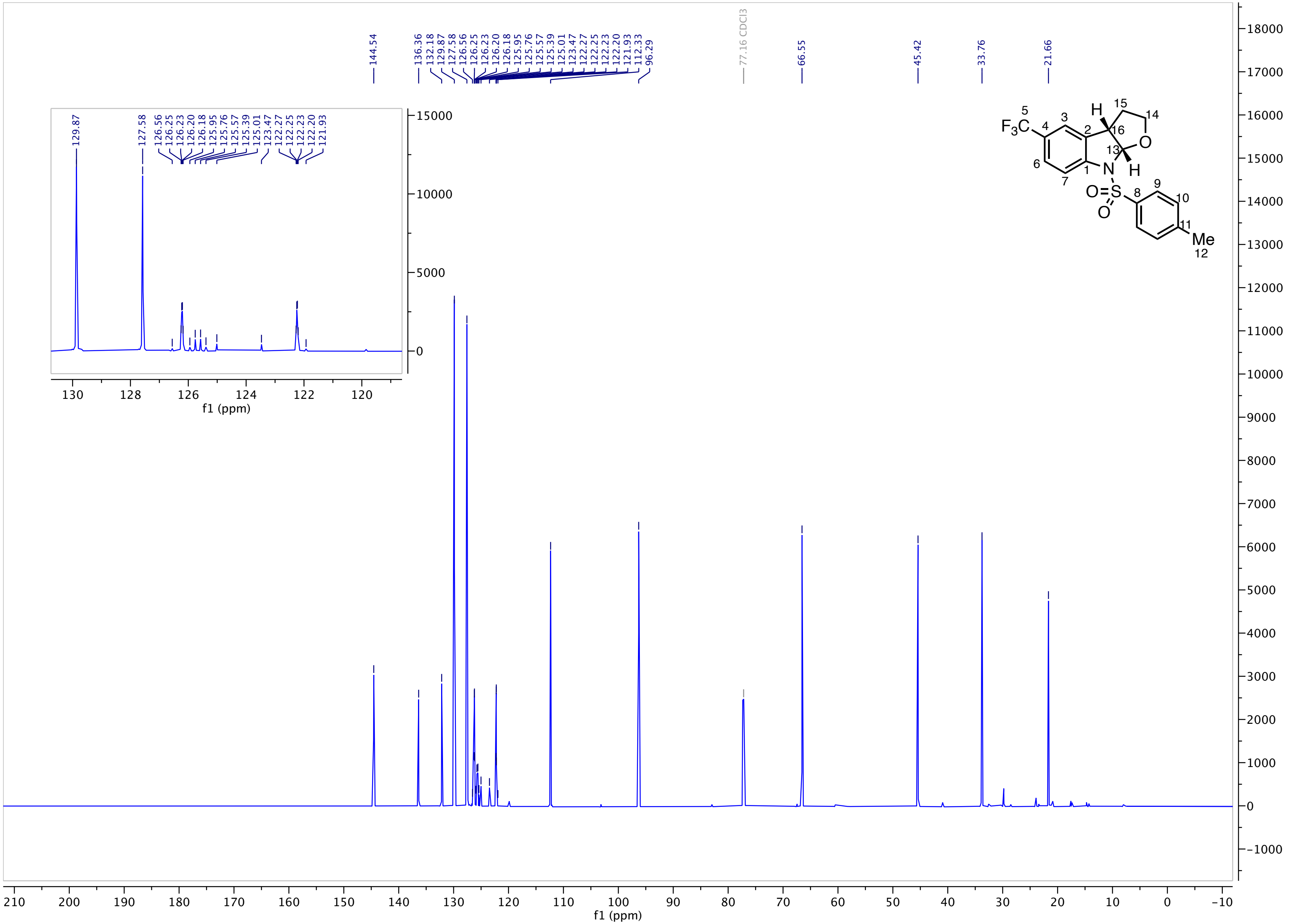

^{19}F NMR (376 MHz, $CDCl_3$): (3a*S*,8a*S*)-8-Tosyl-5-(trifluoromethyl)-3,3a,8,8a-tetrahydro-2*H*-furo[2,3-*b*]indole (**7c**)

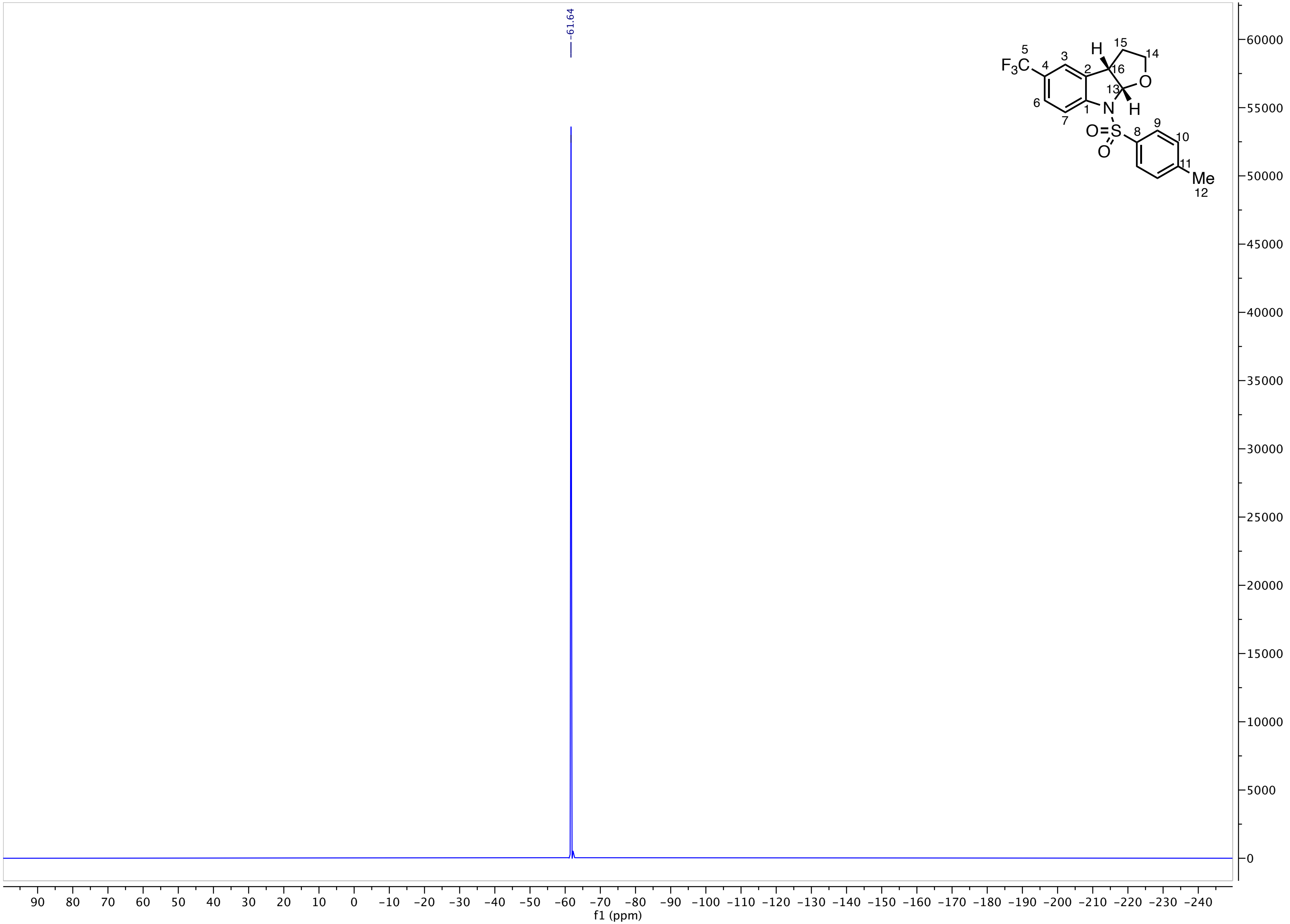

Supplement: Supplementary file 1 [file ol5c02618_si_001.pdf]
